# The willingness to participate in biomedical research involving human beings in low‐ and middle‐income countries: a systematic review

**DOI:** 10.1111/tmi.13195

**Published:** 2019-01-08

**Authors:** Joyce L. Browne, Connie O. Rees, Johannes J. M. van Delden, Irene Agyepong, Diederick E. Grobbee, Ama Edwin, Kerstin Klipstein‐Grobusch, Rieke van der Graaf

**Affiliations:** ^1^ Julius Global Health Julius Center for Health Sciences and Primary Care University Medical Center Utrecht Utrecht The Netherlands; ^2^ Department of Medical Humanities Julius Center for Health Sciences and Primary Care University Medical Center Utrecht Utrecht The Netherlands; ^3^ Ghana Health Service, Research and Development Division Accra Ghana; ^4^ Public Health Faculty Ghana College of Physicians and Surgeons Accra Ghana; ^5^ Department of Psychological Medicine and Mental Health School of Medicine University of Health and Allied Sciences Ho Ghana; ^6^ Division of Epidemiology & Biostatistics School of Public Health Faculty of Health Sciences University of the Witwatersrand Johannesburg South Africa

**Keywords:** health, low‐ and middle‐income countries, willingness to participate, barriers to participate, reasons for participation, reasons for non‐participation, informed consent, santé, pays à revenu faible ou intermédiaire, volonté de participer, obstacles à la participation, raisons de la participation, raisons de la non‐participation, consentement éclairé

## Abstract

**Objectives:**

To systematically review reasons for the willingness to participate in biomedical human subjects research in low‐ and middle‐income countries (LMICs).

**Methods:**

Five databases were systematically searched for articles published between 2000 and 2017 containing the domain of ‘human subjects research’ in ‘LMICs’ and determinant ‘reasons for (non)participation’. Reasons mentioned were extracted, ranked and results narratively described.

**Results:**

Ninety‐four articles were included, 44 qualitative and 50 mixed‐methods studies. Altruism, personal health benefits, access to health care, monetary benefit, knowledge, social support and trust were the most important reasons for participation. Primary reasons for non‐participation were safety concerns, inconvenience, stigmatisation, lack of social support, confidentiality concerns, physical pain, efficacy concerns and distrust. Stigmatisation was a major concern in relation to HIV research. Reasons were similar across different regions, gender, non‐patient or patient participants and real or hypothetical study designs.

**Conclusions:**

Addressing factors that affect (non‐)participation in the planning process and during the conduct of research may enhance voluntary consent to participation and reduce barriers for potential participants.

## Abstract (fr)

**Objectifs:**

Analyser systématiquement les raisons de la volonté de participer à la recherche biomédicale sur les sujets humains dans les PRFI.

**Méthodes:**

Cinq bases de données ont été systématiquement recherchées pour des articles publiés entre 2000 et 2017 contenant le domaine de la «recherche sur des sujets humains» dans les «PRFI» et les déterminantes «raisons de la (non) participation». Les raisons mentionnées ont été extraites, classées et les résultats décrits de manière narrative.

**Résultats:**

94 articles ont été inclus, 44 études qualitatives et 51 études à méthodes mixtes. L'altruisme, les avantages pour la santé personnelle, l'accès aux soins de santé, les avantages pécuniaires, les connaissances, le soutien social et la confiance étaient les principales raisons de la participation. Les principales raisons de la non‐participation étaient les problèmes de sécurité, les inconvénients, la stigmatisation, le manque de soutien social, les soucis de confidentialité, la douleur physique, les soucis d'efficacité et la méfiance. La stigmatisation était une préoccupation majeure dans le cadre de la recherche sur le VIH. Les raisons étaient similaires dans les différentes régions, le sexe, les participants patients ou non‐patients et les modèles d’étude réels ou hypothétiques.

**Conclusions:**

La prise en compte des facteurs qui affectent la (non) participation au processus de planification et au cours de la recherche peut renforcer le consentement volontaire à la participation et réduire les obstacles pour les participants potentiels.

## Introduction

Ample studies have addressed the willingness of human subjects to participate in biomedical research. Some studies focused on ethical aspects, looking into voluntary informed consent and the relation between participants’ motivations and the level of voluntariness that they display. Others looked into practical aspects in an attempt to understand barriers for research participation and improve recruitment and retention rates 1, 2, 3, 4. Studies on the willingness to participate include research in specific populations such as pregnant women or children with cancer, ethnic minorities, and in varying contexts in high as well as low‐ and middle‐income countries (LMICs) 2, 5, 6, 7, 8, 9.

Systematic reviews on willingness to participate are rare 7, 10, and do not exist for research participation in LMICs specifically. At the same time, these reviews are highly relevant for research conducted in LMICs since social determinants such as poverty, limited health care access, illiteracy and linguistic or cultural aspects may influence the willingness to participate and affect the understanding of research concepts such as randomisation, research risks and voluntariness 11, 12, 13, 14, 15. A better understanding of the motives of those who participate could improve informed consent processes, incorporating a culturally competent approach, and inform ethical guidelines for the design and conduct of health‐related human subjects research. We therefore aim to systematically review reasons for the willingness to participate in human subjects biomedical research in LMICs.

## Methods

### Eligibility criteria

Articles were eligible for inclusion in this systematic review if they related to the domain of biomedical research involving human beings in LMICs (as defined by the World Bank) and addressed ‘reasons to (not) participate’ 16. Articles were included if published after the year 2000, following amendments in the guidelines for research ethics in low‐resource settings 17. Articles were excluded if they concerned secondary analysis or were not published in English or Dutch.

### Data search

A systematic search of the electronic databases PubMed, Embase, Cochrane Library, Popline and GHL (Global Health Library) was conducted to include all articles up to June 27^th^, 2017. A search string involving relevant key words and possible variations was constructed based on the domain (human subjects research in LMIC) and determinant (reasons for (non‐) participation, see Appendix S1).

### Study selection

Studies were screened for title and abstract based on the eligibility criteria independently by two reviewers (CR and JB). Reasons for exclusion were registered. Discordance of article relevance between reviewers (CR and JB) was discussed and resolved, with full‐text articles being assessed and a third reviewer (RvdG) consulted if necessary. If the full‐text article was not available online, one attempt was made to contact the author, and if no response was received the article was excluded.

### Data collection

Data extraction of the articles was performed by two reviewers (CR and JB) for the following items: authors, year of publication, original study design, indication (disease), country, participants, study design of the nested study, aim of derived study, reasons identified and generic reasons identified. ‘Original study design’ referred to the type of research of which the willingness to participate was investigated, ‘nested study’ referred to at which point the ‘willingness to participate’ in research was investigated (hypothetical, prospective or retrospective). ‘Generic reasons’ were the groupings of individually different reasons given in relevant articles.

### Data items

The various reasons for and against participation were classified into categories as defined in Table 1. Categories were defined by the authors (JB, CR, RvdG) after data extraction, based on themes derived from previously published similar reviews 18.

**Table 1:** Categorisation and definitions of reasons

Reasons category	Generic reason	Explanation
Participation		
Personal benefit	Access to health care	Receiving free access to medical treatment in the form of ancillary care, ‘access to quality care’, ‘free medical treatment’, etc.
	Personal health benefits	A benefit associated specifically with the disease/condition being addressed in the research. For instance, ‘protection for HIV’ in an HIV vaccine trial, or ‘HIV testing and counselling’ in an HIV prevention trial
	Need for treatment	Participant would rely on research to obtain specific treatment, particularly in the case of patient participation
	Monetary benefit	Financial and/or material gain
	Knowledge (existing/expanding)	Having previous knowledge of the indication/research, or participating in research in order to ‘gain knowledge’ or ‘receive education’ about a certain disease, or alternatively to ‘satisfy curiosity’.
	Perception of being at risk	Perceiving oneself of being at high risk of contracting the disease covered by the research (e.g. HIV vaccine)
	Feeling of community	Social group forming between participants
Benefit for others	Altruism	‘Doing something good for community’, ‘ability to help others’, ‘solidarity with sufferers’, ‘helping to further research’, ‘benefit society’ and other similar sentiments
	Community involvement	The research benefits/involves specifically the community of the participant in some way
Agreeable research aspects	Guarantee of confidentiality	Being assured of adequate confidentiality with regard to participation/personal details
	Allowing withdrawal	Participants free to withdraw from study
	Convenience	Taking part does not take up much time/is accessible
	Result availability	Results made available to participants at research conclusion
	Researcher attitude	Positive attitude of researchers
	Non‐invasive procedure	Procedures done in the research are not extensively/at all invasive
Social acceptance	Cultural acceptability	Participation is considered appropriate according to local cultural/religious norms
	Trust	Trust in researchers, regulations, medicine
	Social support	Society's, family members’, and/or friends’ approval or encouragement for participation in the research
	Peer enrolment	Friends or peers have (previously) enrolled in the research
	Research outcome	Participants are supportive of the research objective, e.g. vaccine development
	Advice from physician	Following advice of health professional (doctor, nurse, health worker, etc.)
Non‐participation		
Physical harm	Safety concerns	Fear of side effects, sero‐conversion, fear of gaining a disease from vaccination, fear of physical harm, not wanting to be used as a guinea pig
	Invasive procedures	Lack of willingness to undergo invasive procedures
	Physical pain	Fear of specific procedures, repeated blood draw/vaccinations
	Worsening of medical condition	Recurrent illnesses/conditions
Social harm	Confidentiality concerns	Concerns about personal details/details of participation
	Cultural insensitivity	Aspects of research do not comply with aspects of participant's culture
	Lack of social support	Friends/Peers/Family members/Partner do not approve of participation, or discourage participation
	Stigmatisation	Social disapproval/discrimination for participation
Practical inconveniences	Inconvenience	Research site too far, participation takes up too much time, not compatible with schedule
	False‐positive test results	Receiving a false‐positive test results as a result of a vaccination (e.g. for HIV, comparable to reaction to Mantoux test after BCG vaccination)
	Non‐compliance to terms of research	Lack of willingness to comply to terms of research, e.g. child‐bearing, or cessation of current treatment
	Personal costs	Unwilling to spend money on transportation costs etc
Disagreeable research aspects	Lack of clarity	Lack of proper explanation or understanding of specific aspects of research, e.g. ‘lack of information’, ‘inadequate information’, ‘lack of understanding’
	Insufficient compensation	Compensation (material or monetary) offered for research participation deemed insufficient
	Efficacy concerns	Skepticism of efficacy of (e.g.) vaccine
	Placebo concerns	Unwilling to receive placebo
	Re‐contact	No desire of being re‐contacted
Personal opinions/assumptions	Distrust	Distrust of researchers, drug companies, governments, regulatory bodies, physicians (misconceptions)
	Previous negative experience	Previous negative experience with research/indication
	Lack of knowledge	Lack of sufficient or accurate knowledge about general research aspects, thereby not feeling at ease about participation
	Lack of interest	No interest in area of research, or research participation
	No perceived need	Satisfaction with available drugs/treatments, or denial of existence of problem, no wish for further treatment
	Overwhelmed	Other ongoing (social, emotional) issues (e.g. dealing with a dramatic diagnosis)
	Fear of health status	Fear of positive test results, health concerns
	Temptation to unsafe behaviour	Treatment gives participants a false sense of security to undertake more risky behaviour (e.g. unsafe sex after HIV vaccine)

### Data synthesis

The analysis aimed to provide an overall ranking of frequency of reasons for participation and the relative importance of reasons compared to others in two steps. First, it was assessed whether a reason was listed in an article. Subsequently, for the papers that provided ranking information with regard to relative importance of reasons, the order of the top three reasons was determined. Given the heterogeneity of methods to determine relative ranking of reasons across studies, the ranking as reported in each of the studies was used.

Thus, three rankings were created: (i) the absolute frequency a reason was listed in the articles, (ii) the frequency a reason was ranked in the top three, (iii) and how many times a reason was ranked as most important. A descriptive composite ranking of importance was subsequently created based on these three categories, in an attempt to globally suggest which reasons may be the most important.

For papers that did not provide information on the relative importance of a reason compared to others, the article was only considered for the absolute number of times a reason was mentioned.

Ranking of reasons were stratified for the following categories, if available in more than one article: World regions (as defined by the World Bank, with the regions of ‘South Asia’ and ‘East and Asia & Pacific’ combined into ‘Asia’), male *vs*. female, non‐patient *vs*. patients, HIV research *vs*. non‐HIV research (due to the hypothesis possibility that stigmatisation could influence research participation, particularly in HIV research) and hypothetical (i.e. empirical studies that ask participants about potential participation in studies that do not (yet) exist or enrol participants) *vs*. ‘real’ studies 16. ‘Real’ studies (i.e. empirical studies nested in research for which participants were recruited/enrolled in) could either be prospective or retrospective. An article was categorised into a specific region by study location. If a paper concerned multiple countries or regions, the information specific to individual regions was extracted.

### Quality assessment

Owing to the nature of the research question for this review, the risk of bias for included studies was not investigated. The protocol for this systematic review was registered in the PROSPERO database (CRD42015017126).

## Results

**Figure 1:**
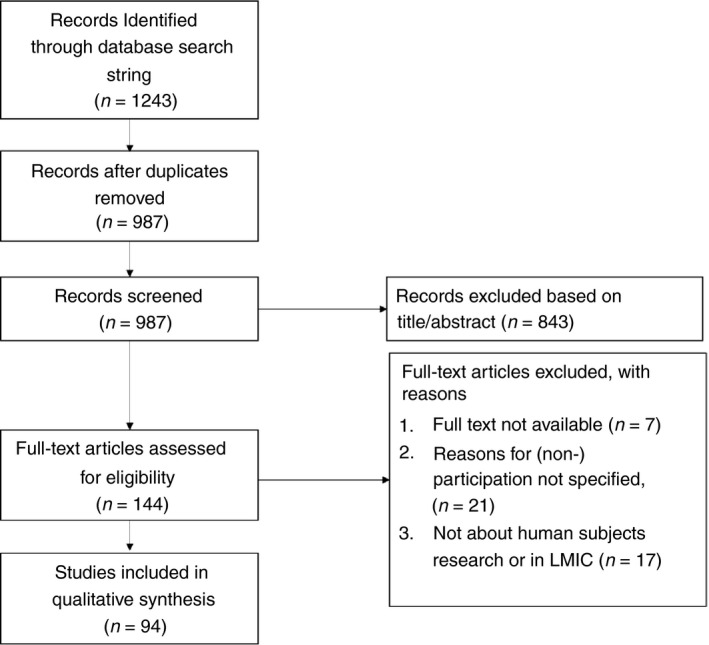
Flow diagram of review process.

The search across all databases yielded a total of 1243 results of which 987 unique articles remained after removal of duplicates. One hundred and forty‐four articles were screened in full‐text, resulting in 94 articles included in this systematic review. Figure 1 presents study selection flow diagram. Table S1 presents an overview of included articles, the characteristics of which are summarised in Table 2. The majority of articles (*n* = 54) reported both reasons for and against participation in research. Most were hypothetical (*n* = 64), and all were qualitative (*n* = 44) or mixed methods (*n* = 50). The majority of articles reported on studies about specific diseases, most commonly infectious diseases. Most studies were conducted in sub‐Saharan Africa (*n* = 45), followed by Asia (*n* = 27), Latin America and the Caribbean (*n* = 11), the Middle East and North Africa (*n* = 4) and Eastern Europe (*n* = 2).

**Table 2:** Summary of characteristics of included studies (*n* = 94)

Characteristic		*N*=, [references]
Type of reasons	Reasons for participation	21 3, 24, 25, 27, 33, 35, 38, 49, 57, 59, 60, 64, 70, 73, 74, 79, 80, 81, 82, 90, 120
	Reasons against participation	19 92, 93, 94, 95, 96, 97, 98, 99, 100, 101, 102, 103, 104, 105, 106, 107, 108, 109, 111
	Both	54 1, 19, 20, 21, 22, 23, 28, 29, 30, 31, 32, 34, 36, 37, 39, 40, 42, 43, 44, 45, 46, 47, 48, 50, 51, 52, 53, 54, 55, 56, 58, 61, 62, 63, 65, 66, 67, 68, 69, 71, 72, 75, 76, 77, 78, 84, 85, 86, 87, 88, 89, 91
Study nature	Hypothetical study	64 3, 20, 21, 22, 23, 24, 25, 28, 29, 30, 31, 32, 34, 35, 36, 37, 39, 40, 41, 42, 43, 44, 45, 46, 47, 48, 49, 50, 51, 52, 53, 54, 55, 56, 58, 59, 61, 62, 63, 65, 66, 67, 69, 70, 71, 72, 75, 77, 80, 81, 86, 87, 88, 90, 91, 93, 94, 95, 96, 97, 98, 101, 106, 111
	‘Real’/embedded study	
	Prospective	21 1, 19, 27, 38, 57, 64, 68, 73, 76, 78, 83, 85, 89, 92, 99, 100, 103, 104, 107, 109, 120
	Retrospective	9 33, 60, 74, 79, 82, 84, 102, 105, 108
Study methods	Quantitative	
	Qualitative	44 3, 19, 20, 22, 23, 24, 28, 29, 33, 34, 38, 39, 42, 43, 44, 48, 49, 57, 61, 64, 66, 71, 72, 73, 75, 79, 81, 83, 84, 86, 87, 89, 90, 95, 97, 99, 100, 108, 111, 120
	Mixed methods	50 1, 21, 25, 27, 30, 31, 32, 35, 36, 37, 45, 46, 47, 50, 51, 52, 53, 54, 55, 56, 58, 59, 60, 62, 65, 67, 68, 69, 70, 74, 76, 77, 80, 85, 88, 91, 93, 94, 96, 98, 101, 102, 103, 105, 106, 109
Types of studies reasons were assessed for	Clinical trials	5 19, 30, 74, 78, 120
	Non‐therapeutic trials	1 27
	Bio‐banks	1 91
	Dental research	1 20
	(Medical) research in general	7 1, 3, 21, 58, 61, 79, 111
	Genomics Research	1 66
Disease/disorder focus	Infectious diseases	
	HIV	40 22, 23, 25, 28, 31, 32, 33, 34, 36, 37, 38, 40, 42, 43, 44, 45, 46, 47, 48, 49, 50, 51, 52, 53, 54, 56, 57, 59, 62, 65, 67, 68, 69, 70, 71, 75, 82, 83, 85, 86, 88, 89, 92, 93, 94, 95, 98, 101, 106, 107, 108
	Malaria	6 60, 80, 83, 84, 87, 99
	Tuberculosis	2 39, 97
	Sexually transmitted infections	3 41, 53, 57
	Typhoid fever	1 76
	RSV	1 84
	Non‐infectious diseases	
	Cancer	6 29, 77, 90, 102, 104, 109
	Stroke	1 100
	Dementia	1 24
	Haemophilia	1 35
	Childhood obesity	1 103
	Pre‐eclampsia	2 63, 105
	Rheumatoid arthritis	2 55, 74
	Mental health	1 72
	Cardiovascular Disease	3 64, 73
Regions	Sub‐Saharan Africa	45 3, 19, 25, 28, 31, 33, 35, 37, 38, 39, 40, 42, 44, 49, 50, 57, 60, 62, 63, 67, 69, 70, 72, 75, 79, 80, 82, 83, 84, 85, 86, 87, 88, 89, 90, 92, 93, 94, 95, 96, 97, 99, 101, 106, 107, 108, 111, 120
	Middle East and North Africa	4 1, 20, 61, 91
	Latin America and the Caribbean	11 29, 45, 56, 64, 68, 74, 78, 93, 100, 103, 109
	Asia	27 21, 22, 23, 24, 27, 30, 32, 36, 41, 43, 46, 47, 48, 51, 52, 53, 54, 58, 59, 65, 66, 71, 76, 77, 81, 98, 102, 104, 105
	Eastern Europe	2 55, 73

Table 3 presents the frequency a reason for (non‐)participation was mentioned in studies, the number of times ranked as a reason in the top three, and the number of times ranked as top reason. Fifty‐five studies included information on the relative importance of reasons mentioned. Table 4 provides the composite summary of relative importance. Table S2 provides ranking of reason per article, Table S3 the number of times a reason was listed, and Tables S4–8 ranking by different population characteristics. Figures S1–S13 visualise the data provided here.

**Table 3:** Frequency reasons for and against participation in human subjects research were mentioned in included studies (*n* = 94)

Reasons for participation				Reasons for non‐participation			
	× Mentioned (*n* = 73)	× Top 3 (*n* = 41)	× Top Reason (*n* = 41)		× Mentioned (*n* = 71)	× Top 3 (*n* = 47)	× Top Reason (*n* = 47)
Ability to withdraw	1	1	0	Confidentiality concerns	12	4	3
Access to Health Care	42	20	4	Costs	5	3	1
Altruism	46	30	20	Cultural insensitivity	2	2	0
Advice from physician	4	2	0	Distrust	14	4	0
Community involvement	5	2		Efficacy concerns	12	10	2
Convenience	3	0	0	False‐positive test results	8	4	1
Cultural acceptability	2	1	1	Lack of social support	23	11	3
Feeling of community	1			Fear of health status	5	3	0
Personal health benefits	40	21	8	Inconvenience	25	14	4
Knowledge	16	4	1	Insufficient compensation	2		
Monetary benefit	31	10	2	Invasive procedures	8	6	4
Low pressure decision	1			Lack of interest	10	3	0
Need for treatment	1	1	1	Lack of Clarity	7	4	3
Non‐invasive procedure	2	2	1	Non‐compliance to terms of research	5	3	1
Peer enrolment	4			No perceived need	5	2	0
Low perception of risk	3	0	0	Overwhelmed	1		
Personal benefit	5	1	1	Physical pain	13	5	2
Result availability	1	1	0	Placebo concerns	6	4	1
Social support	18	6	0	Previous negative experience	2		
Trust	17	6	0	Re‐contact	1	1	0
Guarantee of Confidentiality	4	1	0	Safety concerns	45	32	16
Unaware of voluntariness	1	0	0	Stigmatisation	20	6	2
Motivation to avoid risky behaviour	1	0	0	Temptation to unsafe behaviour	1		
Research Outcomes	5	1	0	Lack of Perceived Benefit	1		
				Effect on lifestyle	2	0	0
				Worsening of Medical condition	3	2	1
				Lack of Knowledge	7	3	1

**Table 4:** Ranking of reasons

	Top reasons for participation	Top reasons for non‐participation
1	Altruism	Safety Concerns
2	Personal Health Benefits	Inconvenience
3	Access to Health Care	Lack of Social Support
4	Monetary Benefit	Stigmatisation
5	Knowledge	Confidentiality Concerns
6	Social Support	Physical Pain
7	Trust	Efficacy Concerns
8		Distrust

Reasons most frequently mentioned and indicated as relatively most important within studies in favour of participation, were altruism, personal health benefits, access to health care, monetary benefit, knowledge, social support and trust. Overall, these were common across LMICs in different regions, real and hypothetical studies, for both HIV and non‐HIV research, for men and women and for non‐patient and patient participants (Tables 3, 4; Figures S1–13).

### Altruism

Altruism was mentioned in 46 of 94 articles and thus the most often cited reason for study participation 1, 19, 20, 21, 22, 23, 24, 25, 26, 27, 28, 29, 30, 31, 32, 33, 34, 35, 36, 37, 38, 39, 40, 41, 42, 43, 44, 45, 46, 47, 48, 49, 50, 51, 52, 53, 54, 55, 56, 57, 58, 59, 60, 61, 62, 63, 64. It was ranked in the top three 30 times 1, 19, 20, 21, 23, 27, 30, 31, 32, 36, 37, 40, 41, 44, 45, 46, 47, 48, 50, 51, 52, 53, 54, 55, 56, 57, 58, 59, 61, 65, 66, and the top reason for participation 20 times 1, 19, 20, 21, 23, 30, 31, 32, 36, 37, 45, 48, 52, 54, 56, 58, 59, 61, 66. Altruism was ranked first in all regions, except for Eastern Europe, where it was ranked third, in both HIV and non‐HIV research, among non‐patient and patient participants, for hypothetical and real studies and for male participants.

### Personal health benefits

Personal health benefits were mentioned as a motivator for research participation in 40 papers 22, 25, 26, 27, 28, 30, 31, 33, 36, 39, 40, 41, 42, 43, 44, 46, 47, 48, 50, 51, 52, 53, 54, 56, 57, 58, 59, 60, 62, 63, 64, 67, 68, 69, 70, 71, 72, 73, 74, ranked in the top three in 21 papers 30, 31, 36, 40, 41, 44, 46, 47, 48, 50, 51, 52, 53, 54, 56, 57, 58, 65, 67, 68, 71, and reported as the top reason for participation nine times 40, 46, 47, 51, 53, 57, 65, 67, 68. This category ranked second for the regions of Sub‐Saharan Africa, Asia and South and Latin America, HIV research, for non‐patient participants, male participants and real and hypothetical studies, and was ranked first overall in articles involving female participants.

### Access to health care

Access to health care was mentioned as a motivator to participate in research 42 times, 3, 19, 20, 21, 24, 25, 27, 29, 31, 32, 33, 34, 38, 40, 41, 49, 50, 53, 54, 55, 57, 58, 60, 62, 63, 64, 67, 71, 72, 73, 74, 75, 76, 77, 78, 79, 80, 81, 82, 83, 84, 85 ranked in the top three 20 times 19, 21, 27, 31, 32, 40, 41, 50, 53, 54, 55, 57, 58, 67, 71, 77, 78, 80, 81, 85, and was ranked first in four studies 41, 71, 78, 80, 85. It ranked third overall in articles for Sub‐Saharan Africa, Asia, South and Latin America and in articles concerning both male and female participants and for hypothetical studies and second in Eastern Europe. It was ranked as second for non‐HIV research and real studies, fourth for HIV research, and third and second for non‐patient and patient participants, respectively.

### Monetary benefit

Monetary benefit was mentioned as a reason 31 times 3, 19, 20, 21, 22, 23, 24, 27, 31, 32, 33, 35, 37, 41, 45, 48, 49, 50, 53, 54, 55, 58, 59, 60, 69, 71, 81, 82, 83, 85, 86, ranked in the top three ten times 19, 21, 27, 37, 45, 48, 59, 69, 71, 85, and ranked as the top reason twice 27, 69. Several studies stated that monetary benefit was one of the less important influencing factors in participation 35, 38, it being ranked fourth overall in importance. This was ranked as the third most important reason in HIV and non‐HIV research, was ranked fourth for the regions of Sub‐Saharan Africa, Asia and Eastern Europe, and for patient and non‐patient participants, male and female participants and for real and hypothetical studies. It was ranked fifth for South and Latin America, and ninth for North Africa and the Middle East.

### Knowledge

The gaining of knowledge through research participation was mentioned 16 times overall, 25, 27, 41, 44, 54, 59, 60, 62, 67, 73, 76, 81, 82, 86, 87, 88 ranked in the top three reasons four times, 44, 59, 67, 88 and was given as the top reason in one paper to participate 44. Knowledge was ranked fifth in Sub‐Saharan Africa and Eastern Europe, in HIV research, by male and patient participants and for hypothetical studies. It was ranked sixth for research conducted in Asia.

### Social support

Social support as encouragement or approval to participate in research by family members, community or friends was mentioned 18 times 20, 22, 23, 32, 35, 36, 42, 53, 59, 63, 69, 75, 78, 84, 85, 88, 89, 90, in the top three reasons in six studies 20, 32, 36, 69, 78, and ranked sixth overall. Social support seemed to play a larger role in Asia than in other regions. Furthermore, it appeared to be slightly more important for HIV research than non‐HIV research (being ranked sixth and seventh in these categories, respectively). There was no difference between male and female participants’ perspective (or between real and hypothetical studies) of social support as a reason for participation, but was more important for patients than non‐patient participants. However, a few articles suggested that the influence of family and friends was more important for women (46, 78). Furthermore, social support was ranked higher in North Africa (ranked third) and the Middle East in comparison to other regions.

### Trust

Trust was mentioned in 17 articles 1, 30, 53, 54, 55, 56, 60, 62, 63, 69, 72, 73, 74, 78, 85, 87, and was ranked in the top three in six papers 1, 30, 56, 69, 78, 85. While the reason of ‘trust’ was ranked seventh overall, it was ranked higher (fourth) for research in South and Latin America and North Africa and the Middle East, as well as for non‐HIV research, patient participants and for real studies (ranked fifth).

### Other reasons for participation

Additional reasons for participation mentioned were: ability to withdraw 91, advice from physician 20, 27, 77, 85, community involvement 34, 61, 62, 63, 66, cultural acceptability 63, 91, creating a feeling of community 44, low pressure decision 75, need for treatment 77, research involving a non‐invasive procedure 46, 81, peer enrolment 33, 44, 84, low perception of risk 54, 70, result availability 20, guarantee of confidentiality 23, 33, 34, 62, being unaware of voluntariness of participation 46, research outcome, 60, 62, 66, 84 and finally seeing research participation as motivation to avoid risky behaviour 59.

The most important reasons for non‐participation were safety concerns, inconvenience, stigmatisation, lack of social support, confidentiality concerns, physical pain, efficacy concerns and distrust. Overall, these were common across different regions, real and hypothetical studies, HIV and non‐HIV research, men and women and non‐patient and patient participants (Tables 4–5, S1–13).

### Safety concerns

Safety concerns were the most often mentioned reason for non‐participation. This was a particular issue for vaccine or drug trials, but not for observational studies. Safety concerns were mentioned in 45 papers 1, 20, 21, 22, 23, 26, 28, 30, 31, 32, 34, 36, 37, 39, 40, 41, 42, 45, 47, 48, 50, 51, 52, 53, 55, 56, 58, 59, 61, 62, 67, 68, 69, 71, 76, 77, 88, 92, 93, 94, 95, 96, 97, 98, 99, ranked in the top three reasons 32 times 1, 20, 21, 23, 30, 31, 32, 36, 37, 40, 41, 45, 47, 48, 50, 51, 52, 53, 55, 56, 58, 61, 65, 67, 68, 69, 71, 77, 93, 94, 96, 97, 98, and identified as the top reason for non‐participation 16 times 31, 32, 40, 45, 47, 48, 55, 58, 61, 65, 68, 71, 77, 93, 94, 96, 98. Safety concerns were consistently ranked as most important in all categories, with the exception of North Africa and the Middle East, where they ranked second. In some articles, it was explicitly mentioned that women were generally more ‘worried about complications’ 27.

### Inconvenience

Inconvenience was mentioned in 25 articles as a reason for non‐participation 20, 28, 30, 31, 32, 34, 37, 42, 44, 46, 50, 51, 54, 58, 67, 69, 71, 100, 101, 102, 103, 104, 105, 106, ranked as the top three reasons fourteen times 31, 44, 46, 50, 54, 67, 69, 71, 100, 101, 102, 104, 106, and ranked first in four articles 67, 101, 102, 104. Examples of inconveniences included not having enough time to participate in research, transport issues or a long distance to the research site 67, 71.

### Stigmatisation

Especially in trials about HIV and other STIs, stigmatisation was named a barrier. Despite being ranked third overall, stigmatisation was consistently ranked higher for HIV research, specifically in Sub‐Saharan Africa and Asia, among non‐patients, and female participants. Stigmatisation was in fact only mentioned once in relation to non‐HIV research, suggesting that despite being mentioned 20 times overall 22, 23, 31, 32, 36, 37, 41, 42, 47, 50, 52, 53, 68, 69, 75, 86, 93, 94, 98, 107, being ranked in the top 35 times 23, 32, 41, 50, 94, and as the top reasons thrice 23, 41, 50, it is not one of the more important reasons for non‐participation when looking at human subjects research in general.

### Lack of social support

Lack of social support was mentioned as a reason for non‐participation 23 times 32, 41, 44, 48, 53, 54, 68, 72, 75, 76, 77, 84, 85, 88, 89, 93, 94, 98, 102, 105, 108, 109, 110, ranked in the top three eleven times 32, 41, 48, 50, 53, 77, 93, 94, 102, 105, 108, and given as the top reason three times 53, 105, 108. Lack of social support was found to be ranked higher (third) for research being conducted in Asia, involving HIV and women, whilst playing a smaller role in the regions of Sub‐Saharan Africa, North Africa and the Middle East and Eastern Europe. Furthermore, it seems to be slightly less important for non‐patient participants over patient participants.

### Confidentiality concerns

Confidentiality concerns were mentioned 12 times 20, 21, 22, 43, 44, 46, 58, 86, 91, 107, ranked in the top three, four times 21, 44, 46, 91, and ranked first, three times 44, 46, 91. Confidentiality concerns were ranked highest by research participants in North Africa and the Middle East (second), even though it was ranked fifth overall. This reason was not assigned the same importance for Eastern Europe or South and Latin America, or for HIV research. Furthermore, they seemed more important for male participants than female participants, and were also more important for patient participants over non‐patient participants and hypothetical over real studies.

### Physical pain

(Fear of) physical pain was mentioned as a reason 13 times 19, 20, 28, 31, 44, 47, 50, 53, 54, 55, 76, 83, 105, ranked in the top three to five times 19, 20, 31, 47, 105, and given as the most important reason twice 19, 47. It seemed to be slightly more important for male participants, patient participants, for non‐HIV research, and research participants in Sub‐Saharan Africa. For South and Latin America this reason was least cited.

### Efficacy concerns

Efficacy concerns were mentioned 12 times, 36, 40, 45, 47, 51, 52, 53, 59, 77, 92, 97, 98 ranked in the top three ten times 36, 40, 45, 47, 51, 52, 59, 77, 97, 98, and ranked first twice 59, 97. This was of the most importance for research conducted in Asia, and in HIV research. For men and studies conducted in Eastern Europe, South and Latin America and North Africa and the Middle East, it was a less important reason.

### Distrust

Distrust was mentioned in 14 articles 21, 34, 37, 39, 41, 42, 45, 53, 55, 56, 58, 62, 83, and given as a top three reason four times 41, 56, 58, 61. Distrust was an important factor mostly in Eastern Europe, and was ranked as the eighth most important reason overall.

### Other reasons for non‐participation

Additional reasons given for non‐participation were costs 44, 58, 69, 103, 111, cultural insensitivity 37, 101, fear of false‐positive test results after participation in HIV research 36, 44, 45, 50, 59, 86, 93, 94, fear of knowledge of health status 19, 28, 30, 54, 68, insufficient compensation 21, 43, invasive procedures, 1, 20, 30, 39, 54, 78, 87, 106 lack of interest 30, 34, 37, 61, 86, 95, 102, 104, 105, 109, lack of clarity 1, 30, 37, 51, 52, 91, 111, not willing to comply to terms of research 55, 68, 93, 106, 108, no perceived need 29, 53, 55, 65, 98, feeling overwhelmed 109, placebo concerns 29, 40, 52, 53, 54, 55, having had a previous negative experience 75, 111, not wishing to be re‐contacted 91, feeling tempted to unsafe behaviour 43, lack of perceived benefit 92, effect on lifestyle 20, 53, worsening medical condition, 99, 100, 102 and having a lack of knowledge about research 26, 55, 56, 75, 88, 95, 101.

## Discussion

This systematic review shows that the most important reasons for willingness to participate in research (altruism, personal health benefits and access to health care) or not (safety concerns, inconvenience, stigmatisation and lack of social support) are common across LMICs in different regions, for both HIV and non‐HIV research, for men and women and for non‐patient and patient participants. Research professionals and ethics committees addressing the interests of LMICs (study) populations can use the results from this review to prepare for and conduct research in these environments.

Some of the reasons identified in this review could influence the voluntary decision to participate in research. For example, (expected) personal or community health benefits, access to health care, (dis)trust or community pressure could affect autonomy in the consent to participate in research, or *de facto* constitute controlling influences affecting autonomy 15, 112.

As many of the reasons to (not) participate are linked to socio‐economic factors relatively common in LMIC contexts (such as poverty and illiteracy), the complete removal of these influences seems unrealistic for study investigators. Literature and international ethical guidelines for research conduct 14 mention a number of ways that could help to mitigate the potential threats of these reasons to participate to voluntary informed consent. Simultaneously, barriers to research identified in this review, such as need for/lack of social support, fear of stigmatisation, inconvenience and therapeutic misconception can also be addressed using these approaches 112, 113.

First, community engagement, in which the role of the family and community (leaders) in decision making is acknowledged and incorporated 112. Community engagement addresses the importance of (expected) personal and/or community benefit in the decision to participate in research, and can enhance the understanding of research 112. The 2016 CIOMS International Ethical Guidelines for Health‐related Research Involving Humans similarly recommend to engage communities when conducting (clinical) research in low‐resource settings to ensure ethical and scientific quality 14.

Second, the potentially inappropriate influence of reasons to (not) participate on voluntary consent could be attenuated by balancing the decision to participate in research against a person's expressed values in the consent process 15. This ‘threshold inquiry’ assesses whether the (potential) participant would also have participated in the absence of these influences and as such not persuade, coerce or manipulate a person into participation. Importantly, these influences are *potentially* ‐but not by definition inappropriate. Thus, a threshold inquiry allows for an assessment of whether the inducement for trial participation (e.g. access to health care) is sufficiently weighted against the risk the person assumes, and as such does not result in ‘poor judgement which makes us take unnecessary, unreasonable and excessive risks of harm, whether physical harm or the harm of violating important values’ – as there is nothing ‘unethical or wrong when individuals considering entering a trial weigh the inducement against the risk they will assume’ 114.

Therefore, the third manner in which potential influences could be addressed is to incorporate procedures in informed consent processes that safeguard the understanding of the nature and implications of the research. Various existing strategies can be employed, including sufficient time for subjects to consider their participation and discuss it with family and friends; and provision of adequate information about what research entails (about research in general and the specific research in particular) from someone without a dependency relationship (such as between physician and patient) (4, 15, 112).

Previous reviews of reasons for research participation have been conducted for specific LMIC populations, healthy volunteers in predominantly high‐resource settings and specific subpopulations in high‐resource settings. Reviews summarising studies conducted in Brazil, India and China, similarly identified the importance of altruism, personal benefit and access to health 2, 9. Overall, participation in human subjects research seems an effort of subjects to improve their personal or community's circumstances, and this effort generally outweighs monetary gain in importance 7, 9, 115, 116, 117. This contrasts with a review of reasons among healthy volunteers to participate in clinical trials in mainly high‐resource settings (United States (*n* studies = 6), Portugal, Spain, the Netherlands, Croatia, Germany, United Kingdom and Malawi (all *n* = 1), in which financial rewards were reported a primary motivation to participate – albeit altruistic motives informed the decision as well 118. For specific (patient) populations in high‐resource settings – children and their parents participating in drug research, women with breast cancer, cancer patients and minority populations in the United States – altruism and access to health care were (more) important considerations in the decision to participate in research 5, 6, 7, 8, 118.

The major reasons for non‐participation – concern for safety; distrust of research or health professionals; privacy concerns, and a fear of social consequences – were also reported by previous reviews in LMICs, high‐resource settings and among specific subpopulations 7, 9, 115, 119.

To the best of our knowledge, this is the first systematic review to investigate motivations that influence willingness to participate across LMICs as a whole. As we stratified our results by various study characteristics, the results can be generalised to a wide scope of human subjects research. The comprehensive inclusion of study designs, both qualitative and quantitative methods, is a strength of this study.

This review, however, has limitations that should be considered when interpreting the findings. First, the method by which the importance of reasons for participation was determined may not yield indisputable results, as a standardised methodology of ranking of reasons of (non‐) participation is not available. We aimed to provide a structured overview with a ranking of relative importance, a quantitative improvement over previously published reviews 9. Second, a majority of studies included were hypothetical (64 out of 94), and the extent to which these reflect real life situations may vary. Nonetheless, the ranking of reasons for (non‐)participation between hypothetical and ‘real’ studies yielded similar results. We did not look specifically into the difference in reasons to participate based on the method of data collection (e.g. interview *vs*. self‐administered questionnaire) or study design (e.g. observational *vs*. interventional research). It is possible that differences in reasons for (non‐) participation could be found between these groups. Furthermore, the paucity of data of studies from North Africa, the Middle East and Eastern Europe, as well as from non‐infectious disease research, limits the generalisability of the results to these domains. The same can be said about the fact that we limited relevant articles to those written in the Dutch or English language, meaning literature written in other languages common to LMIC (i.e. French, Spanish) was not taken into account in these reviews.

This review identified a number of research needs for global health (research) ethics. First, a standardised way to collect data on reasons for (non‐)participation in research and synthesis of preferences would allow for better comparison and analysis of data across studies. This would eliminate many of the limitations identified for this review. Ideally, these tools could help researchers to assess motivators and barriers to conduct of the study in the feasibility or piloting stage. Second, given potential similarities in the reasons to (not) participate between LMICs populations and disadvantaged populations in high‐income countries resulting from socio‐economic disadvantage, further research into the reasons for (non‐) participation in these groups may be of value, including systematic synthesis of the body of literature up to now. Similarly, very few reviews included (potentially) marginalised or hard‐to‐reach populations in LMICs such as (ethnic) minority groups or members of the LGBT community.

The main motivations to participate in human subject research in LMICs are altruism, a desire for personal health benefits, and access to health care. Safety concerns, inconvenience, a lack of social support and – for HIV‐related studies – stigmatisation are the major reasons for non‐participation in these populations. In order to ensure voluntary consent to participation and reduce barriers for potential participants, these reasons for (non‐) participation should be considered in the planning and conduct of research.

## Supporting information


**Appendix S1**. Search strings.
**Figure S1.** Overall ranking of reasons for participation (Graph A) and non‐participation (Graph B). Visualisation of data given in Table 2.
**Figure S2.** Ranking of reasons for participation (Graph A) and non‐participation (Graph B) – Sub‐Saharan Africa.
**Figure S3.** Ranking of reasons for participation (Graph A) and non‐participation (Graph B) – Asia.
**Figure S4.** Ranking of reasons for participation (Graph A) and non‐participation (Graph B) – Latin America & the Caribbean.
**Figure S5.** Ranking of reasons for participation (Graph A) and non‐participation (Graph B) – North Africa & the Middle East.
**Figure S6.** Ranking of reasons for participation (Graph A) and non‐participation (Graph B) – HIV research.
**Figure S7.** Ranking of reasons for participation (Graph A) and non‐participation (Graph B) – Non‐ HIV research.
**Figure S8.** Ranking of reasons for participation (Graph A) and non‐participation (Graph B) – patients.
**Figure S9.** Ranking of reasons for participation (Graph A) and non‐participation (Graph B) – non‐patients.
**Figure S10.** Ranking of reasons for participation (Graph A) and non‐participation (Graph B) – males.
**Figure S11.** Ranking of reasons for participation (Graph A) and non‐participation (Graph B) – females.
**Figure S12.** Ranking of reasons for participation (Graph A) and non‐participation (Graph B) – real studies.
**Figure S13.** Ranking of reasons for participation (Graph A) and non‐participation (Graph B) – hypothetical studies.
**Table S1.** Overview of included articles (*n* = 94).
**Table S2.** Ranking of reasons per article.
**Table S3.** Number of studies in which a reason was mentioned.
**Table S4.** Overall ranking of reasons per region.
**Table S5.** Overall ranking of reasons – HIV *vs*. Non‐HIV research.
**Table S6.** Overall ranking of reasons – Non‐patient *vs*. patient participants.
**Table S7.** Overall ranking of reasons – male *vs*. female participants.
**Table S8.** Overall ranking of reasons for real *vs*. hypothetical studies.
