# Supplementary material for: The willingness to participate in biomedical research involving human beings in low‐ and middle‐income countries: a systematic review
Source: Trop Med Int Health. 2019 Jan 8;24(3):264–79. doi: 10.1111/tmi.13195 (PMC6850431; doi:10.1111/tmi.13195)
Supplement: Supplementary file 1 — Appendix S1. Search strings. Figure S1. Overall ranking of reasons for participation (Graph A) and non‐participation (Graph B). Visualisation of data given in Table 2. Figure S2. Ranking of reasons for participation (Graph A) and non‐participation (Graph B) – Sub‐Saharan Africa. Figure S3. Ranking of reasons for participation (Graph A) and non‐participation (Graph B) – Asia. Figure S4. Ranking of reasons for participation (Graph A) and non‐participation (Graph B) – Latin America & the Caribbean. Figure S5. Ranking of reasons for participation (Graph A) and non‐participation (Graph B) – North Africa & the Middle East. Figure S6. Ranking of reasons for participation (Graph A) and non‐participation (Graph B) – HIV research. Figure S7. Ranking of reasons for participation (Graph A) and non‐participation (Graph B) – Non‐ HIV research. Figure S8. Ranking of reasons for participation (Graph A) and non‐participation (Graph B) – patients. Figure S9. Ranking of reasons for participation (Graph A) and non‐participation (Graph B) – non‐patients. Figure S10. Ranking of reasons for participation (Graph A) and non‐participation (Graph B) – males. Figure S11. Ranking of reasons for participation (Graph A) and non‐participation (Graph B) – females. Figure S12. Ranking of reasons for participation (Graph A) and non‐participation (Graph B) – real studies. Figure S13. Ranking of reasons for participation (Graph A) and non‐participation (Graph B) – hypothetical studies. Table S1. Overview of included articles (n = 94). Table S2. Ranking of reasons per article. Table S3. Number of studies in which a reason was mentioned. Table S4. Overall ranking of reasons per region. Table S5. Overall ranking of reasons – HIV vs. Non‐HIV research. Table S6. Overall ranking of reasons – Non‐patient vs. patient participants. Table S7. Overall ranking of reasons – male vs. female participants. Table S8. Overall ranking of reasons for real vs. hypothetical studies. [file TMI-24-264-s001.docx]

### Supplementary File 1. Search strings

### 1.1. PubMed Search String

Human Subject Research[Title/Abstract] OR Human Subjects Research[Title/Abstract] OR clinical trial[Title/Abstract] OR trial*[Title/Abstract] OR randomized controlled trial[Title/Abstract] OR efficacy trial[Title/Abstract] OR Biobank*[Title/Abstract] OR Questionnaire[Title/Abstract] OR Biomedical Research[Title/Abstract] OR Experimental treatment[Title/Abstract] OR Experimental Therapy[Title/Abstract] OR experimental therapy[Title/Abstract] OR prevention[Title/Abstract] OR Biorepositor*[Title/Abstract] OR “stored human biological materials”[Title/Abstract] OR “stored human samples”[Title/Abstract] OR preparedness[Title/Abstract] OR “community engagement”[Title/Abstract] OR Survey[Title/Abstract] OR “Community-based participatory research”[Title/Abstract] OR Tissue Bank[Title/Abstract] OR Observational stud*[Title/Abstract] OR Population Bank*[Title/Abstract] OR Cohort study[Title/Abstract] OR Virtual Biobank[Title/Abstract] OR Longitudinal study[Title/Abstract] OR clinical stud*[Title/Abstract] OR intervention stud*[Title/Abstract] OR descriptive stud*[Title/Abstract] OR future research[Title/Abstract] OR future trials[Title/Abstract]

**AND**

Developing Countries[Mesh:noexp] OR Africa[Mesh:noexp] OR Africa, Northern[Mesh:noexp] OR Africa South of the Sahara[Mesh:noexp] OR Africa, Central[Mesh:noexp] OR Africa, Eastern[Mesh:noexp] OR Africa, Southern[Mesh:noexp] OR Africa, Western[Mesh:noexp] OR Asia[Mesh:noexp] OR Asia, Central[Mesh:noexp] OR Asia, Southeastern[Mesh:noexp] OR Asia, Western[Mesh:noexp] OR Caribbean Region[Mesh:noexp] OR West Indies[Mesh:noexp] OR South America[Mesh:noexp] OR Latin America[Mesh:noexp] OR Central America[Mesh:noexp] OR Afghanistan[Mesh:noexp] OR Albania[Mesh:noexp] OR Algeria[Mesh:noexp] OR American Samoa[Mesh:noexp] OR Angola[Mesh:noexp] OR "Antigua and Barbuda"[Mesh:noexp] OR Argentina[Mesh:noexp] OR Armenia[Mesh:noexp] OR Azerbaijan[Mesh:noexp] OR Bahrain[Mesh:noexp] OR Bangladesh[Mesh:noexp] OR Barbados[Mesh:noexp] OR Benin[Mesh:noexp] OR Byelarus[Mesh:noexp] OR Belize[Mesh:noexp] OR Bhutan[Mesh:noexp] OR Bolivia[Mesh:noexp] OR Bosnia-Herzegovina[Mesh:noexp] OR Botswana[Mesh:noexp] OR Brazil[Mesh:noexp] OR Bulgaria[Mesh:noexp] OR Burkina Faso[Mesh:noexp] OR Burundi[Mesh:noexp] OR Cambodia[Mesh:noexp] OR Cameroon[Mesh:noexp] OR Cape Verde[Mesh:noexp] OR Central African Republic[Mesh:noexp] OR Chad[Mesh:noexp] OR Chile[Mesh:noexp] OR China[Mesh:noexp] OR Colombia[Mesh:noexp] OR Comoros[Mesh:noexp] OR Congo[Mesh:noexp] OR Costa Rica[Mesh:noexp] OR Cote d'Ivoire[Mesh:noexp] OR Croatia[Mesh:noexp] OR Cuba[Mesh:noexp] OR Cyprus[Mesh:noexp] OR Czechoslovakia[Mesh:noexp] OR Czech Republic[Mesh:noexp] OR Slovakia[Mesh:noexp] OR Djibouti[Mesh:noexp] OR "Democratic Republic of the Congo"[Mesh:noexp] OR Dominica[Mesh:noexp] OR Dominican Republic[Mesh:noexp] OR East Timor[Mesh:noexp] OR Ecuador[Mesh:noexp] OR Egypt[Mesh:noexp] OR El Salvador[Mesh:noexp] OR Eritrea[Mesh:noexp] OR Estonia[Mesh:noexp] OR Ethiopia[Mesh:noexp] OR Fiji[Mesh:noexp] OR Gabon[Mesh:noexp] OR Gambia[Mesh:noexp] OR "Georgia (Republic)"[Mesh:noexp] OR Ghana[Mesh:noexp] OR Greece[Mesh:noexp] OR Grenada[Mesh:noexp] OR Guatemala[Mesh:noexp] OR Guinea[Mesh:noexp] OR Guinea-Bissau[Mesh:noexp] OR Guam[Mesh:noexp] OR Guyana[Mesh:noexp] OR Haiti[Mesh:noexp] OR Honduras[Mesh:noexp] OR Hungary[Mesh:noexp] OR India[Mesh:noexp] OR Indonesia[Mesh:noexp] OR Iran[Mesh:noexp] OR Iraq[Mesh:noexp] OR Jamaica[Mesh:noexp] OR Jordan[Mesh:noexp] OR Kazakhstan[Mesh:noexp] OR Kenya[Mesh:noexp] OR Korea[Mesh:noexp] OR Kosovo[Mesh:noexp] OR Kyrgyzstan[Mesh:noexp] OR Laos[Mesh:noexp] OR Latvia[Mesh:noexp] OR Lebanon[Mesh:noexp] OR Lesotho[Mesh:noexp] OR Liberia[Mesh:noexp] OR Libya[Mesh:noexp] OR Lithuania[Mesh:noexp] OR Macedonia[Mesh:noexp] OR Madagascar[Mesh:noexp] OR Malaysia[Mesh:noexp] OR Malawi[Mesh:noexp] OR Mali[Mesh:noexp] OR Malta[Mesh:noexp] OR Mauritania[Mesh:noexp] OR Mauritius[Mesh:noexp] OR Mexico[Mesh:noexp] OR Micronesia[Mesh:noexp] OR Middle East[Mesh:noexp] OR Moldova[Mesh:noexp] OR Mongolia[Mesh:noexp] OR Montenegro[Mesh:noexp] OR Morocco[Mesh:noexp] OR Mozambique[Mesh:noexp] OR Myanmar[Mesh:noexp] OR Namibia[Mesh:noexp] OR Nepal[Mesh:noexp] OR Netherlands Antilles[Mesh:noexp] OR New Caledonia[Mesh:noexp] OR Nicaragua[Mesh:noexp] OR Niger[Mesh:noexp] OR Nigeria[Mesh:noexp] OR Oman[Mesh:noexp] OR Pakistan[Mesh:noexp] OR Palau[Mesh:noexp] OR Panama[Mesh:noexp] OR Papua New Guinea[Mesh:noexp] OR Paraguay[Mesh:noexp] OR Peru[Mesh:noexp] OR Philippines[Mesh:noexp] OR Poland[Mesh:noexp] OR Portugal[Mesh:noexp] OR Puerto Rico[Mesh:noexp] OR Romania[Mesh:noexp] OR Russia[Mesh:noexp] OR "Russia (Pre-1917)"[Mesh:noexp] OR Rwanda[Mesh:noexp] OR "Saint Kitts and Nevis"[Mesh:noexp] OR Saint Lucia[Mesh:noexp] OR "Saint Vincent and the Grenadines"[Mesh:noexp] OR Samoa[Mesh:noexp] OR Saudi Arabia[Mesh:noexp] OR Senegal[Mesh:noexp] OR Serbia[Mesh:noexp] OR Montenegro[Mesh:noexp] OR Seychelles[Mesh:noexp] OR Sierra Leone[Mesh:noexp] OR Slovenia[Mesh:noexp] OR Sri Lanka[Mesh:noexp] OR Somalia[Mesh:noexp] OR South Africa[Mesh:noexp] OR Sudan[Mesh:noexp] OR Suriname[Mesh:noexp] OR Swaziland[Mesh:noexp] OR Syria[Mesh:noexp] OR Tajikistan[Mesh:noexp] OR Tanzania[Mesh:noexp] OR Thailand[Mesh:noexp] OR Togo[Mesh:noexp] OR Tonga[Mesh:noexp] OR "Trinidad and Tobago"[Mesh:noexp] OR Tunisia[Mesh:noexp] OR Turkey[Mesh:noexp] OR Turkmenistan[Mesh:noexp] OR Uganda[Mesh:noexp] OR Ukraine[Mesh:noexp] OR Uruguay[Mesh:noexp] OR USSR[Mesh:noexp] OR Uzbekistan[Mesh:noexp] OR Vanuatu[Mesh:noexp] OR Venezuela[Mesh:noexp] OR Vietnam[Mesh:noexp] OR Yemen[Mesh:noexp] OR Yugoslavia[Mesh:noexp] OR Zambia[Mesh:noexp] OR Zimbabwe[Mesh:noexp] OR Macedonia[Title/Abstract] OR Madagascar[Title/Abstract] OR Malagasy Republic[Title/Abstract] OR Malaysia[Title/Abstract] OR Malaya[Title/Abstract] OR Malay[Title/Abstract] OR Sabah[Title/Abstract] OR Sarawak[Title/Abstract] OR Malawi[Title/Abstract] OR Nyasaland[Title/Abstract] OR Mali[Title/Abstract] OR Malta[Title/Abstract] OR Marshall Islands[Title/Abstract] OR Mauritania[Title/Abstract] OR Mauritius[Title/Abstract] OR Agalega Islands[Title/Abstract] OR Mexico[Title/Abstract] OR Micronesia[Title/Abstract] OR Middle East[Title/Abstract] OR Moldova[Title/Abstract] OR Moldovia[Title/Abstract] OR Moldovian[Title/Abstract] OR Mongolia[Title/Abstract] OR Montenegro[Title/Abstract] OR Morocco[Title/Abstract] OR Ifni[Title/Abstract] OR Mozambique[Title/Abstract] OR Myanmar[Title/Abstract] OR Myanma[Title/Abstract] OR Burma[Title/Abstract] OR Namibia[Title/Abstract] OR Nepal[Title/Abstract] OR Netherlands Antilles[Title/Abstract] OR New Caledonia[Title/Abstract] OR Nicaragua[Title/Abstract] OR Niger[Title/Abstract] OR Nigeria[Title/Abstract] OR Northern Mariana Islands[Title/Abstract] OR Oman[Title/Abstract] OR Muscat[Title/Abstract] OR Pakistan[Title/Abstract] OR Palau[Title/Abstract] OR Palestine[Title/Abstract] OR Panama[Title/Abstract] OR Paraguay[Title/Abstract] OR Peru[Title/Abstract] OR Philippines[Title/Abstract] OR Philipines[Title/Abstract] OR Phillipines[Title/Abstract] OR Phillippines[Title/Abstract] OR Poland[Title/Abstract] OR Portugal[Title/Abstract] OR Puerto Rico[Title/Abstract] OR Romania[Title/Abstract] OR Rumania[Title/Abstract] OR Roumania[Title/Abstract] OR Russia[Title/Abstract] OR Russian[Title/Abstract] OR Rwanda[Title/Abstract] OR Ruanda[Title/Abstract] OR Saint Kitts[Title/Abstract] OR St Kitts[Title/Abstract] OR Nevis[Title/Abstract] OR Saint Lucia[Title/Abstract] OR St Lucia[Title/Abstract] OR Saint Vincent[Title/Abstract] OR St Vincent[Title/Abstract] OR Grenadines[Title/Abstract] OR Samoa[Title/Abstract] OR Samoan Islands[Title/Abstract] OR Navigator Island[Title/Abstract] OR Navigator Islands[Title/Abstract] OR Sao Tome[Title/Abstract] OR Saudi Arabia[Title/Abstract] OR Senegal[Title/Abstract] OR Serbia[Title/Abstract] OR Montenegro[Title/Abstract] OR Seychelles[Title/Abstract] OR Sierra Leone[Title/Abstract] OR Slovenia[Title/Abstract] OR Sri Lanka[Title/Abstract] OR Ceylon[Title/Abstract] OR Solomon Islands[Title/Abstract] OR Somalia[Title/Abstract] OR Sudan[Title/Abstract] OR Suriname[Title/Abstract] OR Surinam[Title/Abstract] OR Swaziland[Title/Abstract] OR Syria[Title/Abstract] OR Tajikistan[Title/Abstract] OR Tadzhikistan[Title/Abstract] OR Tadjikistan[Title/Abstract] OR Tadzhik[Title/Abstract] OR Tanzania[Title/Abstract] OR Thailand[Title/Abstract] OR Togo[Title/Abstract] OR Togolese Republic[Title/Abstract] OR Tonga[Title/Abstract] OR Trinidad[Title/Abstract] OR Tobago[Title/Abstract] OR Tunisia[Title/Abstract] OR Turkey[Title/Abstract] OR Turkmenistan[Title/Abstract] OR Turkmen[Title/Abstract] OR Uganda[Title/Abstract] OR Ukraine[Title/Abstract] OR Uruguay[Title/Abstract] OR USSR[Title/Abstract] OR Soviet Union[Title/Abstract] OR Union of Soviet Socialist Republics[Title/Abstract] OR Uzbekistan[Title/Abstract] OR Uzbek[Title/Abstract] OR Vanuatu[Title/Abstract] OR New Hebrides[Title/Abstract] OR Venezuela[Title/Abstract] OR Vietnam[Title/Abstract] OR Viet Nam[Title/Abstract] OR West Bank[Title/Abstract] OR Yemen[Title/Abstract] OR Yugoslavia[Title/Abstract] OR Zambia[Title/Abstract] OR Zimbabwe[Title/Abstract] OR Rhodesia[Title/Abstract] OR Africa[Title/Abstract] OR Asia[Title/Abstract] OR Caribbean[Title/Abstract] OR West Indies[Title/Abstract] OR South America[Title/Abstract] OR Latin America[Title/Abstract] OR Central America[Title/Abstract] OR Afghanistan[Title/Abstract] OR Albania[Title/Abstract] OR Algeria[Title/Abstract] OR Angola[Title/Abstract] OR Antigua[Title/Abstract] OR Barbuda[Title/Abstract] OR Argentina[Title/Abstract] OR Armenia[Title/Abstract] OR Armenian[Title/Abstract] OR Aruba[Title/Abstract] OR Azerbaijan[Title/Abstract] OR Bahrain[Title/Abstract] OR Bangladesh[Title/Abstract] OR Barbados[Title/Abstract] OR Benin[Title/Abstract] OR Byelarus[Title/Abstract] OR Byelorussian[Title/Abstract] OR Belarus[Title/Abstract] OR Belorussian[Title/Abstract] OR Belorussia[Title/Abstract] OR Belize[Title/Abstract] OR Bhutan[Title/Abstract] OR Bolivia[Title/Abstract] OR Bosnia[Title/Abstract] OR Herzegovina[Title/Abstract] OR Hercegovina[Title/Abstract] OR Botswana[Title/Abstract] OR Brasil[Title/Abstract] OR Brazil[Title/Abstract] OR Bulgaria[Title/Abstract] OR Burkina Faso[Title/Abstract] OR Burkina Fasso[Title/Abstract] OR Upper Volta[Title/Abstract] OR Burundi[Title/Abstract] OR Urundi[Title/Abstract] OR Cambodia[Title/Abstract] OR Khmer Republic[Title/Abstract] OR Kampuchea[Title/Abstract] OR Cameroon[Title/Abstract] OR Cameroons[Title/Abstract] OR Cameron[Title/Abstract] OR Camerons[Title/Abstract] OR Cape Verde[Title/Abstract] OR Central African Republic[Title/Abstract] OR Chad[Title/Abstract] OR Chile[Title/Abstract] OR China[Title/Abstract] OR Colombia[Title/Abstract] OR Comoros[Title/Abstract] OR Comoro Islands[Title/Abstract] OR Comores[Title/Abstract] OR Mayotte[Title/Abstract] OR Congo[Title/Abstract] OR Zaire[Title/Abstract] OR Costa Rica[Title/Abstract] OR Cote d'Ivoire[Title/Abstract] OR Ivory Coast[Title/Abstract] OR Croatia[Title/Abstract] OR Cuba[Title/Abstract] OR Cyprus[Title/Abstract] OR Czechoslovakia[Title/Abstract] OR Czech Republic[Title/Abstract] OR Slovakia[Title/Abstract] OR Slovak Republic[Title/Abstract] OR Djibouti[Title/Abstract] OR French Somaliland[Title/Abstract] OR Dominica[Title/Abstract] OR Dominican Republic[Title/Abstract] OR East Timor[Title/Abstract] OR East Timur[Title/Abstract] OR Timor Leste[Title/Abstract] OR Ecuador[Title/Abstract] OR Egypt[Title/Abstract] OR United Arab Republic[Title/Abstract] OR El Salvador[Title/Abstract] OR Eritrea[Title/Abstract] OR Estonia[Title/Abstract] OR Ethiopia[Title/Abstract] OR Fiji[Title/Abstract] OR Gabon[Title/Abstract] OR Gabonese Republic[Title/Abstract] OR Gambia[Title/Abstract] OR Gaza[Title/Abstract] OR Georgia Republic[Title/Abstract] OR Georgian Republic[Title/Abstract] OR Ghana[Title/Abstract] OR Gold Coast[Title/Abstract] OR Greece[Title/Abstract] OR Grenada[Title/Abstract] OR Guatemala[Title/Abstract] OR Guinea[Title/Abstract] OR Guam[Title/Abstract] OR Guiana[Title/Abstract] OR Guyana[Title/Abstract] OR Haiti[Title/Abstract] OR Honduras[Title/Abstract] OR Hungary[Title/Abstract] OR India[Title/Abstract] OR Maldives[Title/Abstract] OR Indonesia[Title/Abstract] OR Iran[Title/Abstract] OR Iraq[Title/Abstract] OR Isle of Man[Title/Abstract] OR Jamaica[Title/Abstract] OR Jordan[Title/Abstract] OR Kazakhstan[Title/Abstract] OR Kazakh[Title/Abstract] OR Kenya[Title/Abstract] OR Kiribati[Title/Abstract] OR Korea[Title/Abstract] OR Kosovo[Title/Abstract] OR Kyrgyzstan[Title/Abstract] OR Kirghizia[Title/Abstract] OR Kyrgyz Republic[Title/Abstract] OR Kirghiz[Title/Abstract] OR Kirgizstan[Title/Abstract] OR "Lao PDR"[Title/Abstract] OR Laos[Title/Abstract] OR Latvia[Title/Abstract] OR Lebanon[Title/Abstract] OR Lesotho[Title/Abstract] OR Basutoland[Title/Abstract] OR Liberia[Title/Abstract] OR Libya[Title/Abstract] OR Lithuania[Title/Abstract] OR "developing country"[Title/Abstract] OR "developing countries"[Title/Abstract] OR "developing nation"[Title/Abstract] OR "developing nations"[Title/Abstract] OR "developing population"[Title/Abstract] OR "developing populations"[Title/Abstract] OR "developing world"[Title/Abstract] OR "less developed country"[Title/Abstract] OR "less developed countries"[Title/Abstract] OR "less developed nation"[Title/Abstract] OR "less developed nations"[Title/Abstract] OR "less developed population"[Title/Abstract] OR "less developed populations"[Title/Abstract] OR "less developed world"[Title/Abstract] OR "lesser developed country"[Title/Abstract] OR "lesser developed countries"[Title/Abstract] OR "lesser developed nation"[Title/Abstract] OR "lesser developed nations"[Title/Abstract] OR "lesser developed population"[Title/Abstract] OR "lesser developed populations"[Title/Abstract] OR "lesser developed world"[Title/Abstract] OR "under developed country"[Title/Abstract] OR "under developed countries"[Title/Abstract] OR "under developed nation"[Title/Abstract] OR "under developed nations"[Title/Abstract] OR "under developed population"[Title/Abstract] OR "under developed populations"[Title/Abstract] OR "under developed world"[Title/Abstract] OR "underdeveloped country"[Title/Abstract] OR "underdeveloped countries"[Title/Abstract] OR "underdeveloped nation"[Title/Abstract] OR "underdeveloped nations"[Title/Abstract] OR "underdeveloped population"[Title/Abstract] OR "underdeveloped populations"[Title/Abstract] OR "underdeveloped world"[Title/Abstract] OR "middle income country"[Title/Abstract] OR "middle income countries"[Title/Abstract] OR "middle income nation"[Title/Abstract] OR "middle income nations"[Title/Abstract] OR "middle income population"[Title/Abstract] OR "middle income populations"[Title/Abstract] OR "low income country"[Title/Abstract] OR "low income countries"[Title/Abstract] OR "low income nation"[Title/Abstract] OR "low income nations"[Title/Abstract] OR "low income population"[Title/Abstract] OR "low income populations"[Title/Abstract] OR "lower income country"[Title/Abstract] OR "lower income countries"[Title/Abstract] OR "lower income nation"[Title/Abstract] OR "lower income nations"[Title/Abstract] OR "lower income population"[Title/Abstract] OR "lower income populations"[Title/Abstract] OR "underserved country"[Title/Abstract] OR "underserved countries"[Title/Abstract] OR "underserved nation"[Title/Abstract] OR "underserved nations"[Title/Abstract] OR "underserved population"[Title/Abstract] OR "underserved populations"[Title/Abstract] OR "underserved world"[Title/Abstract] OR "under served country"[Title/Abstract] OR "under served countries"[Title/Abstract] OR "under served nation"[Title/Abstract] OR "under served nations"[Title/Abstract] OR "under served population"[Title/Abstract] OR "under served populations"[Title/Abstract] OR "under served world"[Title/Abstract] OR "deprived country"[Title/Abstract] OR "deprived countries"[Title/Abstract] OR "deprived nation"[Title/Abstract] OR "deprived nations"[Title/Abstract] OR "deprived population"[Title/Abstract] OR "deprived populations"[Title/Abstract] OR "deprived world"[Title/Abstract] OR "poor country"[Title/Abstract] OR "poor countries"[Title/Abstract] OR "poor nation"[Title/Abstract] OR "poor nations"[Title/Abstract] OR "poor population"[Title/Abstract] OR "poor populations"[Title/Abstract] OR "poor world"[Title/Abstract] OR "poorer country"[Title/Abstract] OR "poorer countries"[Title/Abstract] OR "poorer nation"[Title/Abstract] OR "poorer nations"[Title/Abstract] OR "poorer population"[Title/Abstract] OR "poorer populations"[Title/Abstract] OR "poorer world"[Title/Abstract] OR "developing economy"[Title/Abstract] OR "developing economies"[Title/Abstract] OR "less developed economy"[Title/Abstract] OR "less developed economies"[Title/Abstract] OR "lesser developed economy"[Title/Abstract] OR "lesser developed economies"[Title/Abstract] OR "under developed economy"[Title/Abstract] OR "under developed economies"[Title/Abstract] OR "underdeveloped economy"[Title/Abstract] OR "underdeveloped economies"[Title/Abstract] OR "middle income economy"[Title/Abstract] OR "middle income economies"[Title/Abstract] OR "low income economy"[Title/Abstract] OR "low income economies"[Title/Abstract] OR "lower income economy"[Title/Abstract] OR "lower income economies"[Title/Abstract] OR "low gdp"[Title/Abstract] OR "low gnp"[Title/Abstract] OR "low gross domestic"[Title/Abstract] OR "low gross national"[Title/Abstract] OR "lower gdp"[Title/Abstract] OR "lower gnp"[Title/Abstract] OR "lower gross domestic"[Title/Abstract] OR "lower gross national"[Title/Abstract] OR lmic[Title/Abstract] OR lmics[Title/Abstract] OR "third world"[Title/Abstract] OR "lami country"[Title/Abstract] OR "lami countries"[Title/Abstract] OR "transitional country"[Title/Abstract] OR "transitional countries"[Title/Abstract] OR "developing countries"[MeSH Term] OR "developing country"[MeSH Term] OR "poverty"[MeSH Term] OR low resource[Title/Abstract] OR limited resource[Title/Abstract] OR constrained resource[Title/Abstract] OR restricted resource[Title/Abstract] OR delimited resource[Title/Abstract] OR restrained resource[Title/Abstract] OR emerging country[Title/Abstract] OR emerging countries[Title/Abstract]

**AND**

“Willingness to participate”[Title/Abstract] OR “Motivation to participate”[Title/Abstract] OR “attitudes towards research”[Title/Abstract] OR “motivate to consent”[Title/Abstract] OR “motivating factor to participate” [Title/Abstract] OR willing to consent[Title/Abstract] OR “participation and retention”[Title/Abstract] OR “Reasons to participate”[Title/Abstract] OR “community engagement”[Title/Abstract] OR “Facilitators to participate”[Title/Abstract] OR Trial preparedness[Title/Abstract] OR “Interest in participation”[Title/Abstract] OR “Agree to participate”[Title/Abstract] OR “Refusal to participate”[Title/Abstract] OR “Barriers to participation”[Title/Abstract] OR “barriers to participate” [Title/Abstract] OR “unwilling to participate”[Title/Abstract] OR “challenges to participation”[Title/Abstract] OR “concerns regarding participation”[Title/Abstract] OR trial participation[Title/Abstract] OR research participation[Title/Abstract]

### 1.2. Embase Search String

‘Human Subject Research’:ab,ti OR ‘Human Subjects Research’:ab,ti OR ‘clinical trial’:ab,ti OR trial:ab,ti OR ‘randomized controlled trial’:ab,ti OR ‘efficacy trial’:ab,ti OR Biobank*:ab,ti OR Questionnaire:ab,ti OR ‘Biomedical Research’:ab,ti OR ‘Experimental treatment’:ab,ti OR ‘Experimental Therapy’:ab,ti OR prevention:ab,ti OR Biorepositor*:ab,ti OR ‘stored human biological materials’:ab,ti OR ‘stored human samples’:ab,ti OR preparedness:ab,ti OR ‘community engagement’:ab,ti OR Survey:ab,ti OR ‘Community-based participatory research’:ab,ti OR ‘Tissue Bank’:ab,ti OR ‘Observational study’:ab,ti OR ‘Population Bank’:ab,ti OR ‘Cohort study’:ab,ti OR Virtual Biobank:ab,ti OR Longitudinal study:ab,ti OR clinical stud*:ab,ti OR ‘intervention study’:ab,ti OR ‘descriptive study’:ab,ti OR ‘future research’:ab,ti OR ‘future trials’:ab,ti

AND

('Developing Country'/exp) OR (Africa or Asia or Caribbean or West Indies or South America or Latin America or Central America):ab,ti or Afghanistan:ab,ti OR Albania:ab,ti OR Algeria:ab,ti OR Angola:ab,ti OR Antigua:ab,ti OR Barbuda:ab,ti OR Argentina:ab,ti OR Armenia:ab,ti OR Armenian:ab,ti OR Aruba:ab,ti OR Azerbaijan:ab,ti OR Bahrain:ab,ti OR Bangladesh:ab,ti OR Barbados:ab,ti OR Benin:ab,ti OR Byelarus:ab,ti OR Byelorussian:ab,ti OR Belarus:ab,ti OR Belorussian:ab,ti OR Belorussia:ab,ti OR Belize:ab,ti OR Bhutan:ab,ti OR Bolivia:ab,ti OR Bosnia:ab,ti OR Herzegovina:ab,ti OR Hercegovina:ab,ti OR Botswana:ab,ti OR Brasil:ab,ti OR Brazil:ab,ti OR Bulgaria:ab,ti OR ‘Burkina Faso’:ab,ti OR ‘Burkina Fasso’:ab,ti OR ‘Upper Volta’:ab,ti OR Burundi:ab,ti OR Urundi:ab,ti OR Cambodia:ab,ti OR ‘Khmer Republic’:ab,ti OR Kampuchea:ab,ti OR Cameroon:ab,ti OR Cameroons:ab,ti OR Cameron:ab,ti OR Camerons:ab,ti OR ‘Cape Verde’:ab,ti OR ‘Central African Republic’:ab,ti OR Chad:ab,ti OR Chile:ab,ti OR China:ab,ti OR Colombia:ab,ti OR Comoros:ab,ti OR ‘Comoro Islands’:ab,ti OR Comores:ab,ti OR Mayotte:ab,ti OR Congo:ab,ti OR Zaire:ab,ti OR ‘Costa Rica’:ab,ti OR ‘Cote d Ivoire’:ab,ti or ‘Ivory Coast’:ab,ti or Croatia:ab,ti or Cuba:ab,ti or Cyprus:ab,ti or Czechoslovakia:ab,ti or ‘Czech Republic’:ab,ti or Slovakia:ab,ti or ‘Slovak Republic’:ab,ti or Djibouti:ab,ti or ‘French Somaliland’:ab,ti or Dominica:ab,ti or ‘Dominican Republic’:ab,ti or ‘East Timor’:ab,ti or ‘East Timur’:ab,ti or ‘Timor Leste’:ab,ti or Ecuador:ab,ti or Egypt:ab,ti or ‘United Arab Republic’:ab,ti or ‘El Salvador’:ab,ti or Eritrea:ab,ti or Estonia:ab,ti or Ethiopia:ab,ti or Fiji:ab,ti or Gabon:ab,ti or ‘Gabonese Republic’:ab,ti or Gambia:ab,ti or Gaza:ab,ti or ‘Georgia Republic’:ab,ti or ‘Georgian Republic’:ab,ti or Ghana:ab,ti or ‘Gold Coast’:ab,ti or Greece:ab,ti or Grenada:ab,ti or Guatemala:ab,ti or Guinea:ab,ti or Guam:ab,ti or Guiana:ab,ti or Guyana:ab,ti or Haiti:ab,ti or Honduras:ab,ti or Hungary:ab,ti or India:ab,ti or Maldives:ab,ti or Indonesia:ab,ti or Iran:ab,ti or Iraq:ab,ti or ‘Isle of Man’:ab,ti or Jamaica:ab,ti or Jordan:ab,ti or Kazakhstan:ab,ti or Kazakh:ab,ti or Kenya:ab,ti or Kiribati:ab,ti or Korea:ab,ti or Kosovo:ab,ti or Kyrgyzstan:ab,ti or Kirghizia:ab,ti or ‘Kyrgyz Republic’:ab,ti or Kirghiz:ab,ti or Kirgizstan:ab,ti or ‘Lao PDR’:ab,ti or Laos:ab,ti or Latvia:ab,ti or Lebanon:ab,ti or Lesotho:ab,ti or Basutoland:ab,ti or Liberia:ab,ti or Libya:ab,ti or Lithuania:ab,ti or Macedonia:ab,ti or Madagascar:ab,ti or ‘Malagasy Republic’:ab,ti or Malaysia:ab,ti or Malaya:ab,ti or Malay:ab,ti or Sabah:ab,ti or Sarawak:ab,ti or Malawi:ab,ti or Nyasaland:ab,ti or Mali:ab,ti or Malta:ab,ti or ‘Marshall Islands’:ab,ti or Mauritania:ab,ti or Mauritius:ab,ti or ‘Agalega Islands’:ab,ti or Mexico:ab,ti or Micronesia:ab,ti or ‘Middle East’:ab,ti or Moldova:ab,ti or Moldovia:ab,ti or Moldovian:ab,ti or Mongolia:ab,ti or Montenegro:ab,ti or Morocco:ab,ti or Ifni:ab,ti or Mozambique:ab,ti or Myanmar:ab,ti or Myanma:ab,ti or Burma:ab,ti or Namibia:ab,ti or Nepal:ab,ti or ‘Netherlands Antilles’:ab,ti or ‘New Caledonia’:ab,ti or Nicaragua:ab,ti or Niger:ab,ti or Nigeria:ab,ti or ‘Northern Mariana Islands’:ab,ti or Oman:ab,ti or Muscat:ab,ti or Pakistan:ab,ti or Palau:ab,ti or Palestine:ab,ti or Panama:ab,ti or Paraguay:ab,ti or Peru:ab,ti or Philippines:ab,ti or Philipines:ab,ti or Phillipines:ab,ti or Phillippines:ab,ti or Poland:ab,ti or Portugal:ab,ti or ‘Puerto Rico’:ab,ti or Romania:ab,ti or Rumania:ab,ti or Roumania:ab,ti or Russia:ab,ti or Russian:ab,ti or Rwanda:ab,ti or Ruanda:ab,ti or ‘Saint Kitts’:ab,ti or ‘St Kitts’:ab,ti or Nevis:ab,ti or ‘Saint Lucia’:ab,ti or ‘St Lucia’:ab,ti or ‘Saint Vincent’:ab,ti or ‘St Vincent’:ab,ti or Grenadines:ab,ti or Samoa:ab,ti or ‘Samoan Islands’:ab,ti or ‘Navigator Island’:ab,ti or ‘Navigator Islands’:ab,ti or ‘Sao Tome’:ab,ti or ‘Saudi Arabia’:ab,ti or Senegal:ab,ti or Serbia:ab,ti or Montenegro:ab,ti or Seychelles:ab,ti or ‘Sierra Leone’:ab,ti or Slovenia:ab,ti or ‘Sri Lanka’:ab,ti or Ceylon:ab,ti or ‘Solomon Islands’:ab,ti or Somalia:ab,ti or ‘South Africa’:ab,ti or Sudan:ab,ti or Suriname:ab,ti or Surinam:ab,ti or Swaziland:ab,ti or Syria:ab,ti or Tajikistan:ab,ti or Tadzhikistan:ab,ti or Tadjikistan:ab,ti or Tadzhik:ab,ti or Tanzania:ab,ti or Thailand:ab,ti or Togo:ab,ti or ‘Togolese Republic’:ab,ti or Tonga:ab,ti or Trinidad:ab,ti or Tobago:ab,ti or Tunisia:ab,ti or Turkey:ab,ti or Turkmenistan:ab,ti or Turkmen:ab,ti or Uganda:ab,ti or Ukraine:ab,ti or Uruguay:ab,ti or USSR:ab,ti or 'Soviet Union':ab,ti OR ‘Union of Soviet Socialist Republics’:ab,ti OR Uzbekistan:ab,ti OR Uzbek:ab,ti OR Vanuatu:ab,ti OR ‘New Hebrides’:ab,ti OR Venezuela:ab,ti OR Vietnam:ab,ti OR ‘Viet Nam’:ab,ti OR ‘West Bank’:ab,ti OR Yemen:ab,ti OR Yugoslavia:ab,ti OR Zambia:ab,ti OR Zimbabwe:ab,ti OR Rhodesia:ab,ti OR Afghanistan/exp OR Albania/exp OR Algeria/exp OR Angola/exp OR Antigua/exp OR Barbuda/exp OR Argentina/exp OR Armenia/exp OR Armenian/exp OR Aruba/exp OR Azerbaijan/exp OR Bahrain/exp OR Bangladesh/exp OR Barbados/exp OR Benin/exp OR Byelarus/exp OR Byelorussian/exp OR Belarus/exp OR Belorussian/exp OR Belorussia/exp OR Belize/exp OR Bhutan/exp OR Bolivia/exp OR Bosnia/exp OR Herzegovina/exp OR Hercegovina/exp OR Botswana/exp OR Brasil/exp OR Brazil/exp OR Bulgaria/exp OR ‘Burkina Faso’/exp OR ‘Burkina Fasso’/exp OR ‘Upper Volta’/exp OR Burundi/exp OR Urundi/exp OR Cambodia/exp OR ‘Khmer Republic’/exp OR Kampuchea/exp OR Cameroon/exp OR Cameroons/exp OR Cameron/exp OR Camerons/exp OR ‘Cape Verde’/exp OR ‘Central African Republic’/exp OR Chad/exp OR Chile/exp OR China/exp OR Colombia/exp OR Comoros/exp OR ‘Comoro Islands’/exp OR Comores/exp OR Mayotte/exp OR Congo/exp OR Zaire/exp OR ‘Costa Rica’/exp OR ‘Cote d Ivoire’/exp or ‘Ivory Coast’/exp or Croatia/exp or Cuba/exp or Cyprus/exp or Czechoslovakia/exp or ‘Czech Republic’/exp or Slovakia/exp or ‘Slovak Republic’/exp or Djibouti/exp or ‘French Somaliland’/exp or Dominica/exp or ‘Dominican Republic’/exp or ‘East Timor’/exp or ‘East Timur’/exp or ‘Timor Leste’/exp or Ecuador/exp or Egypt/exp or ‘United Arab Republic’/exp or ‘El Salvador’/exp or Eritrea/exp or Estonia/exp or Ethiopia/exp or Fiji/exp or Gabon/exp or ‘Gabonese Republic’/exp or Gambia/exp or Gaza/exp or ‘Georgia Republic’/exp or ‘Georgian Republic’/exp or Ghana/exp or ‘Gold Coast’/exp or Greece/exp or Grenada/exp or Guatemala/exp or Guinea/exp or Guam/exp or Guiana/exp or Guyana/exp or Haiti/exp or Honduras/exp or Hungary/exp or India/exp or Maldives/exp or Indonesia/exp or Iran/exp or Iraq/exp or ‘Isle of Man’/exp or Jamaica/exp or Jordan/exp or Kazakhstan/exp or Kazakh/exp or Kenya/exp or Kiribati/exp or Korea/exp or Kosovo/exp or Kyrgyzstan/exp or Kirghizia/exp or ‘Kyrgyz Republic’/exp or Kirghiz/exp or Kirgizstan/exp or ‘Lao PDR’/exp or Laos/exp or Latvia/exp or Lebanon/exp or Lesotho/exp or Basutoland/exp or Liberia/exp or Libya/exp or Lithuania/exp or Macedonia/exp or Madagascar/exp or ‘Malagasy Republic’/exp or Malaysia/exp or Malaya/exp or Malay/exp or Sabah/exp or Sarawak/exp or Malawi/exp or Nyasaland/exp or Mali/exp or Malta/exp or ‘Marshall Islands’/exp or Mauritania/exp or Mauritius/exp or ‘Agalega Islands’/exp or Mexico/exp or Micronesia/exp or ‘Middle East’/exp or Moldova/exp or Moldovia/exp or Moldovian/exp or Mongolia/exp or Montenegro/exp or Morocco/exp or Ifni/exp or Mozambique/exp or Myanmar/exp or Myanma/exp or Burma/exp or Namibia/exp or Nepal/exp or ‘Netherlands Antilles’/exp or ‘New Caledonia’/exp or Nicaragua/exp or Niger/exp or Nigeria/exp or ‘Northern Mariana Islands’/exp or Oman/exp or Muscat/exp or Pakistan/exp or Palau/exp or Palestine/exp or Panama/exp or Paraguay/exp or Peru/exp or Philippines/exp or Philipines/exp or Phillipines/exp or Phillippines/exp or Poland/exp or Portugal/exp or ‘Puerto Rico’/exp or Romania/exp or Rumania/exp or Roumania/exp or Russia/exp or Russian/exp or Rwanda/exp or Ruanda/exp or ‘Saint Kitts’/exp or ‘St Kitts’/exp or Nevis/exp or ‘Saint Lucia’/exp or ‘St Lucia’/exp or ‘Saint Vincent’/exp or ‘St Vincent’/exp or Grenadines/exp or Samoa/exp or ‘Samoan Islands’/exp or ‘Navigator Island’/exp or ‘Navigator Islands’/exp or ‘Sao Tome’/exp or ‘Saudi Arabia’/exp or Senegal/exp or Serbia/exp or Montenegro/exp or Seychelles/exp or ‘Sierra Leone’/exp or Slovenia/exp or ‘Sri Lanka’/exp or Ceylon/exp or ‘Solomon Islands’/exp or Somalia/exp or ‘South Africa’/exp or Sudan/exp or Suriname/exp or Surinam/exp or Swaziland/exp or Syria/exp or Tajikistan/exp or Tadzhikistan/exp or Tadjikistan/exp or Tadzhik/exp or Tanzania/exp or Thailand/exp or Togo/exp or ‘Togolese Republic’/exp or Tonga/exp or Trinidad/exp or Tobago/exp or Tunisia/exp or Turkey/exp or Turkmenistan/exp or Turkmen/exp or Uganda/exp or Ukraine/exp or Uruguay/exp or USSR/exp or 'Soviet Union'/exp OR ‘Union of Soviet Socialist Republics’/exp OR Uzbekistan/exp OR Uzbek/exp OR Vanuatu/exp OR ‘New Hebrides’/exp OR Venezuela/exp OR Vietnam/exp OR ‘Viet Nam’/exp OR ‘West Bank’/exp OR Yemen/exp OR Yugoslavia/exp OR Zambia/exp OR Zimbabwe/exp OR Rhodesia/exp OR ‘developing country’:ab,ti OR ‘developing countries’:ab,ti OR ‘developing nation’:ab,ti OR ‘developing nations’:ab,ti OR ‘developing population’:ab,ti OR ‘developing populations’:ab,ti OR ‘developing world’:ab,ti OR ‘less developed country’:ab,ti OR ‘less developed countries’:ab,ti OR ‘less developed nation’:ab,ti OR ‘less developed nations’:ab,ti OR ‘less developed population’:ab,ti OR ‘less developed populations’:ab,ti OR ‘less developed world’:ab,ti OR ‘lesser developed country’:ab,ti OR ‘lesser developed countries’:ab,ti OR ‘lesser developed nation’:ab,ti OR ‘lesser developed nations’:ab,ti OR ‘lesser developed population’:ab,ti OR ‘lesser developed populations’:ab,ti OR ‘lesser developed world’:ab,ti OR ‘under developed country’:ab,ti OR ‘under developed countries’:ab,ti OR ‘under developed nation’:ab,ti OR ‘under developed nations’:ab,ti OR ‘under developed population’:ab,ti OR ‘under developed populations’:ab,ti OR ‘under developed world’:ab,ti OR ‘underdeveloped country’:ab,ti OR ‘underdeveloped countries’:ab,ti OR ‘underdeveloped nation’:ab,ti OR ‘underdeveloped nations’:ab,ti OR ‘underdeveloped population’:ab,ti OR ‘underdeveloped populations’:ab,ti OR ‘underdeveloped world’:ab,ti OR ‘middle income country’:ab,ti OR ‘middle income countries’:ab,ti OR ‘middle income nation’:ab,ti OR ‘middle income nations’:ab,ti OR ‘middle income population’:ab,ti OR ‘middle income populations’:ab,ti OR ‘low income country’:ab,ti OR ‘low income countries’:ab,ti OR ‘low income nation’:ab,ti OR ‘low income nations’:ab,ti OR ‘low income population’:ab,ti OR ‘low income populations’:ab,ti OR ‘lower income country’:ab,ti OR ‘lower income countries’:ab,ti OR ‘lower income nation’:ab,ti OR ‘lower income nations’:ab,ti OR ‘lower income population’:ab,ti OR ‘lower income populations’:ab,ti OR ‘underserved country’:ab,ti OR ‘underserved countries’:ab,ti OR ‘underserved nation’:ab,ti OR ‘underserved nations’:ab,ti OR ‘underserved population’:ab,ti OR ‘underserved populations’:ab,ti OR ‘underserved world’:ab,ti OR ‘under served country’:ab,ti OR ‘under served countries’:ab,ti OR ‘under served nation’:ab,ti OR ‘under served nations’:ab,ti OR ‘under served population’:ab,ti OR ‘under served populations’:ab,ti OR ‘under served world’:ab,ti OR ‘deprived country’:ab,ti OR ‘deprived countries’:ab,ti OR ‘deprived nation’:ab,ti OR ‘deprived nations’:ab,ti OR ‘deprived population’:ab,ti OR ‘deprived populations’:ab,ti OR ‘deprived world’:ab,ti OR ‘poor country’:ab,ti OR ‘poor countries’:ab,ti OR ‘poor nation’:ab,ti OR ‘poor nations’:ab,ti OR ‘poor population’:ab,ti OR ‘poor populations’:ab,ti OR ‘poor world’:ab,ti OR ‘poorer country’:ab,ti OR ‘poorer countries’:ab,ti OR ‘poorer nation’:ab,ti OR ‘poorer nations’:ab,ti OR ‘poorer population’:ab,ti OR ‘poorer populations’:ab,ti OR ‘poorer world’:ab,ti OR ‘developing economy’:ab,ti OR ‘developing economies’:ab,ti OR ‘less developed economy’:ab,ti OR ‘less developed economies’:ab,ti OR ‘lesser developed economy’:ab,ti OR ‘lesser developed economies’:ab,ti OR ‘under developed economy’:ab,ti OR ‘under developed economies’:ab,ti OR ‘underdeveloped economy’:ab,ti OR ‘underdeveloped economies’:ab,ti OR ‘middle income economy’:ab,ti OR ‘middle income economies’:ab,ti OR ‘low income economy’:ab,ti OR ‘low income economies’:ab,ti OR ‘lower income economy’:ab,ti OR ‘lower income economies’:ab,ti OR ‘low gdp’:ab,ti OR ‘low gnp’:ab,ti OR ‘low gross domestic’:ab,ti OR ‘low gross national’:ab,ti OR ‘lower gdp’:ab,ti OR ‘lower gnp’:ab,ti OR ‘lower gross domestic’:ab,ti OR ‘lower gross national’:ab,ti OR lmic:ab,ti OR lmics:ab,ti OR ‘third world’:ab,ti OR ‘lami country’:ab,ti OR ‘lami countries’:ab,ti OR ‘transitional country’:ab,ti OR ‘transitional countries’:ab,ti OR ‘developing country’/exp OR ‘poverty’/exp OR ((low:ab,ti OR limited:ab,ti OR constrained:ab,ti OR restricted:ab,ti OR delimited:ab,ti OR restrained:ab,ti) AND (resource:ab,ti)) OR 'emerging country':ab,ti OR 'emerging countries':ab,ti

AND

‘Willingness to participate’:ab,ti OR ‘Motivation to participate’:ab,ti OR ‘attitudes towards research’:ab,ti OR ‘motivate to consent’:ab,ti OR ‘motivating factor to participate’:ab,ti OR ‘willing to consent’:ab,ti OR ‘participation and retention’:ab,ti OR ‘Reasons to participate’:ab,ti OR ‘community engagement’:ab,ti OR ‘Facilitators to participate’:ab,ti OR ‘Trial preparedness’:ab,ti OR ‘Interest in participation’:ab,ti OR ‘Agree to participate’:ab,ti OR ‘Refusal to participate’:ab,ti OR ‘Barriers to participation’:ab,ti OR ‘barriers to participate’:ab,ti OR ‘unwilling to participate’:ab,ti OR ‘challenges to participation’:ab,ti OR ‘concerns regarding participation’:ab,ti OR ‘trial participation’:ab,ti OR ‘research participation’:ab,ti

### 1.3. Cochrane Search String

(Human Subject Research or Human Subjects Research or clinical trial or trial* OR randomized controlled trial OR efficacy trial OR Biobank* OR Questionnaire OR Biomedical Research OR Experimental treatment OR Experimental Therapy OR experimental therapy OR prevention OR Biorepositor* OR stored human biological materials OR stored human samples OR preparedness OR community engagement OR Survey* OR Community-based participatory research OR Tissue Bank OR Observational stud* OR Population Bank* OR Cohort study OR Virtual Biobank OR Longitudinal study OR clinical stud* OR intervention stud* OR descriptive stud* OR future research OR future trials):ti,ab,kw

AND

(Africa or Asia or Caribbean or "West Indies" or "South America" or "Latin America" or "Central America"):ti,ab,kw or (Afghanistan or Albania or Algeria or Angola or Antigua or Barbuda or Argentina or Armenia or Armenian or Aruba or Azerbaijan or Bahrain or Bangladesh or Barbados or Benin or Byelarus or Byelorussian or Belarus or Belorussian or Belorussia or Belize or Bhutan or Bolivia or Bosnia or Herzegovina or Hercegovina or Botswana or Brasil or Brazil or Bulgaria or "Burkina Faso" or "Burkina Fasso" or "Upper Volta" or Burundi or Urundi or Cambodia or "Khmer Republic" or Kampuchea or Cameroon or Cameroons or Cameron or Camerons or "Cape Verde" or "Central African Republic" or Chad or Chile or China or Colombia or Comoros or "Comoro Islands" or Comores or Mayotte or Congo or Zaire or "Costa Rica" or "Cote d'Ivoire" or "Ivory Coast" or Croatia or Cuba or Cyprus or Czechoslovakia or "Czech Republic" or Slovakia or "Slovak Republic" **or** Djibouti or "French Somaliland" or Dominica or "Dominican Republic" or "East Timor" or "East Timur" or "Timor Leste" or Ecuador or Egypt or "United Arab Republic" or "El Salvador" or Eritrea or Estonia or Ethiopia or Fiji or Gabon or "Gabonese Republic" or Gambia or Gaza or Georgia or Georgian or Ghana or "Gold Coast" or Greece or Grenada or Guatemala or Guinea or Guam or Guiana or Guyana or Haiti or Honduras or Hungary or India or Maldives or Indonesia or Iran or Iraq or "Isle of Man" or Jamaica or Jordan or Kazakhstan or Kazakh or Kenya or Kiribati or Korea or Kosovo or Kyrgyzstan or Kirghizia or "Kyrgyz Republic" or Kirghiz or Kirgizstan or "Lao PDR" or Laos or Latvia or Lebanon or Lesotho or Basutoland or Liberia or Libya or Lithuania **or** Macedonia or Madagascar or "Malagasy Republic" or Malaysia or Malaya or Malay or Sabah or Sarawak or Malawi or Nyasaland or Mali or Malta or "Marshall Islands" or Mauritania or Mauritius or "Agalega Islands" or Mexico or Micronesia or "Middle East" or Moldova or Moldovia or Moldovian or Mongolia or Montenegro or Morocco or Ifni or Mozambique or Myanmar or Myanma or Burma or Namibia or Nepal or "Netherlands Antilles" or "New Caledonia" or Nicaragua or Niger or Nigeria or "Northern Mariana Islands" or Oman or Muscat or Pakistan or Palau or Palestine or Panama or Paraguay or Peru or Philippines or Philipines or Phillipines or Phillippines or Poland or Portugal or "Puerto Rico" **or** Romania or Rumania or Roumania or Russia or Russian or Rwanda or Ruanda or "Saint Kitts" or "St Kitts" or Nevis or "Saint Lucia" or "St Lucia" or "Saint Vincent" or "St Vincent" or Grenadines or Samoa or "Samoan Islands" or "Navigator Island" or "Navigator Islands" or "Sao Tome" or "Saudi Arabia" or Senegal or Serbia or Montenegro or Seychelles or "Sierra Leone" or Slovenia or "Sri Lanka" or Ceylon or "Solomon Islands" or Somalia or Sudan or Suriname or Surinam or Swaziland or Syria or Tajikistan or Tadzhikistan or Tadjikistan or Tadzhik or Tanzania or Thailand or Togo or "Togolese Republic" or Tonga or Trinidad or Tobago or Tunisia or Turkey or Turkmenistan or Turkmen or Uganda or Ukraine or Uruguay or USSR or "Soviet Union" or "Union of Soviet Socialist Republics" or Uzbekistan or Uzbek or Vanuatu or "New Hebrides" or Venezuela or Vietnam or "Viet Nam" or "West Bank" or Yemen or Yugoslavia or Zambia or Zimbabwe or Rhodesia):ti,ab,kw or (developing or less* NEXT developed or "under developed" or underdeveloped or "middle income" or low* NEXT income or underserved or "under served" or deprived or poor*) NEXT (countr* or nation* or population* or world):ti,ab,kw or (developing or less* NEXT developed or "under developed" or underdeveloped or "middle income" or low* NEXT income) NEXT (economy or economies) **or** low* NEXT (gdp or gnp or "gross domestic" or "gross national") **or** (low NEAR/3 middle NEAR/3 countr*):ti,ab,kw **or** (lmic or lmics or "third world" or "lami country" or "lami countries"):ti,ab,kw **or** ("transitional country" or "transitional countries"):ti,ab,kw **or** (“low resource” or “ limited resource” or “constrained resource” or “restricted resource” or “delimited resource” or “restrained resource” or “emerging country” or “emerging countries”):ti,ab,kw

AND

(“Willingness to participate” OR “Motivation to participate” OR “attitudes towards research” OR “motivate to consent” OR “motivating factor to participate” OR willing to consent OR “participation and retention” OR “Reasons to participate” OR “community engagement” OR “Facilitators to participate” OR Trial preparedness OR “Interest in participation” OR “Agree to participate” OR “Refusal to participate” OR “Barriers to participation” OR “barriers to participate” OR “unwilling to participate” OR “challenges to participation” OR “concerns regarding participation” OR trial participation OR research participation):ti,ab,kw

### 1.4. GHL and Popline Search String

(Human Subject Research OR Human Subjects Research OR clinical trial OR trial* OR randomized controlled trial OR efficacy trial OR Biobank* OR Questionnaire OR Biomedical Research OR Experimental treatment OR Experimental Therapy OR experimental therapy OR prevention OR Biorepositor* OR “stored human biological materials” OR “stored human samples” OR preparedness OR “community engagement” OR Survey OR “Community-based participatory research” OR Tissue Bank OR Observational stud* OR Population Bank* OR Cohort study OR Virtual Biobank OR Longitudinal study OR clinical stud* OR intervention stud* OR descriptive stud* OR future research OR future trials)

AND

(“developing country" OR "developing countries" OR "low income country" OR "low income countries" OR "middle income country" OR "middle income countries" OR "low resource")

AND

(“Willingness to participate” OR “Motivation to participate” OR “attitudes towards research” OR “motivate to consent” OR “motivating factor to participate” OR willing to consent OR “participation and retention” OR “Reasons to participate” OR “community engagement” OR “Facilitators to participate” OR Trial preparedness OR “Interest in participation” OR “Agree to participate” OR “Refusal to participate” OR “Barriers to participation” OR “barriers to participate” OR “unwilling to participate” OR “challenges to participation” OR “concerns regarding participation” OR trial participation OR research participation)

Supplementary Table 1. Overview of included articles (n=94)

| Article, Ref # | Original study | | Indication (Disease) | | | Country | | | Participants | Derived Study Type | Derived Study Method | Aim of Derived Study | Reasons identified (% importance) | Generic Reasons Identified | |  |
| --- | --- | --- | --- | --- | --- | --- | --- | --- | --- | --- | --- | --- | --- | --- | --- | --- |
| 1.Abrams et al., 2011 | Clinical trials | | n/a | | | South Africa | | | Adolescents | Prospective | Semi-structured interviews, Qualitative | Assess experiences with research | P: Interesting (12%), Blood screening (24%), Blood donation (24%), Money (35%), Fight against TB (6%), Absence from school (12%), | P: Altruism (47%),  Access to Health care (35%), Monetary benefit (35%) | |  |
|  |  |  |  |  |  |  |  |  |  |  |  |  | NP: Fear of blood drawing/needles (53%), Fear of results (18%) | NP:  Physical pain (53%),  Fear of health status (18%) | |  |
| 2. Ahram et al., 2014 | Bio-banking | | n/a | | | Jordan | | | National population (n=3196) | Hypothetical | Survey, Cross-sectional, Mixed-methods | Assess factors influencing participation in bio-banking | P: Religious permission (47%), Availability of results (39%), Allowing withdrawal (31%) | P: Cultural acceptance (47%), Result availability (39%), Ability withdrawal (31%) | |  |
|  |  |  |  |  |  |  |  |  |  |  |  |  | NP: Access to medical history (9.5%), unspecified research (31%),  re-contact (8.5%) | NP: Confidentiality concerns (9.5%), Lack of clarity (31%), Re-contact (8.5%) | |  |
| 3. Akazili et al, 2016 | Clinical trial | | Malaria | | | Ghana | | | Parents (n=380) | Retrospective | Questionnaires, Interviews,Mixed-methods | Assess reasons for WTP | P: Better quality of care, access to care for community, disease prevention in community, improved living standards. | P: Access to Health Care, Personal Health Benefits, Knowledge, Monetary Benefit, Altruism, Trust, Research Outcome | |  |
| 4.  Al-Amad et al., 2014` | Dental research | | n/a | | | Jordan | | | Dental patients (n= 120) | Hypothetical | Survey, cross-sectional, Qualitative | Assess attitude toward research | P: Financial incentive (10%), Encourage by family (45%), Request by doctor (90%), To get treatment (30%), Advance science (95%), Help others (95%) | P: Altruism (95%), Physician’s advice (90%), Social support (45%), Access to Health care (30%), Monetary benefit (10%) | |  |
|  |  |  |  |  |  |  |  |  |  |  |  |  | NP: Invasive procedures (79%), Fear of infection (M: 53.8%, F:82.3%), pain (M:43,6%, F;73.4%), Multiple visits (M: 41%, F:58.1%), Dental Phobia (M: 28.2%, F: 64.5%), Impairment to appearance (M: 38.5%, F; 53.2%), Fear of poison (44.4%), Chewing problems (44.4%), Privacy violation (M: 35.9%, F: 20.6%) | NP: Invasive procedures (79%), Safety concerns (71%), Physical pain (62.1%), Inconvenience (51.5%), Lifestyle effect (44.4%), Confidentiality concerns (26.5%) | |  |
| 5.  Anjos et al. | Clinical trial | | Stroke rehabilitation | | | Brazil | | | Stroke patients (n=571) | Prospective | Phone interviews, Qualitative | Barriers to participation | NP: Recurrent stroke (45%), Uncontrolled medical condition (17.2%), not reachable (7%), could not comply with schedule (6.6%), | NP: Worsening of medical condition (45%), Inconvenience (6.6%) | |  |
| 6.  Asiki et al., 2013 | Vaccine trial | | HIV | | | Uganda | | | High risk individuals from fishing communities (n=328) | Hypothetical | Interview, Cohort, Mixed- Methods | Assess WTP in future HIV vaccine trials in high-risk populations | P: Access to HIV counseling and testing (31%), HIV education (18%), HIV prevention (16.6%), Health Care (12.5%). First to participate in vaccine trial (14.3%), Altruism (6.7%) | P: Personal Health Benefits (31%), Knowledge (18%) Access to Health Care (12.5%), | |  |
|  |  |  |  |  |  |  |  |  |  |  |  |  | NP: Time commitment (1.2%), Fear of vaccine safety (<1%), Transport challenges (<1%) | NP: Inconvenience (1.2%), Safety concerns (<1%) | |  |
| 7.  Bakari et al., 2013 | Vaccine trial | | HIV | | | Tanzania | | | Police officers (n= 364) | Retrospective | Interviews, Cohort, Qualitative | Assess retention/recruitment into vaccine trial | NP: Family/partner advice (7.9%), Desire to have children in 2 years (8.5%), Declined to repeat test (2.2%), | NP: Lack of social support (7.9%), Non-compliance to terms of research (11%) | |  |
| 8.  Boniphace et al., 2010 | PMTCT, prevention | | HIV | | | Tanzania | | | Males (n=138) | Hypothetical | Surveys, Cross-sectional, Mixed methods | Assess WTP | NP: Too busy (25.2%), cultural reasons (21.4%, lack of knowledge (21.4%) | NP: Inconvenience (25.2%), Cultural insensitivity (21.4%), Lack of Knowledge (21.4%) | |  |
| 9. Bouida et al., 2016 | N/a | | N/a | | | Tunisia | | | General outpatient clinic patients (n=612), healthy volunteers (n=354) | Cross-sectional, hypothetical | Questionnaires, Qualitative | WTP, reasons for WTP | P: Altruism (33%), contribution to health improvement (33%), help patients (5%). | P: Altruism (66%), Community involvement (5%). | |  |
|  |  |  |  |  |  |  |  |  |  |  |  |  | NP: risk of harmful effects (66%), distrust (21%), researches are useless (6%). | NP: Safety Concerns (66%) Distrust (21%), Lack of Interest (6%) | |  |
| 10.  Burt et al., 2013 | Biomedical Research | | n/a | | | India | | | Indian people on street (n=175) | Hypothetical | Surveys, cross-sectional, Mixed-methods | Assess public perception/WTP in clinical research | P: Benefits society (94.1%), Financial benefits, Health Care | P: Altruism (94.1%), Access to Health care, Monetary Benefit | |  |
|  |  |  |  |  |  |  |  |  |  |  |  |  | NP: Distrust of regulations (43.3%), lack of adequate compensation (75.2%), Safety, confidentiality (45.9%), Treated like guinea pigs (45%) | NP: Distrust (43.3%), Confidentiality concerns (45.9%), Insufficient compensation (75.2%), Safety concerns (45.9%) | |  |
| 11.  Chakrapani et al., 2012 | Vaccine trial | | HIV | | | India | | | Indian MSM (n=68) | Hypothetical | Focus groups & key informant Interviews, Qualitative | Assess WTP | P: Community/Government endorsement, Financial benefit, Altruism, HIV prevention | P: Social Support, Monetary benefit, Altruism, Personal Health Benefit | |  |
|  |  |  |  |  |  |  |  |  |  |  |  |  | NP: Stigmatization by community/family/partners, Disclosure of sexuality, safety concerns | NP: Stigmatization, Confidentiality concerns, Safety concerns | |  |
| 12. Chalamilla, 2012 | Vaccine trial | | HIV | | | Tanzania | | | High-risk youths (n= 1299) | Prospective | Surveys, cohort, Qualitative | Assess W/Feasibility of HIV vaccine amongst high-risk population | NP: Efficacy concerns, Safety concerns, Lack of perceived benefit | NP: Efficacy concerns, Safety concerns, Lack of perceived benefit | |  |
| 13.  Chu et al., 2013 | Vaccine trial | | HIV | | | China | | | Chinese MSM (n= 626) | Hypothetical | Self-interviewing survey, Cross-sectional, Qualitative | Assess WTP and HIV-risk behavior | P: Confidentiality guarantee (90%), Help society (89.4%), Protecting family members (93.5%), family support (75.6%), Financial gain (71%), | P: Altruism (93.5%), Confidentiality guarantee (90%), Social support (75.6%), Monetary benefit (71%) | |  |
|  |  |  |  |  |  |  |  |  |  |  |  |  | NP: Fear of discrimination (30%), Vaccine related health problems (11.6%) | NP: Stigmatization (30%), Safety concerns (11.6%) | |  |
| 14. Copenhaver et al., 2009 | Observational Cohort study | | Dementia | | | China | | | Elderly Chinese dementia patients/proxies(n=100) | Hypothetical | Interview, Case-control, Qualitative | Assess stigma of dementia, WTP in cohort study | P: Altruism, Monetary incentive, Health evaluation | P: Altruism, Monetary benefit, Access to Health care | |  |
| 15.  de Bruyn et al., 2008 | Vaccine trial | | HIV | | | South Africa | | | High school students (n= 240) | Hypothetical | Self-administered, facilitated questionnaire, Mixed-methods | Assess attitude/knowledge about HIV vaccine | P: Receive up-to-date knowledge (88.9%), 'doing something for people w/ HIV',(70.9%) free counselling/testing (70.5%), Protection from HIV (70.2%), Motivation to engage in less-risky behavior (59%) | P: Knowledge (88.9%), Altruism (70.9%), Access to Health care (70.5%), Personal Health Benefit (70.2%) | |  |
| 16. Deschamps et al., 2014 | Vaccine trial | | HIV | | | Dominican Republic, Haiti, Puerto Rico | | | Female sex workers (n= 799) | Prospective | Interviews, Cohort, Mixed-methods | Assess eligibility criteria/recruitment strategies | P: Personal health benefit (86%) | P: Personal health benefit (86%) | |  |
|  |  |  |  |  |  |  |  |  |  |  |  |  | NP: Death or Injury (70%), testing HIV positive (48%), Side effects (42%), Discrimination (25%), Negative reactions from family/friends (22%), Avoid pregnancy (10%) | NP: Safety concerns (70%), Fear of health status (48%), Stigmatization (25%), Lack of social support (22%), Non-compliance to terms of research (10%) | |  |
| 17. Dietrich et al., 2011 | Vaccine trial | | HIV | | | South Africa | | | HIV- adults (n= 30) | Hypothetical | Interviews, Cross-sectional, Qualitative | Assess WTP in future trials | P: Having enough time to decide, treatment for side-effects, family support, | P: Low pressure decision, Access to Health care, Social support | |  |
|  |  |  |  |  |  |  |  |  |  |  |  |  | NP: Being perceived as HIV+, Misconception about research intentions/trials, family disapproval, Knowing people who had died of HIV/AIDS | NP: Stigmatization, Distrust, Lack of social support, Previous negative experience | |  |
| 18. Djomand et al., 2008 | Vaccine trial | | HIV | | | Botswana (B), Dominican republic (DR), Haiti (H), Jamaica (J), Puerto Rico (PR), Peru (P) | | | HIV high-risk participants | Hypothetical | Question set, Cohort, Mixed- methods | Assess WTP in future trials | NP: Injury/Death (B:90%, DR:92%, H:80%, J:96%, P:97%), Side effects B:86%, DR:89%, H:61%, J:93%, P:90%), Partner not wanting to use condom (B:96%, DR:78%, H:74.6%, J:86%, P:87%), Antibody positivity (B:52%, DR:81%, H:40%, J:89%, P:88%), Job discrimination (B:50%, DR:70%, H:41%, J:70%, P:82%), Partner not wanting sex (B:55%, DR:70%, H:30%, J:72%, P:70%), Negative reactions from family/friends (B:52%, DR:70%, H:25%, J:60%, P:71%), Multiple HIV tests (B:15%, DR:53%, H:20%, J:65%, P:65%), Not able to donate/sell blood (B:22%, DR:46%, H:21%, J:58%, P:58%), Avoiding pregnancy (B:22%, DR:24%, H:25%, J: 47%) | NP: Safety concerns (B:90%, DR:92%, H:80%, J:96%, P:97%), Lack of social support (B:96%, DR:78%, H:74.6%, J:86%, P:87%) , False-positive testing (B:52%, DR:81%, H:40%, J:89%, P:88%),, Stigmatization B:52%, DR:70%, H:25%, J:60%, P:71%), Non-compliance to terms of research (B:22%, DR:24%, H:25%, J: 47%) | |  |
| 19. Dong et al., 2014 | Vaccine trial | | HIV | | | China | | | HIV+ ART patients | Hypothetical | Surveys, Cross-sectional, Mixed-methods | Assess WTP for future trials | P: Delay/Reduce ART treatment (76.6), Delay disease progression (74.9%), increase immune response (57.7%), prevent drug resistance (37.1%) | P: Personal Health Benefits (76.6%), Altruism (37.1%) | |  |
|  |  |  |  |  |  |  |  |  |  |  |  |  | NP: Safety concerns (37%), Lack of knowledge (33.3%), satisfaction with ART (22.2%) | NP: Safety concerns (37%), Lack of Knowledge (33.3%), No perceived need (22.2%) | |  |
| 20. Doshi et al., 2013 | Non-therapeutic trial | | n/a | | | India | | | Volunteers (healthy vs. patient  s) at tertiary referral center (n=100) | Prospective | Surveys, Cross-sectional, Mixed-methods | Assess factors motivating WTP | P: Healthy participants: Financial reward (65%), contribution to scientific progress (39%), Curiosity (14%), free medical check-up (31%), personal health benefit (13%).  Patient participants: Physician recommendation (14%), financial reward (4%), | P: Monetary benefit (65%), Altruism (39%), Access to Health care (31%), Knowledge (14%), Personal health Benefit (13%), Advice from physician (14%) | |  |
| 21. Doshi et al, 2017. | Vaccine trial | | HIV | | | Kenya | | | Healthy MSM (n=70), | Hypothetical | Historical research, ethography, interviews, surveys, Mixed-Methods | WTP, reasons for WTP | P: advantages for community (most important) including national and world at large, individual benefits including health protection, gaining of access to information, being involved at the forefront of research, altruism, trust in researchers to honour responsibilities, confidentiality, privacy/transparency/disclosure. | P: Community Involvement, Access to Health Care, Personal Health Benefits, Knowledge, Altruism, Guarantee of Confidentiality, Trust, Research Outcome. | |  |
|  |  |  |  |  |  |  |  |  |  |  |  |  | NP: side effects, not want to participate as 'guinea pigs' | NP: Safety Concerns, Distrust | |  |
| 22. Evangeli et al., 2012 | Vaccine trial | | HIV | | | South Africa | | | HIV- university students (n=238) | Hypothetical | Questionnaires, Cross-sectional, Qualitative | Effect of message framing on WTP | P: Counseling, More free time, Helping the community, Reduce HIV risk, HIV testing, | P: Personal health benefits, Altruism | |  |
|  |  |  |  |  |  |  |  |  |  |  |  |  | NP: Side effects, Fear of health status, Painful procedures, Time | NP: Safety concerns, Physical pain, Fear of health status, Inconvenience | |  |
| 23.  Farquhar et al., 2006 | Pediatric HIV trial | | HIV | | | Kenya | | | Pregnant women (n=805) | Hypothetical | Interviews, Cross-sectional, Mixed-method | Assess trial acceptability | NP: Side-effect concerns (75%), partner objection (34%), Discrimination (10%), HIV-acquisition (8%), False-positive HIV test results (5%) | NP: Safety concerns (75%), Lack of social support (34%) Stigmatization (10%), False positive test-results (5%) | |  |
| 24.  Fede et al., 2010 | Placebo-controlled trials (PCT) | | Cancer | | | Brazil | | | Cancer Patients (n=100) | Hypothetical | Questionnaires, Cross-sectional, Qualitative | Assess perceptions of PCT | P: To obtain treatment, to help medicine | P: Access to Health care, Altruism | |  |
|  |  |  |  |  |  |  |  |  |  |  |  |  | NP: No wish for treatment, no wish for placebo, | NP: No perceived need, Placebo concerns | |  |
| 25.  Fincham et al., 2010 | Vaccine trial | | HIV | | | South Africa | | | Black females (n=264) | Hypothetical | Surveys, Cross-sectional, Mixed-method | Identify facilitators/inhibitors for WTP | P: personal gains (9%), convenience, social approval (5.6%), trust (5.6%), | P: Monetary benefit (9%), Social support (5.6%), Trust (5.6%), Personal health benefit (9%) | |  |
|  |  |  |  |  |  |  |  |  |  |  |  |  | NP: personal costs (11.5%), safety (9.95%), stigmatization (9.86%), time delays (9.95%) | NP: Personal costs (11.5%), Safety concerns (9.95%), Inconvenience (9.95%)Stigmatization (9.86%), | |  |
| 26. Gitanjali et al., 2003 | Clinical trial | | n/a | | | India | | | Patients (n= 102) | Hypothetical | Mock informed consent forms, Cross-sectional, Mixed-methods | Assess consent trends | P: Help medical community (45%), Pain relief (34%), ‘doctors only do good’ (18%) | P: Altruism (45%), Personal Health benefit (34%), Trust (18%) | |  |
|  |  |  |  |  |  |  |  |  |  |  |  |  | NP: Giving blood (20%), trying a new drug (19.4%), not able to make independent decision (18.5%), afraid of tests (14.8%), ill (13.8%), do not want to interrupt treatment (6.5%), do not want to join without doctor’s permission (6.5%), not interested (5.5%), did not understand (2%), wanted to go home (2%), too old (2%) | NP: Invasive procedures (20%), Safety concerns (19.4%), Fear of health status (14.8%), Inconvenience (6.5%), Lack of Interest (5.5%), Lack of Understanding (2%), | |  |
| 27. González-Saldivar et al, 2016. | Chronic diseases | | Various phase II and II trials | | | Mexico | | | Patients with type 2 diabetes, hypertension, COPD or rheumatoid arthritis (n=604), controls (n=604), | Cross sectional | Questionnaires, interviews, Mixed-Methods | WTP, reasons for WTP | P: everything is free in clinical trials (52% cases, 54.4% controls). Unaware of risks/ do not think of risks | P: access to health care, personal health benefits, low perception of risk, trust | |  |
| 28. Groth et al, 2016. | N/a | | Genomics research | | | India | | | Company employees (n=505) | Cross sectional, hypothetical | Surveys, Qualitative | WTP, reasons for WTP | P: help children or future families (strongest), could help to find new treatment (2nd), would help to understand inherited diseases (3rd). | P: Community involvement, Research Outcomes. | |  |
|  |  |  |  |  |  |  |  |  |  |  |  |  | NP: concerns about family's history not being protected. | NP: Confidentiality Concerns | |  |
| 29. den Hollander et al, 2016. | Pregnancy trial | | Hypertensive disorders of pregnancy | | | Ghana | | | Pregnant women (n=13) | Cross sectional, hypothetical | Participant observations, semi-structured interviews, focus group discussions, Qualitative | Reasons for WTP | P: access to care, religious beliefs, recommendation from health professional, fear of repercussions, social support/experiences of others, benefit of others in community. NP: side effects, | P: Personal Health Benefits, Access to Health Care, Cultural Acceptability, Trust, Social Support, Peer Enrolment, Community Involvement, Altruism | |  |
| 30.  Idika et al., 2010 | Vaccine trial | | HIV | | | Nigeria | | | Youths (n=170) | Hypothetical | Questionnaires, Cohort, Qualitative | Assess WTP, HIV/microbicide awareness/knowledge | NP: Lack of knowledge, Fear of side effects, Lack of interest | NP: Lack of Knowledge, Safety concerns, Lack of interest | |  |
| 31.  Jaspan et al., 2006 | Vaccine trial | | HIV | | | South Africa | | | Adolescents (n=356) | Hypothetical | Survey, Cross-sectional, Mixed-methods | Assess HIV prevalence, sexual risk behavior, WTP in vaccine | P: Help find a vaccine and protect others (33%), Protection from HIV (33%), Free medical care (16%), AIDS cure (12%), Monetary benefit (11%) | P: Altruism (45%), Personal Health Benefit (45%), Access to Health care (16%), Monetary benefit (11%) | |  |
|  |  |  |  |  |  |  |  |  |  |  |  |  | NP: Side effects (27%), Getting HIV from vaccine (23%), Fear of needles (20%), Lack of time (11%), Fear of what people will think (8%) | NP: Safety concerns (50%), Physical pain (20%), Inconvenience (11%), Stigmatization (8%) | |  |
| 32.  Jenkins et al., 2000 | Vaccine trial | | HIV | | | Thailand | | | Young men in the army (n= 2670) | Hypothetical | Survey, Cross-sectional, Mixed-methods | Assess WTP | P: Desire to help society (43.2%), Medical care and health insurance (29.8%), Financial incentive (10.9%), family support (39.4%) | P: Altruism (43.2%), Social support (39.4%), Access to Health care (29.8%), Monetary Benefit (10.9%) | |  |
|  |  |  |  |  |  |  |  |  |  |  |  |  | NP: Physical harm (16%), family pressure (15.3%), 'social harm' (15.1%), time constraints (9.5%), having to sign a consent form (9.8%) | NP: Safety concerns (16%), Lack of social support (15.3%), Stigmatization (15.1%), Inconvenience (9.5%) | |  |
| 33.  Kaljee et al., 2007 | Vaccine trial | | Typhoid | | | Vietnam | | | Households with children (n= 461) | Prospective | Survey, Cross-sectional, Mixed-methods | Assess feasibility/WTP | P: Experience with health care, Knowledge of typhoid, satisfaction with informed consent process | P: Access to health care, Knowledge, | |  |
|  |  |  |  |  |  |  |  |  |  |  |  |  | NP: Afraid of injections (43%%), Friends not enrolling (12.4%), Not wanting to participate in a pilot (5.6%) | NP: Physical pain (43%), Lack of social support (12.4%), Safety concerns (5.6%) | |  |
| 34, Kamuya et al, 2015. | Community-based studies: RSV observational study, malaria vaccine trial | | Pediatrics, RSV and malaria | | | Kenya | | | Parents of (healthy) participants in studies (n=45) | Retrospective | Participant observations, semi-structured interviews, focus group discussion, Qualitative | Reasons for 'silent refusal' | Participation: access to care and quality of care, agree with the decision to participate, social/spousal support, peer pressure, positive relationships with researchers. | P: Access to Health Care, Research Outcomes, Peer Enrolment, Social Support, Trust. | |  |
|  |  |  |  |  |  |  |  |  |  |  |  |  | NP: lack of support. | NP: Lack of Social Support. | |  |
| 35.  Karim et al., 2011 | Prevention study | | HIV | | | South Africa | | | Ineligible women in trial (n= 185) | Retrospective | Interviews, Group discussions, Cross-sectional, Qualitative |  | P: High quality care, financial incentives, altruistic motives, preference for sex with gel, increased likelihood of active gel, low risk of discovery, peer pressure | P: Access to Health care, Monetary benefit, Altruism, Personal Health Benefit, Guarantee of confidentiality, Peer enrolment | |  |
| 36.  Khalil et al., 2007 | Medical research | | n/a | | | Egypt | | | Egyptian out-patients (n=15) | Prospective | Semi-structured interviews, Cross-sectional, Mixed methods | Assess attitude/knowledge/concern | P: Trust (20%), ‘benefit for others’ (53.3%) | P: Altruism (53.3%), Trust (20%), | |  |
|  |  |  |  |  |  |  |  |  |  |  |  |  | NP: invasive procedure (90%), lack of certainty (80%), Personal risk (33.3%) | NP: Invasive procedures (90%), Lack of clarity (80%), Safety concerns (33.3%), | |  |
| 37.  Kiawi et al., 2012 | HIV research | | HIV | | | Cameroon | | | Rural plantation population (n= 65) | Hypothetical | Structured observations, focus group discussions, key informant interviews, Cross-sectional, Qualitative | Identify facilitators/barriers to participation | P: Community benefit, More health care, Personal information protected, presence of incentives, community involvement, minimal disruption to daily life | P: Altruism, Access to Health care, Guarantee of confidentiality, Personal benefit, Community involvement, Convenience | |  |
|  |  |  |  |  |  |  |  |  |  |  |  |  | NP: Other research more important, fear of HIV testing, mistrust of researchers, difficult access to medical care/treatment, life disruptions/requirement of behavioral changes | NP: Lack of interest, Safety concerns, Distrust, Inconvenience | |  |
| 38.  Kivouele et al.,2011 | Vaccine trial | | Malaria | | | Congo | | | Congolese public of Brazzaville (n=100) | Hypothetical | Interviews, Cross-sectional, Qualitative | Evaluate knowledge, perception and WTP | P: Trust, Knowledge | P: Trust, Knowledge | |  |
|  |  |  |  |  |  |  |  |  |  |  |  |  | NP: Invasive procedures, | NP: Invasive procedure | |  |
| 39.  Kiwanuka et al., 2004 | Vaccine trial | | HIV | | | Uganda | | | (n= 14,177) | Hypothetical | Questionnaire, Cohort, Mixed-methods | Assess knowledge and WTP | P: Perceived risk, protection from HIV, | P: Perception of risk, Personal Health Benefit | |  |
| 40.  Kiwanuka et al., 2013 | Vaccine trial | | HIV | | | Uganda | | | Fishing community (n=2200) | Hypothetical | Interviews, Cohort, Mixed- methods | Assess HIV risk behavior and WTP | NP: Side effects (43.2%), fear of contracting HIV from vaccine (27%) | NP: Safety concerns (60%) | |  |
| 41.  Kruse-Jarres et al., 2013 | Clinical trial | | Hemophilia | | | USA/South Africa | | | Adult patients/Parents of pediatric patients with hemophilia (n= 74 in SA) | Hypothetical | Online surveys, Cohort, Mixed-methods | Identify factors influencing participation | P: Contribute to research, free gift/money least important, Family/Health professional support | P: Altruism, Monetary benefit, Social support | |  |
| 42.  Kufa et al., 2013 | TB vaccine Trial | | Tuberculosis | | | South Africa | | | HIV+ patients (n= 827) | Hypothetical | Interviews, Questionnaires, Cross-sectional, Qualitative | Assess WTP | NP: Efficacy uncertainty (15%), Side effects (13%), | NP: Efficacy concerns (15%), Safety concerns (13%) | |  |
| 43.  Li et al., 2010 | Clinical trial | | Cancer | | | China | | | Cancer patients (n= 578) | Hypothetical | Questionnaires, Cross-sectional, Mixed-methods | Assess perception towards clinical trials | P: Advanced disease stage (75.6%), Physician recommendation (34%), more therapy chance (27%) free therapy (9%), | P: Need for treatment (75.6%), Advice from physician (34%), Access to health care (30%) | |  |
|  |  |  |  |  |  |  |  |  |  |  |  |  | NP: Not wanting to be a 'guinea pig' (14%), unknown effect of drug (12.6%), side effect unknown (5%), family disagreement (2.2%) | NP: Safety concerns (20%), Efficacy concerns (12.6%), Lack of social support (2.2%) | |  |
| 44.  Li et al., 2010 | Vaccine trial | | HIV | | | China | | | MSM (n= 550) | Hypothetical | Structured questionnaires, Cross-sectional, Mixed method | Assess WTP | P: Help scientists/society (98%), HIV information (87%), HIV counselling/testing (98%), Incentive (92%), Motivation to avoid risky behavior (94%), HIV protection (85%), Family support (41.77%) | P: Altruism (98%), Personal Health Benefit (95%), Social support (41.7%%), | |  |
|  |  |  |  |  |  |  |  |  |  |  |  |  | NP: False-positive HIV test (77%), Efficacy concerns (70%), Weaken body’s ability to fight HIV (64%), Stigmatization (54%), | NP: False-positive testing (77%), Efficacy concerns (70%), Safety concerns (64%), Stigmatization (54%) | |  |
| 45.  Lindegger et al., 2007 | Prevention Study | | HIV | | | South Africa | | | Semi-rural community (n=41) | Hypothetical | Interviews, Cross-sectional, Mixed-methods | Asses knowledge, attitudes and WTP | P: Altruism (44%), Material benefit (17%) | P: Altruism (44%), Monetary benefit (17%) | |  |
|  |  |  |  |  |  |  |  |  |  |  |  |  | NP: Lack of information (27%), Cultural/Religious issues (22%), Vaccination causes illness (15%), Laziness (15%), Logistics (15%), Social concerns (5%), Distrust (5%) | NP: Lack of clarity (27%), Cultural insensitivity (22%), Safety concerns (15%), Lack of interest (15%), Inconvenience (15%), Stigmatization (5%), Distrust (5%) | |  |
| 46.  Lobato et al., 2014 | Clinical trial | | n/a | | | Brazil | | | Participants in a functional food study (n= 143) | Prospective | Interviews Cross-sectional, Mixed- methods | Assess WTP | P: Medical treatment (92%), Researcher influence (88%), Influence of family/friends (62%), | P: Access to Health Care (92%), Trust (88%), Social support (62%), | |  |
|  |  |  |  |  |  |  |  |  |  |  |  |  | NP: Exams (70%), | NP: Invasive procedures (70%) | |  |
| 47.  Loh et al., 2012 | Qigong exercise trial | | Cancer | | | Malaysia | | | Cancer patients (n=197) | Retrospective | Telephone interviews, Cross-sectional, Mixed-methods | Assess barriers to participation | NP: Job commitment (17%), Family commitments (25%), Illness (17%), Transportation (25%), Lost interest (17%) | NP: Inconvenience (42%), Lack of social support (25%), Worsening of Medical condition (17%), Lack of Interest (17%) | |  |
| 48.  Martinez-Andrade et al., 2014 | Pilot intervention Study | | Childhood obesity | | | Mexico | | | Parents of participants (n=306) | Prospective | Interviews, Cross-sectional, Mixed-methods | Intervention | NP: Transportation cost, Time | NP: Inconvenience, Costs, | |  |
| 49.  Macphail et al., 2012 | Drug Trial | | HIV | | | South Africa | | | HIV+ women participating in treatment trial (n=31) | Prospective | Interviews, Cross-sectional, Qualitative | Assess motivations to participate/adhere to treatment | P: Seeking high-quality health care, Altruism | P: Access to Health care, Altruism | |  |
| 50.  Mahomed et al., 2008 | Vaccine trial | | Tuberculosis | | | South Africa | | | Adolescents (n= | Hypothetical | Questionnaires, Interviews, Cross-sectional, Qualitative | Assess WTP | P: Parents:  Personal health benefits, Personal Benefits, Adolescents:  Knowledge, Altruism | P: Personal Benefits, Personal Health Benefits, Knowledge, Altruism | |  |
|  |  |  |  |  |  |  |  |  |  |  |  |  | NP: Safety concerns, invasive procedures (e.g. blood draw), Exploitation | NP: Safety concerns, Invasive procedures, Distrust | |  |
| 51. Mamotte et al, 2016. | Clinical trial | | HIV | | | South Africa | | | Female HIV patients (n=100) | Cross sectional | Questionnaires, interviews, Mixed-methods | WTP, reasons for WTP, | P: want to participate (97% indicated this, 90% indicated this influenced participation 'a lot'), needed health care (89%, 85% a lot), trusted researcher (84%, 92%), ill health (53%, 70%), needing money (13%), no other choice (12%), pleasing other person such as researcher, partner or someone else (12%) influenced by others (threats, pressure, advice): partner, family, care provider, trial staff. | P: Access to Health Care, Trust, Personal Health Benefits, Monetary Benefits, Social Support, Advice from Physician | |  |
|  |  |  |  |  |  |  |  |  |  |  |  |  | NP: influenced by others (threats, pressure, advice): partner, family, care provider, trial staff | NP: lack of social support | |  |
| 52.  Mbunda et al., 2014 | Vaccine trial | | HIV | | | Tanzania | | | Adolescents visiting a clinic (n=450) | Hypothetical | Questionnaire, Cross-sectional, Mixed- methods | Assess WTP | P: Knowledge of clinical trials, social support for decision making | P: Knowledge, Social support | |  |
|  |  |  |  |  |  |  |  |  |  |  |  |  | NP: Safety (25%), Fear of HIV from vaccine (15%), Side effects (5%), Lack of knowledge, Lack of social support | NP: Safety concerns (40%), Lack of knowledge, Lack of social support | |  |
| 53.  McGrath et al., 2001 | Vaccine trial | | HIV | | | Uganda | | | Male Members of Ugandan military (n=1182) | Hypothetical | Surveys, Cross-sectional, Mixed- methods | Assess knowledge and WTP | P: HIV protection (35%), HIV cure (27%), HIV testing (94%) | P: Personal Health Benefits (35%), Access to Health Care (94%), Altruism (27%) | |  |
|  |  |  |  |  |  |  |  |  |  |  |  |  | NP: Fear of side-effects (26%), getting placebo (19.5%), the vaccine won’t work (28%) | NP: Safety concerns (26%), Placebo concerns (19.5%), Efficacy concerns (28%) | |  |
| 54. Meneguin et al, 2016. | Clinical trial | | Hypertension and coronary disease | | | Brazil | | | CVD Patients (n=19) | Cross sectional | Focus group discussions, Qualitative | Perception of research, reasons for WTP | P: personal health benefit, exchange relation (mix between own benefit, benefit others scientific progress, continued treatment at health facility), access to health care and better health care, altruism and contribution to science progress. | P: Personal Health Benefit, Altruism, Access to Health Care | |  |
| 55.  Mensch et al., 2013 | Placebo vaginal gel (intervention study) | | STI's | | | India | | | Female sex workers | Hypothetical | Respondent-driven sampling, Surveys, Cross-sectional, Mixed-methods | Assess WTP | P: Financial reimbursement (34.5%), free health care (99%), HIV testing (99.2%), STI testing/treatment (98.9%), Information about HIV prevention (98.3%), Free family planning/contraceptives (86.3%), Help test a product for HIV prevention (97.8%), partner referral for testing/treatment (58.9%) | P: Access to health care (99%), Personal Health Benefits (99%), Knowledge (98.3%), Altruism (97.8%), Personal benefit (58.9%), Monetary benefit (34.5%), | |  |
|  |  |  |  |  |  |  |  |  |  |  |  |  | NP: Husband/partner disapproval (23.3%), family disapproval (24.9%), fear of doctors/nurses (17.1%), HIV contraction worry (13.5%), fear of trouble with police (33.4%), worried that community assumes HIV infection (11.1%), Harmful effects of product (6.4%) | NP: Stigmatization (33.4%), Lack of social support (24%), Distrust (17.1%), Safety concerns 6.4%), | |  |
| 56.  Meque et al., 2014 | Vaccine trial | | HIV | | | Mozambique | | | Participants in HIV incidence/WTP studies (n= 1116) | Hypothetical | Surveys, Cross-sectional, Qualitative | Assess WTP | P: Finding an HIV cure, Helping society, Family/social support, | P: Altruism, Personal Health Benefits, Social Support | |  |
|  |  |  |  |  |  |  |  |  |  |  |  |  | NP: Personal risk (main deterrent), ‘People will think I have HIV’ | NP: Safety concerns, Stigmatization | |  |
| 57.  Mfutso-Bengo et al., 2015 | Biomedical research | | n/a | | | Malawi | | |  | Retrospective | Surveys, Cross-sectional, Qualitative | Assess motivations in WTP | P: Access to health care (major reason), financial/material gain, proper diagnosis | P: Access to health care, Monetary benefit | |  |
| 58.  Mfutso-Bengo et al., 2008 | Biomedical research | | n/a | | | Malawi | | |  | Hypothetical | Focus-group discussions, Cross-sectional, Qualitative | Assess refusals | NP: Failure to follow traditional customs, poor informed consent procedures, ignorance, fear of strangers, lack of cultural sensitivity, poor timing, previous bad experience | NP: Cultural insensitivity, Lack of Understanding, Distrust, Inconvenience, Previous negative experience | |  |
| 59.  Mfutso-Bengo et al., 2008 | Biomedical research | | n/a | | | Malawi | | |  | Hypothetical | Focus group discussions, Cross-sectional, Qualitative | Assess reasons for enrolment | P: Access to health care (main reason) | P: Access to Health Care | |  |
| 60. Montgomery et al, 2015. | Clinical trial | | HIV  (Pre-Exposure Prophylaxis) | | | South Africa | | | (Healthy) Women (n=102) and male partners (n=22) | Cross sectional | In-depth interviews, serial ethnographic interviews, focus group discussions, Qualitative | Assess influence of male partners on women's participation | P: three dimensions of 'understanding' by male partners: comprehension, support/agreeability, and acceptance of increased access of women to health information, knowledge and preventive products | P: Social Support | |  |
|  |  |  |  |  |  |  |  |  |  |  |  |  | NP: lack of aforementioned dimensions by male partner | NP: Lack of Social Support | |  |
| 61.  Murthy et al., 2012 | RCT | | Cancer | | | India | | | Oral cancer patients (n= 498) | Prospective | Surveys, Cohort, Mixed-methods | Assess reasons for non-enrolment | NP: Inconvenience (32%), lack of interest (16.8%), | NP: Inconvenience (32%), Lack of interest (16.8%) | |  |
| 62.  Newman et al., 2014 | Vaccine trials | | HIV | | | India | | | MSM (n=400) | Hypothetical | Interviews, Cross-sectional, Qualitative | Assess WTP | P: Free medical treatment, financial incentive, life insurance, access to vaccine after trial | P: Access to Health care (90%), Personal Health Benefit (90%), Monetary benefit (60%), | |  |
|  |  |  |  |  |  |  |  |  |  |  |  |  | NP: Side effects, large distance to trial site, | NP: Safety concerns (80%) ,Inconvenience (65%) | |  |
| 63.  Nyamathi et al., 2007 | Vaccine trial | | HIV | | | India | | | Community members (n=112) | Hypothetical | Semi-structured interviews, Cross-sectional, Qualitative | Assess perceptions of participation in trials | P: Altruism, Desire for HIV protection | P: Altruism, Personal Health Benefit, | |  |
|  |  |  |  |  |  |  |  |  |  |  |  |  | NP: Sero-conversion, Confidentiality concerns, Compensation concerns, Temptation to risky behavior | NP: Confidentiality concerns, Temptation to unsafe behavior, Insufficient compensation | |  |
| 64.  Nyblade et al., 2011 | Vaccine trial | | HIV | | | Kenya | | | Research center (HIV vaccine and epidemiological centers) population (n=133) | Prospective | Interviews, Cross-sectional, Qualitative | Assess stigma as a barrier | NP: Being perceived as HIV positive, Stigmatization, Social harm, confidentiality concerns | NP: Stigmatization, Confidentiality concerns | |  |
| 65.  Ogendo et al., 2012 | Vaccine trial | | HIV | | | Kenya | | | High-risk individuals (n=241) | Hypothetical | Surveys/Interviews, Cross-sectional, Qualitative | Identify recruitment strategies | P: Receiving HIV prevention education (47.8%), Receiving compensation, Helping to find a cure (11.8%) | P: Knowledge, Monetary benefit | |  |
|  |  |  |  |  |  |  |  |  |  |  |  |  | NP: Fear of lack of confidentiality (29.9%), HIV testing concerns (17.5%), No personal gain, Stigmatization | NP: Confidentiality concerns, False-positive testing, Lack of interest, Stigmatization | |  |
| 66.  Okall et al., 2014 | Prevention studies | | HIV | | | Kenya | | | MSM (n= 66) | Hypothetical | Interviews,Structured Survey, Cross-sectional, Qualitative | Assess WTP | P: HIV education (35.3%), HIV testing/counseling (17.7%), finding an HIV cure (17.7%), Incentives (7.8%), Free HIV treatment/care (2%), being part of a social group (2%), having friends enroll (2%) | P: Knowledge, (35.3%) Personal Health Benefits (19%), Altruism (17.7%), Feeling of community (2%), Peer enrolment (2%) | |  |
|  |  |  |  |  |  |  |  |  |  |  |  |  | NP: Confidentiality concerns (64.7%), Time away from job (5.9%), Losing job (3.9%), Moving out of area (3.9%), New medical costs not covered in study (3.9%), HIV false positive testing (2%), Partner/Spouse refusal (2%), Taking blood (2%) | NP: Confidentiality concerns (64.7%), Inconvenience (10%), Personal costs (3.9%), False positive testing (2%), Lack of social support (2%), Physical pain (2%) | |  |
| 67. O'Neill, 2016. | Clinical trial | | Malaria | | | the Gambia | | | Inhabitants of a village (assumed to be healthy) (n=30) | Cross sectional | In-depth interviews, participant observation, information conversation, group discussion, Qualitative | Exploration of anxieties in WTP, focus on blood-taking | NP: fear of loss of strength through blood loss (depleting life-force and body strength), fear of need to be hospitalized due to lack of blood (and then require someone else's) | NP: Safety Concerns, Worsening of Medical Condition | |  |
| 68.  Pare-Toe et al., 2013 | Clinical Trial | | Malaria | | | Burkina Faso | | | Parents/Guardians of potential child participants | Hypothetical | Surveys, Cross-sectional, Mixed-methods | Assess relevance of informed consent process | P: Access to free health care (70.4%) | P: Access to Health care (70.4%) | |  |
| 69.  Perisse et al., 2000 | Vaccine trial | | HIV | | | Brazil | | | MSM HIV - (n= 815) | Hypothetical | Surveys, Cross-sectional, Mixed methods | Assess WTP | P: Contribute to ending AIDS (96%), Helping community (87.7%), Free snack/bus ticket (13.5%) | P: Altruism (96%), Monetary benefit (13.5%) | |  |
|  |  |  |  |  |  |  |  |  |  |  |  |  | NP: Vaccine-induced HIV (77%), False positive testing (61%), Do not want to be a guinea pig (47.5%), Side effects (45%), Vaccine failure (36%), Distrust of Brazilian/US government/drug companies/researchers (22%) | NP: Safety concerns (77%), False-positive testing (61%), Efficacy concerns (36%), Distrust (70%) | |  |
| 70.  Qiu et al., 2013 | Observational Cohort study | | n/a | | | China | | | Pregnant women (n= 526) | Hypothetical | Survey, Cross-sectional, Qualitative | Assess WTP | P: Minimally invasive procedures (97%), Assessment of childhood development (ranked 9/10), Priority service at doctor (ranked 8.9/10), Pregnancy/Infant nutrition counseling (ranked 8.3/10), Concession on medical fees (ranked 8.2/10), Registration fee waived (ranked 7.2/10), Free prenatal classes (ranked 7/10), Free parking (ranked 6.4/10) | P: Non-invasive procedures (97%), Access to Health Care, Personal benefit, Knowledge, Monetary Benefit | |  |
| 71.  Ramjee et al., 2010 | Intervention study | | HIV | | | South Africa | | | Women (n= 7046) | Retrospective | Interviews,  Cross-sectional, Mixed-methods | Describe experiences | P: HIV education, Ancillary care, Monetary benefits, Fast access to health care | P: Knowledge, Access to Health care, Monetary benefits | |  |
| 72.  Reynolds et al., 2011 | Treatment efficacy trial | | Malaria/  HIV | | | Tanzania | | | Participants in Malaria/HIV trials | Prospective | Interviews, Cohort, Qualitative | Assess recruitment implications | P: Free medical care (most important), Financial compensation | P: Access to Health Care, Monetary Benefit | |  |
|  |  |  |  |  |  |  |  |  |  |  |  |  | NP: ‘true’ aim of study, fear of blood taking | NP: Distrust, Physical pain | |  |
| 73.  Rodrigues et al., 2013 | Research | | HIV | | | India | | | HIV+ adults (n= 173) | Hypothetical | Surveys, Cross-sectional, Mixed-methods | Assess WTP/Knowledge | P: Individual health benefits (66%), Altruism (50%), Non-invasive procedures (12%), Unaware of ability to refuse (23%) | P: Personal Health Benefits (66%), Altruism (50%), Non-invasive procedures (12%), Unaware of voluntariness (23%) | |  |
|  |  |  |  |  |  |  |  |  |  |  |  |  | NP: Privacy concerns (66%), Time constraints (50%) | NP: Confidentiality concerns (66%), Inconvenience (50%) | |  |
| 74.  Rohra et al., 2009 | Case-control Study | | Pre-eclampsia | | | Pakistan | | | Participants in a primigravidae study (n= 1665) | Retrospective | Interviews, Cross-sectional, Mixed-methods | Reasons for refusal/drop-out | NP: Family refusal (34.4%), Fear of pricking blood (20.9%), Not interested in research (20.1%), Planning to deliver in a different place (8.6%), | NP: Lack of social support (34.4%), Physical pain (20.9%), Lack of interest (20.1%), Inconvenience (8.6%) | |  |
| 75.  Ruan et al, 2009 | Circumcision (prevention study) | | HIV | | | China | | | MSM (n= 328) | Hypothetical | Interviews, Cohort, Mixed- methods | Assess WTP | P: Contribution to AIDS research (99%), free circumcision (2%), free HIV counseling/testing (100%) | P: Personal health benefits (100%), Altruism (99%) | |  |
|  |  |  |  |  |  |  |  |  |  |  |  |  | NP: Concern about pain (46.5%), Ineffectiveness concerns (28.5%), Infection (12.7%), Reduced sexual pleasure (5.3%), Scars/Appearance (2.5%), Others think I am at risk (2.5%) | NP: Physical pain (46.5%), Efficacy concerns (28.5%), Safety concerns (12.7%), Stigmatization (2.5%) | |  |
| 76.  Ruzagira et al., 2009 | Vaccine trial | | HIV | | | Uganda | | | Community cohort, already participating in a vaccine trial | Hypothetical | Questionnaires, Cross-sectional, Mixed- methods | Assess WTP | NP: Chance of receiving placebo (22%), Delaying pregnancy (75%), Large blood draws (40%) , Long trial duration (2%), monthly visits (2%), | NP: Non-compliance to terms of research (75%), Invasive procedures (40%), Inconvenience (2%) | |  |
| 77.  Sahay et al., 2005 | Vaccine trial | | HIV | | | India | | | STI clinic patients (n=349) | Hypothetical | Semi-structured questionnaires, Cross-sectional, Qualitative | Assess WTP | P: Insurance (51%), Monetary benefit (32%), Importance of vaccine for self (38%), Altruism (61%) | P: Altruism (61%), Monetary benefit, (32%) Personal Health benefit (45%) | |  |
|  |  |  |  |  |  |  |  |  |  |  |  |  | NP: Serious Adverse effects (55%), Refusal by partner (56%) | NP: Safety concerns (55%), Lack of social support (56%) | |  |
| 78.  Sexton et al. 2011 | Epidemiological Study | | Cancer | | | Mexico/USA | | | Female breast cancer patients (n=914) | Prospective | Interviews, Cross-sectional, Mixed-methods | Assess WTP, refusal to participate | NP: Lack of Interest, Overwhelmed with diagnosis, Family disapproval (10%), | NP: Lack of interest, Overwhelmed, Lack of social support | |  |
| 79.  Shaffer et al., 2006 | Clinical Trial | | HIV | | | Kenya | | | Potential participants, clinicians/researchers (n= 89) | Hypothetical | Focus group discussions, Cross-sectional, Qualitative | Assess WTP/Ethical issues | P: Continued (life-long) drug therapy, Financial incentive, Health/Ancillary care, Moral obligation | P: Monetary benefit, Access to Health care, altruism | |  |
| 80. Shanks et al, 2015. | Clinical trial | | Mental Health | | | Czech Republic, Democratic Republic of Congo | | | (healthy) Community members (medical professionals, key community members, target population) | Hypothetical | Group discussions, Qualitative | Acceptability and feasibility of the proposed study design, informing the community of the study to promote participation and understanding of study in community. | P DRC: good and culturally adapted information, moral obligation [to study team because of previous health care provided], rarely were incentives or compensation mentioned, support by research team (through home visits). | P DRC: Research Outcome, Access to Health Care, Trust, Personal Health Benefits. | |  |
|  |  |  |  |  |  |  |  |  |  |  |  |  | NP DRC: loss of support, insufficient confidentiality | NP DRC: Lack of Social Support, Confidentiality Concerns. | |  |
| 81.  Sikateyo et al., 2012 | Entero-toxigenic vaccine Trial | | n/a | | | Zambia | | | Participants in trials | Prospective | Ethnography, Qualitative | Understand patient's consent | P: Enhance personal and family well-being | P: Personal benefit | |  |
| 82.  Smit et al., 2006 | Vaccine trial | | HIV | | | South Africa | | | Community members (n = 198) | Hypothetical | Surveys, Cross-sectional, Mixed-methods | Assess WTP | P: Money (87%), Free Medical care (93%), Knowledge you are fighting HIV/AIDS (100%), receiving antiretroviral treatment (97%), Protection (100%) | P: Altruism (100%), Personal Health Benefit (100%), Access to Health Care (97%), Monetary benefit (87%), | |  |
|  |  |  |  |  |  |  |  |  |  |  |  |  | NP: Frequent clinic visits (94%), Frequent blood draw (94%), Frequent vaccinations (94%), Side-effects (98%), receiving AIDS from vaccine (96%), People think you have HIV (94%) familial concerns (94%), Positive test to HIV (96%), Time (96%) | NP: Inconvenience, Physical pain, Safety concerns, Stigmatization, Lack of social support, False Positive test | |  |
| 83.  Suhadev et al., 2009 | Vaccine trial | | HIV | | | India | | | Members of Risk groups (n=501) | Hypothetical | Interviews, questionnaires, Cross-sectional, Mixed- methods | Assess WTP | P: Less chance of HIV infection (95%), Common good of India (93%), HIV protection (90%), Help researchers (94%), Reduce threat of HIV infection (86%), Make HIV preventable (86%) | P: Personal Health Benefit (95%), Altruism (93%) | |  |
|  |  |  |  |  |  |  |  |  |  |  |  |  | NP: Uncertainties (89%), Side effects (70%), Efficacy concerns (89%), Effect on lifestyle (84%), | NP: Lack of clarity (89%), Safety Concerns (70%), Efficacy concerns (89%), Inconvenience (84%) | |  |
| 84.  Suhadev et al., 2006 | Vaccine trial | | HIV | | | India | | | Risk populations (n= 112) | Hypothetical | Structured questionnaires, Cross-sectional, Mixed- methods | Assess WTP | P: Altruism (M:97%, F:100%), Desire for HIV protection (M:92%, F:89%), Support researchers (M:82%, F:91%), | P: Altruism (98.5%), Personal Health Benefit (90.5%), | |  |
|  |  |  |  |  |  |  |  |  |  |  |  |  | NP: Uncertainties (43%), Want real vaccine with certainty (26%), Efficacy concerns (35%), Side effects (32%), Effect on lifestyle (31%) | NP: Placebo concerns (26%), Efficacy concerns (35%), Safety concerns (32%), Stigmatization (31%), Lack of clarity (43%) | |  |
| 85. Téguété et al, 2016 | Vaccine trial | | HPV/  Cancer | | | Mali | | | Women with cervical cancer (n=235) | Hypothetical | Questionnaires, Qualitative | Serologic study, WTP | P: Permission from male spouse or head of household, | P: Social Support | |  |
| 86.  Tharawan et al., 2001 | Microbicide/Prevention Study | | HIV/STI's | | | Thailand | | | Women (n= 370) | Hypothetical | Structured questionnaires, Cross-sectional, Mixed-methods | Assess WTP/Knowledge | P: HIV testing (82.2%), Partner receives testing (81.2%), Confidentiality guarantee (75.9%), Approved by Thai ethics committee (75.7%), Free check-ups (73.2), Community knowledge (73%), Doing something good for women (72.2%), Counselling (70.3%), Convenience (61%), Free contraceptives (64.1%), Reimbursement (34.1%) | P: Personal Health Benefit (82.2%), Altruism (75%), Access to Health care (73.2%), Trust (75.8%), Social support (73%), Convenience (61%), Monetary benefit (34.1%) | |  |
|  |  |  |  |  |  |  |  |  |  |  |  |  | NP: Risk for adultery by husbands (45.9%), Need husband’s consent (42.2%), Husband thinks it is a bad idea (33%), Side effect (35.4%), Placebo (31.1%), Foreign product (17%), Microbicide noticeable during sex (15.7%), Pain from needles (15.1%), Not at risk for HIV (12.4%), Effectiveness unsure (10%), Neighbors may gossip (9.5%) | NP: Lack of social support (40%), Safety concerns (35%), Placebo concerns (31.1%), Distrust (17%), Lifestyle effect (15.7%), Physical Pain (15.1%), Efficacy concerns (10%), No perceived need (12.4%), Stigmatization (9.5%) | |  |
| 87.  Thienkrua et al., 2014 | Prevention Study | | HIV | | | Thailand | | | MSM (n= 1744) | Hypothetical | Interviews, Cohort, Mixed- methods | Assess WTP | P: To reduce risk of HIV in MSM (98%), regular sexual health check-up (97.2%), Learn more about HIV (97.5%), Help prevent HIV in Thailand (97%), Contribute to science/knowledge (97.8%), Regular HIV test (96%), Curiosity (93%), HIV protection (90%), Convenience (88%), Concern about risk of HIV (79%), Knowledge of previous success in trial (78%), Financial Compensation (29%) | P: Altruism (98%), Access to health care (97%), Personal Health Benefits (93%), Knowledge (93%), Convenience (88%), Perceived as being at risk (79%), Trust (78%), Monetary benefit (29%) | |  |
|  |  |  |  |  |  |  |  |  |  |  |  |  | NP: Not wanting rectal examination/fluid collection (19%), Placebo concerns (22%), Time (14%), Blood (8%), Family/Friend pressure (6%), don’t want to take HIV tests (3%) | NP: Placebo concerns (22%), Invasive procedures (19%), Inconvenience (14%), Physical pain (8%), Lack of social support (6%), Fear of health status (3%) | |  |
| 88.  Udrea et al., 2009 | Novel therapy | | Rheumatoid Arthritis (RA) | | | Romania | | | RA patients (n= 96) | Hypothetical | Questionnaires Surveys, Cross-sectional, Mixed-methods | Assess WTP | P: Improve health (90%), Gather new data on disease (83%) Improve health of others (77%), Trust in physician (72%), Free access to laboratory tests (63%), Payment (23%), Please doctor (17%), | P: Personal benefit (90%), Altruism (83%), Trust (72%), Access to Health care (63%), Monetary benefit (23%), | |  |
|  |  |  |  |  |  |  |  |  |  |  |  |  | NP: Adverse effects (73%), limited experience with product (53%), getting a placebo (50%), availability of alternatives (43%), treatment discontinuation (33%), doctor reimbursement (13%),), blood draw (13%), Disruption of daily routine (13%), Transportation problems (13%) | NP: Safety concerns (73%), Placebo concerns (50%), Non-compliance to terms of research (33%), No perceived need (43%), Lack of Knowledge (53%), Physical pain (13%), Distrust (13%), Inconvenience (13%) | |  |
| 89.  Vieira de Souza et al., 2003 | Vaccine trial | HIV | | | Brazil | | | MSM w/ history of commercial sex (n= | | Hypothetical | Questionnaires, Cohort, Mixed- methods | Assess WTP | P: Human concerns/solidarity (60.8%), protection against HIV (44.2%), have sex without being concerned about AIDS (14.4%), trust of scientific achievements (18.9%) | | P: Altruism (60.8%), Personal Health Benefits (44.2%), Trust (18.9%) | |
|  |  |  |  |  |  |  |  |  |  |  |  |  | NP: Afraid of being used as a guinea pig (13.2%), Adverse effects (33.8%), AIDS contraction from vaccine (21%), Not enough information (34.6%) | | NP: Lack of Knowledge (34.6%), Safety concerns (33.8%), Distrust (13.2%), | |
| 90.  Woodsong et al., 2012 | Microbicide/ Prevention Study | | | HIV/STI | | | Malawi & Zimbabwe | Women & their partners in trial | | Prospective | Semi-structured interviews, Cohort, Qualitative | Assess misconceptions | P: HIV testing/counselling (F: 71%, M:42%), Altruism (F: 27%, M:30%), Access to Health services (F: 17%, M: 25%) | | P: Personal Health Benefit (F: 71%, M:42%), Altruism (F: 27%, M:30%), Access to Health Care (F: 17%, M: 25%) | |
| 91.  Wu et al., 2015 | Biomedical research | | | n/a | | | USA, China | Patients (n= 278, China) | | Hypothetical | Surveys, Cross-sectional, Mixed methods, | Compare WTP | P: Improving health (35%), Helping others with same condition (25%), Advancing science (25%), Priority access to specialists (8%), Shorter wait for care (5%), Free medical care (2%), Payment (1%), | | P: Altruism (50%) , Personal Health Benefit (42%) , Access to Health Care (2%), Monetary Benefit (1%) | |
|  |  |  |  |  |  |  |  |  |  |  |  |  | NP: Safety concerns (62%), Personal Costs (6%), Distrust (5%), Confidentiality concerns (4%), Inconvenience (4%), | | NP: Safety concerns (62%), Personal costs (6%) Distrust (5%), Confidentiality concerns (4%), Inconvenience ( | |
| 92.  Ye et al., 2014 | Prevention study | | | HIV | | | China | Female sex workers (n= 405) | | Hypothetical | Questionnaires, Cross-sectional, Mixed- methods | Assess interest/  WTP | NP: Side effects (89.5%), Not perceived as at risk (50.9%), Not necessary or effective (36.8%), Objection from family (31.6%), Discrimination (17.5%) | | NP: Safety concerns (89.5%), No perceived need (50.9%), Efficacy concerns (36.8%), Lack of 2008) (31.6), Stigmatization (17.5%) | |
| 93.  Yin et al., 2008 | Vaccine trial | | | HIV | | | China | IDU's (n= 401) | | Hypothetical | Questionnaires interview, Cross-sectional, Mixed- methods | Assess WTP | P: Family support (76.2%), Effective vaccine development (100%), Contribute to control/prevention of AIDS (100%), Gain knowledge (98.3%), Free HIV counseling/testing (98.3%), Incentive (99.3%), Motivation to avoid risky behavior (98%), HIV protection (99%) | | P: Social support (76.2%), Personal Health Benefit (98.3%), Altruism (100%), Knowledge (98.3%), Monetary benefit (99.3%), Motivation to avoid risky behavior (98%) | |
| 94. Zvonareva et al, 2015. | Clinical trial | | | CVD | | | Russia | Patients (men and women) n=21) | | Cross sectional | In depth semi-structured interviews,Qualitative | Risk benefits assessment of participants in trial participation. | P: access to (additional/regular) health care, access to health information, gaining health knowledge, provided support for self-discipline for regular drug taking, relationship with physician-investigator, | | P: access to health care, personal health benefit, researcher attitude, trust knowledge | |

P: participation, NP: non-participation, PMTCT: prevention of mother-to-child transmission, WTP: willingness to participate, RSV: respiratory syncytial virus, STI: sexually transmitted infection, PCT: placebo-controlled trial, IDU: intravenous drug users, MSM: Men who have sex with men, CVD: Cardiovascular disease

Supplementary Table 2 Ranking of reasons per article

| Abrams et al., 2011 | 1. Altruism  2. Access to Health Care  3. Monetary benefit | 1. Physical Pain  2. Fear of Health Status |
| --- | --- | --- |
| Ahram et al., 2014 | 1. Cultural Acceptability  2. Result Availability  3. Allowing Withdrawal | 1. Confidentiality concerns  2. Lack of clarity  3. Re-contact |
| Al-Amad et al., 2014` | 1. Altruism  2. Advice from Physician  3. Social Support  4. Access to Health Care  5. Monetary benefit | 1. Invasive procedures  2. Safety concerns  3. Physical Pain  4. Inconvenience  5. Lifestyle effect  6. Confidentiality concerns |
| Anjos et al. |  | 1. Worsening of Medical condition  2. Inconvenience |
| Asiki et al., 2013 | 1. Personal Health Benefits  2. Knowledge  3. Access to health care | 1. Inconvenience  2. Safety concerns |
| Bakari et al., 2013 |  | 1. Lack of social support  2. Non-compliance to terms of research |
| Boniphace et al., 2010 |  | 1. Inconvenience  2. Cultural insensitivity  3. Lack of Knowledge |
| Bouida et al., 2016 | 1. Altruism  2. Community Involvement | 1. Safety Concerns  2. Distrust  3. Lack of Interest |
| Burt et al., 2013 | 1. Altruism  2. Access to Health Care  3. Monetary Benefit | 1. Insufficient Compensation  2. Safety concerns  3. Confidentiality concerns  4. Distrust |
| Chu et al., 2013 | 1. Altruism  2. Guarantee of confidentiality  3. Social support  4. Monetary benefit | 1. Stigmatization  2. Safety concerns |
| Deschamps et al., 2014 | 1. Personal Health Benefit | 1. Safety concerns  2. Fear of Health status  3.Stigmatization  4. Lack of Social Support  5. Non-compliance to terms of research |
| Djomand et al., 2008 |  | 1. Safety concerns  2. Lack of social support  3. False-positive testing  4. Stigmatization  5. Non-compliance to terms of research |
| Dong et al., 2014 | 1. Personal Health Benefits  2. Altruism | 1. Safety concerns  2. Lack of knowledge  3. No perceived need |
| Doshi et al., 2013 | 1. Monetary benefit (healthy)  2. Altruism (healthy)  3. Access to health care (healthy)  4. Knowledge (healthy)  5. Personal health benefits (healthy) |  |
| Farquhar et al., 2006 |  | 1. Safety concerns  2. Lack of social support  3. Stigmatization  4. False-positive test results |
| Fincham et al., 2010 | 1. Monetary benefit  2. Social Support  3. Trust  4. Personal health benefit | 1. Personal costs  2. Safety concerns  3. Inconvenience  4. Stigmatization |
| Gitanjali et al., 2003 | 1. Altruism  2. Personal Health Benefits  3. Trust | 1. Invasive procedures  2. Safety concerns  3. Fear of health status  4. Inconvenience  5. Lack of Interest  6. Lack of Understanding |
| Groth et al., 2016 | 1. Altruism  2. Community Involvement  3. Research Outcomes |  |
| Jaspan et al., 2006 | 1. Altruism  2. Personal Health Benefits  3. Access to Health care  4. Monetary benefit | 1. Safety concerns  2. Physical Pain  3. Inconvenience  4. Stigmatization |
| Jenkins et al., 2000 | 1. Altruism  2. Social Support  3. Access to Health Care  4. Monetary Benefit | 1. Safety concern  2. Lack of social support  3. Stigmatization  4. Inconvenience |
| Khalil et al., 2007 | 1. Altruism  2. Trust | 1. Invasive Procedures  2. Lack of clarity  3. Safety concerns |
| Kiwanuka et al., 2013 |  | 1. Safety Concerns |
| Kufa et al., 2013 |  | 1. Efficacy concerns  2. Safety concerns |
| Li et al., 2010 | 1. Need for treatment  2. Advice from physician  3. Access to Health Care | 1. Safety concerns  2. Efficacy concerns  3. Lack of social support |
| Li et al., 2010 | 1. Altruism  2. Personal Health Benefits  3. Social Support | 1. False-positive testing  2. Efficacy concerns  3. Safety concerns  4. Stigmatization |
| Lindegger et al., 2007 | 1. Altruism  2. Monetary benefit | 1. Lack of clarity  2. Cultural insensitivity  3. Safety Concerns  4. Lack of interest  5. Inconvenience  6. Stigmatization  7. Distrust |
| Lobato et al., 2014 | 1. Access to Health Care  2. Trust  3. Social Support | 1. Invasive procedures |
| Loh et al., 2012 |  | 1. Inconvenience  2. Lack of Social Support  3. Worsening of Medical condition  4. Lack of Interest |
| Mamotte et al., 2016 | 1 Access to Health Care  2. Trust  3. Monetary Benefit  4. Social Support/Advice from Physician |  |
| McGrath et al., 2001 | 1. Personal Health Benefit  2. Access to Health Care  3. Altruism | 1. Safety concerns  2. Placebo concerns  3. Efficacy concerns |
| Mensch et al., 2013 | 1. Access to Health Care  2. Personal Health Benefits  3. Knowledge  4. Altruism  5. Personal benefit  6. Monetary benefit | 1. Stigmatization  2. Lack of Social Support  3. Distrust  4. Safety concerns |
| Murthy et al., 2012 |  | 1. Inconvenience  2. Lack of Interest |
| Newman et al., 2014 | 1. Access to Health Care  2. Personal Health Benefit  3. Monetary benefit | 1. Safety concerns  2. Inconvenience |
| Okall et al., 2014 | 1. Knowledge  2. Personal health benefit  3. Altruism  4. Social Support | 1. Confidentiality concerns  2. Inconvenience  3. Personal costs  4. False positive testing  5. Lack of Social Support  6. Physical pain |
| Pare-Toe et al., 2013 | 1. Access to Health care |  |
| Perisse et al., 2000 | 1. Altruism  2. Monetary Benefit | 1. Safety concerns  2. False-positive testing  3. Efficacy concerns  4. Distrust |
| Qiu et al., 2013 | 1. Non-invasive procedures  2. Access to Health Care  3. Personal benefit  4. Knowledge  5. Monetary benefit |  |
| Rodrigues et al., 2013 | 1. Personal Health Benefit  2. Altruism  3. Non-invasive procedures  4. Unaware of voluntariness | 1. Confidentiality concerns  2. Inconvenience |
| Rohra et al., 2009 |  | 1. Lack of Social Support  2. Physical Pain  3. Lack of Interest  4. Inconvenience |
| Ruan et al, 2009 | 1. Personal health benefit  2. Altruism | 1. Physical pain  2. Efficacy concerns  3. Safety concerns  4. Stigmatization |
| Ruzagira et al., 2009 |  | 1. Non-compliance to terms of research  2. Invasive procedures  3. Inconvenience |
| Sahay et al., 2005 | 1. Altruism  2. Monetary Benefit  3. Personal Health Benefit | 1. Safety Concerns  2. Lack of Social Support |
| Smit et al., 2006 | 1. Altruism  2. Personal Health Benefit  3. Access to Health Care  4. Monetary Benefit | 1. Stigmatization  2. Inconvenience  3. Lack of Social Support  4. Physical Pain  5. Safety Concerns  6. False-positive Testing |
| Suhadev et al., 2009 | 1. Personal Health Benefits  2. Altruism | 1. Lack of Clarity  2. Safety concerns  3. Efficacy Concerns  4. Inconvenience |
| Suhadev et al., 2006 | 1. Altruism  2. Personal Health Benefits | 1. Lack of Clarity  2. Efficacy Concerns  3. Safety Concerns  4. Stigmatization  5. Placebo concerns |
| Tharawan et al., 2001 | 1.Personal Health Benefits  2. Altruism  3. Access to Health Care  4. Trust  5. Social Support  6. Convenience  7. Monetary Benefit | 1. Lack of Social Support  2. Safety Concerns  3. Placebo concerns  4. Distrust  5. Lifestyle effect  6. Physical Pain  7. Efficacy concerns  8. No perceived need  9. Stigmatization |
| Thienkrua et al., 2014 | 1. Altruism  2. Access to Health Care  3. Personal Health Benefits  4. Knowledge  5. Convenience  6. Perception of risk  7. Trust  8. Monetary Benefit | 1. Placebo concern  2. Invasive Procedures  3. Inconvenience  4. Physical Pain  5. Lack of Social Support  6. Fear of Health status |
| Udrea et al., 2009 | 1. Personal benefit  2. Altruism  3. Access to Health Care  4. Monetary Benefit | 1. Safety Concerns  2. Placebo concerns  3. Non-compliance to terms of research  4. No perceived need  5. Lack of Knowledge  6. Physical Pain  7. Distrust  8. Inconvenience |
| Vieira de Souza et al., 2003 | 1. Altruism  2. Personal Health Benefits  3. Trust | 1. Lack of Knowledge  2. Safety concerns  3. Distrust |
| Woodsong et al., 2012 | 1. Personal Health Benefit  2. Altruism  3. Access to Health Care |  |
| Wu et al., 2015 | \| 1. Altruism  2. Personal Health Benefit  3. Access to health care  4. Monetary Benefit \| \| --- \| | 1. Safety Concerns  2. Personal costs  3. Distrust  4. Confidentiality concerns  5. Inconvenience |
| Ye et al., 2011 |  | 1. Safety Concerns  2. No perceived need  3. Efficacy concerns  4. Lack of Social Support  5. Stigmatization |
| Yin et al., 2008 | 1. Altruism  2. Monetary Benefit  3. Knowledge  4. Personal Health Benefit  5. Motivation to Avoid Risky Behavior  6. Social Support | 1. Efficacy Concerns  2. False-positive testing  3. Safety concerns  4. Stigmatization |

Supplementary Table 3 Number of studies in which a reason was mentioned

|  | Studies | No. of studies |  | Studies | No. of studies |
| --- | --- | --- | --- | --- | --- |
| Reasons for Participation | | | **Reasons for non-participation** | | |
| Ability to withdraw | ^92^ | 1 | Confidentiality concerns | ^92,20–22,43,108,87,44,46,58^ | 12 |
| Access to Health Care | ^20,21,58,19,67,24,25,75,27,29,31,32,76,33,34,77,78,38,40,41,3,79,71,80–83,49,50,53–55,57,84,60,62,63,85,86,64,72,73^ | 42 | Costs | ^44,58,69,104,112^ | 5 |
| Altruism | ^20–22,43,44,46,58,19,24,25,27,29,31–34,38,40,41,49,50,53–55,57,60,62–64,23,26,28,30,1,35–37,39,42,45,47,48,51,52,56,59,61^ | 46 | Cultural insensitivity | ^37,102^ | 2 |
| Advice from physician | ^20,27,77,86^ | 4 | Distrust | ^21,58,34,41,83,53,55,62,37,39,42,45,56^ | 14 |
| Community involvement | ^34,61–63,66^ | 5 | Efficacy concerns | ^77,40,53,36,45,47,51,52,59,93,98,99^ | 12 |
| Convenience | ^34,53,54^ | 3 | False-positive test results | ^87,44,36,45,59,94,95,111^ | 8 |
| Cultural acceptability | ^63,92^ | 2 | Lack of social support | ^44,75,32,76,77,41,53,54,85,86,72,48,99,94,95,111,109,68,103,89,106,110,90^ | 23 |
| Feeling of community | ^44^ | 1 | Fear of health status | ^19,54,28,30,68^ | 5 |
| Personal Health benefits | ^22,43,44,46,58,67,25,27,31,33,40,41,71,50,53,54,57,60,62–64,72,73,69,26,28,30,36,39,42,47,48,51,52,56,59,68,70,74^ | 40 | Inconvenience | ^20,28,30–32,34,37,42,44,46,50,51,54,58,67,69,71,101–107^ | 25 |
| Knowledge | ^87,44,67,25,27,76,41,81,82,54,60,62,73,59,89,88^ | 16 | Insufficient compensation | ^21,43^ | 2 |
| Monetary benefit | ^20–22,87,58,19,24,27,31–33,41,3,71,81–83,49,50,53–55,60,86,69,23,35,37,45,48,59^ | 31 | Invasive procedures | ^1,20,30,39,54,78,88,107^ | 8 |
| Low pressure decision | ^75^ | 1 | Lack of interest | ^30,34,37,61,87,96,103,105,106,110^ | 10 |
| Need for treatment | ^77^ | 1 | Lack of Clarity | ^92,1,37,51,52^ ^112,30^ | 7 |
| Non-invasive procedure | ^46,81^ | 2 | Non-compliance to terms of research | ^55,68,94,107,109^ | 5 |
| Peer Enrolment | ^44,33,85^ | 4 | No perceived need | ^29,53,55,26,99^ | 5 |
| Low Perception of risk | ^54,70,74^ | 2 | Overwhelmed | ^110^ | 1 |
| Personal benefit | ^34,41,81,55,121^ | 5 | Physical pain | ^20,44,19,31,76,83,50,53–55,28,47,106^ | 13 |
| Result availability | ^92^ | 1 | Placebo concerns | ^29,40,52–55^ | 6 |
| Social support | ^20,22,75,32,78,53,63,85,86,69,23,35,36,42,59,89–91^ | 18 | Previous negative experience | ^75,112^ | 2 |
| Trust | ^78,53–55,60,62,63,86,72,73,69,30,1,56,74,88^ | 17 | Re-contact | ^92^ | 1 |
| Guarantee of Confidentiality | ^33,34,62,23^ | 4 | Safety concerns | ^20–22,58,67,31,32,76,34,77,40,41,71,50,53,55,62,69,23,26,28,30,1,36,37,39,42,45,47,48,51,52,56,59,61,93,98,99,94,95,68,89,96,97,100^ | 45 |
| Unaware of voluntariness | ^46^ | 1 | Stigmatisation | ^22,108,87,75,31,32,41,50,53,69,23,36,37,42,47,52,59,99,94,95,68^ | 20 |
| Motivation to avoid risky behavior | ^59^ | 1 | Temptation to unsafe behavior | ^43^ | 1 |
|  |  |  | Lack of Perceived Benefit | ^93^ | 1 |
|  |  |  | Effect on lifestyle | ^20,53^ | 2 |
| Research Outcomes | ^60,62,66,85^ | 4 | Worsening of Medical condition | ^100,101,103^ | 3 |
|  |  |  | Lack of Knowledge | ^55,26,56,102,89,96^ ^75^ | 7 |
|  |  |  | Misconception |  | 1 |

Supplementary Table 4 Overall Ranking of Reasons per Region

|  | Sub-Saharan Africa | | Asia | South and Latin America | North Africa and the Middle East | Eastern Europe |
| --- | --- | --- | --- | --- | --- | --- |
|  | **Top Reasons for Participation** | | | | | |
| 1 | Altruism | Altruism | | Altruism | Altruism | Personal benefit |
| 2 | Personal Health Benefits | Personal Health Benefits | | Personal Health Benefits | Cultural Acceptability | Access to Health Care |
| 3 | Access to Health Care | Access to Health Care | | Access to Health Care | Social Support | Altruism |
| 4 | Monetary Benefit | Monetary Benefit | | Trust | Trust | Monetary Benefit |
| 5 | Knowledge | Social Support | | Monetary Benefit | Advice from Physician | Knowledge |
| 6 | Social Support | Knowledge | | Social Support | Ability to Withdraw | Personal Health Benefit |
| 7 | Trust | Trust | |  | Result Availability | Trust |
| 8 |  |  | |  | Access to Health Care |  |
| 9 |  |  | |  | Monetary Benefit |  |
|  | **Top Reasons for Non-Participation** | | | | | |
| 1 | Safety Concerns | Safety Concerns | | Safety Concerns | Invasive Procedures | Safety Concerns |
| 2 | Stigmatization | Stigmatization | | Invasive Procedures | Safety Concerns | Placebo concerns |
| 3 | Inconvenience | Lack of Social Support | | Worsening of medical condition | Confidentiality Concerns | Non-compliance to terms of research |
| 4 | Lack of Social Support | Inconvenience | | Lack of Knowledge | Lack of Clarity | No perceived need |
| 5 | Physical Pain | Efficacy Concerns | | Lack of Social Support | Physical Pain | Lack of Knowledge |
| 6 | Efficacy concerns | Confidentiality Concerns | | Stigmatization | Re-contact | Physical Pain |
| 7 | Confidentiality Concerns | Physical Pain | | False-positive test results | Inconvenience | Distrust |
| 8 | Distrust | Placebo Concerns | | Fear of Health status | Effect on Lifestyle | Inconvenience |

Supplementary Table 5 Overall Ranking of Reasons - HIV vs. Non-HIV Research

|  | HIV Research | |
| --- | --- | --- |
|  | **Top Reasons for Participation** | **Top Reasons for Non-Participation** |
| 1 | Altruism | Safety Concerns |
| 2 | Personal health Benefits | Stigmatization |
| 3 | Monetary Benefit | Lack of Social Support |
| 4 | Access to Health Care | Inconvenience |
| 5 | Knowledge | Efficacy Concerns |
| 6 | Social Support | False-positive Test Results |
| 7 | Trust | Physical Pain |
| 8 | Guarantee of Confidentiality | Lack of Knowledge |
|  | **Non-HIV Research** | |
|  | **Top Reasons for Participation** | **Top Reasons for Non-Participation** |
| 1 | Altruism | Safety Concerns |
| 2 | Access to Health Care | Invasive Procedures |
| 3 | Monetary Benefit | Inconvenience |
| 4 | Personal Benefit | Lack of Social Support |
| 5 | Trust | Physical Pain |
| 6 | Personal Health Benefits | Efficacy Concerns |
| 7 | Social Support | Confidentiality Concerns |
| 8 |  | Worsening of Medical Condition |

Supplementary Table 6 Overall Ranking of Reasons – Non-patient vs. Patient Participants

|  | Non-Patient Participants | |  |
| --- | --- | --- | --- |
|  | **Top Reasons for Participation** | **Top Reasons for Non-Participation** | |
| 1 | Altruism | | Safety Concerns |
| 2 | Personal Health Benefits | | Inconvenience |
| 3 | Access to Health Care | | Stigmatization |
| 4 | Monetary Benefit | | Lack of Social Support |
| 5 | Knowledge | | Efficacy Concerns |
| 6 | Social Support | | Physical Pain |
| 7 | Trust | | Confidentiality Concerns |
| 8 | Guarantee of Confidentiality | | False-Positive Test Results |
|  | **Patient Participants** | |  |
|  | **Top Reasons for Participation** | **Top Reasons for Non-Participation** | |
| 1 | Altruism | | Safety Concerns |
| 2 | Access to Health Care | | Inconvenience |
| 3 | Personal Health Benefits | | Invasive Procedures |
| 4 | Monetary Benefits | | Lack of Social Support |
| 5 | Trust | | Efficacy Concerns |
| 6 | Social Support | | Worsening of Medical Condition |
| 7 | Trust | | Distrust |
| 8 | Non-Invasive Procedure | | Lack of Knowledge |

Supplementary Table 7. Overall Ranking of Reasons - Male vs. Female Participants

|  | Male Participants | |
| --- | --- | --- |
|  | **Top Reasons for Participation** | **Top Reasons for Non-Participation** |
| 1 | Altruism | Safety Concerns |
| 2 | Personal Health Benefits | Inconvenience |
| 3 | Access to Health Care | Physical Pain |
| 4 | Monetary Benefit | Placebo concerns |
| 5 | Knowledge | Stigmatization |
| 6 | Social Support | Efficacy Concerns |
| 7 | Trust | Invasive Procedures |
| 8 | Guarantee of Confidentiality | Confidentiality Concerns |
|  | **Female Participants** | |
|  | **Top Reasons for Participation** | **Top Reasons for Non-Participation** |
| 1 | Personal Health Benefits | Safety Concerns |
| 2 | Altruism | Stigmatization |
| 3 | Access to Health Care | Lack of Social Support |
| 4 | Monetary Benefit | Physical Pain |
| 5 | Non-Invasive Procedure | Costs |
| 6 | Social Support | Lack of Clarity |
| 7 | Trust | Efficacy Concerns |
| 8 | Advice from Physician | Placebo Concerns |

Supplementary Table 8 Overall Ranking of Reasons for Real vs. Hypothetical Studies

|  | Real Studies | |
| --- | --- | --- |
|  | **Top Reasons for Participation** | **Top Reasons for Non-Participation** |
| 1 | Altruism | Incon­­venience |
| 2 | Personal Health Benefits | Lack of Social Support |
| 3 | Access to Health Care | Safety Concerns |
| 4 | Monetary Benefit | Physical Pain |
| 5 | Trust | Worsening of Medical Condition |
| 6 | Social Support | Invasive Procedures |
| 7 | Personal Benefit | Lack of Interest |
| 8 | Advice from Physician | Fear of Health Status |
|  | **Hypothetical Studies** | |
|  | **Top Reasons for Participation** | **Top Reasons for Non-Participation** |
| 1 | Altruism | Safety Concerns |
| 2 | Personal Health Benefits | Inconvenience |
| 3 | Access to Health Care | Stigmatization |
| 4 | Monetary Benefit | Lack of Social Support |
| 5 | Knowledge | Efficacy Concerns |
| 6 | Social Support | Confidentiality Concerns |
| 7 | Trust | Physical Pain |
| 8 | Community Involvement | Distrust |

Figure S1 Overall Ranking of Reasons for Participation (Graph A) and Non-participation (Graph B). Visualization of data given in Table 2.

***Overall***

A
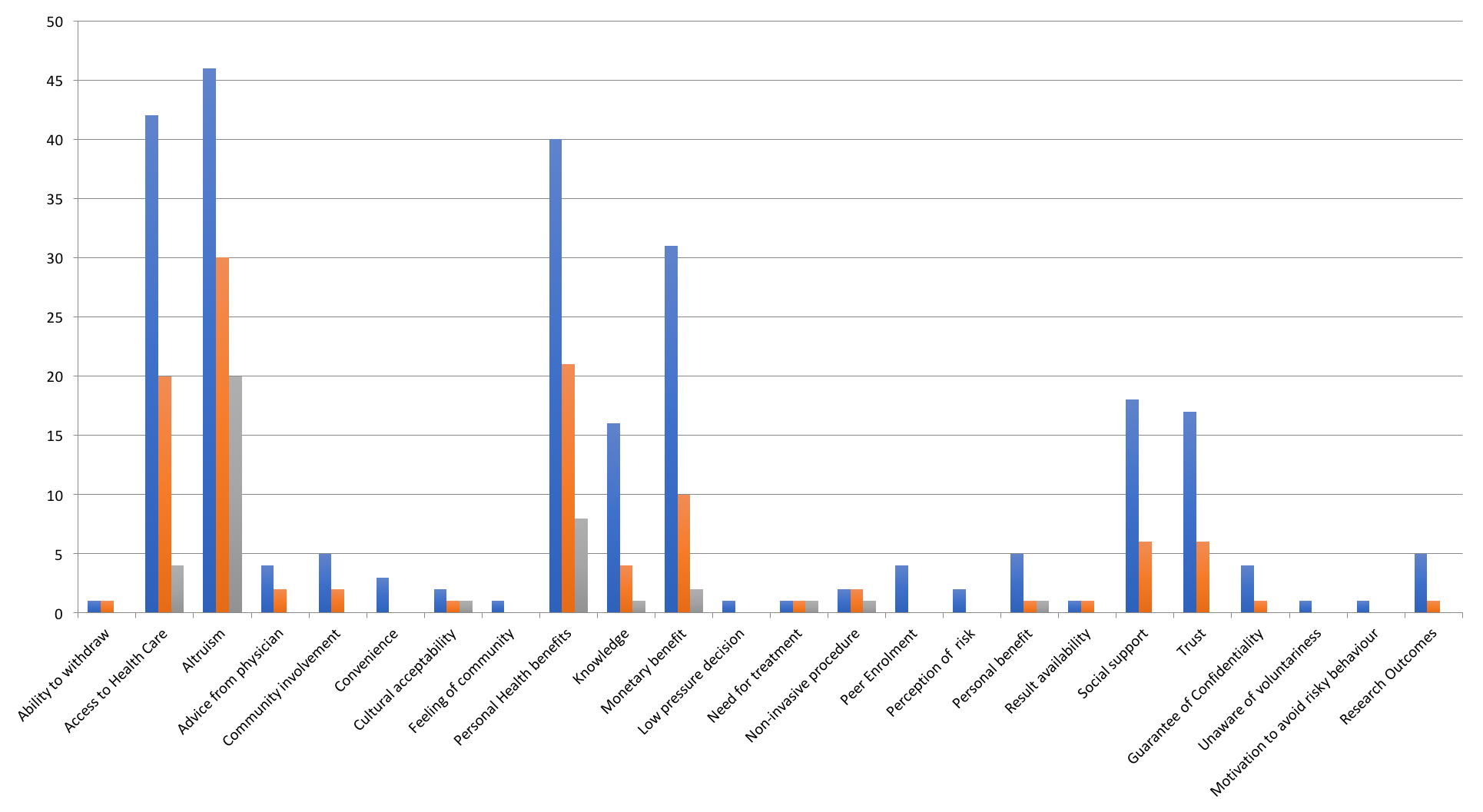


B
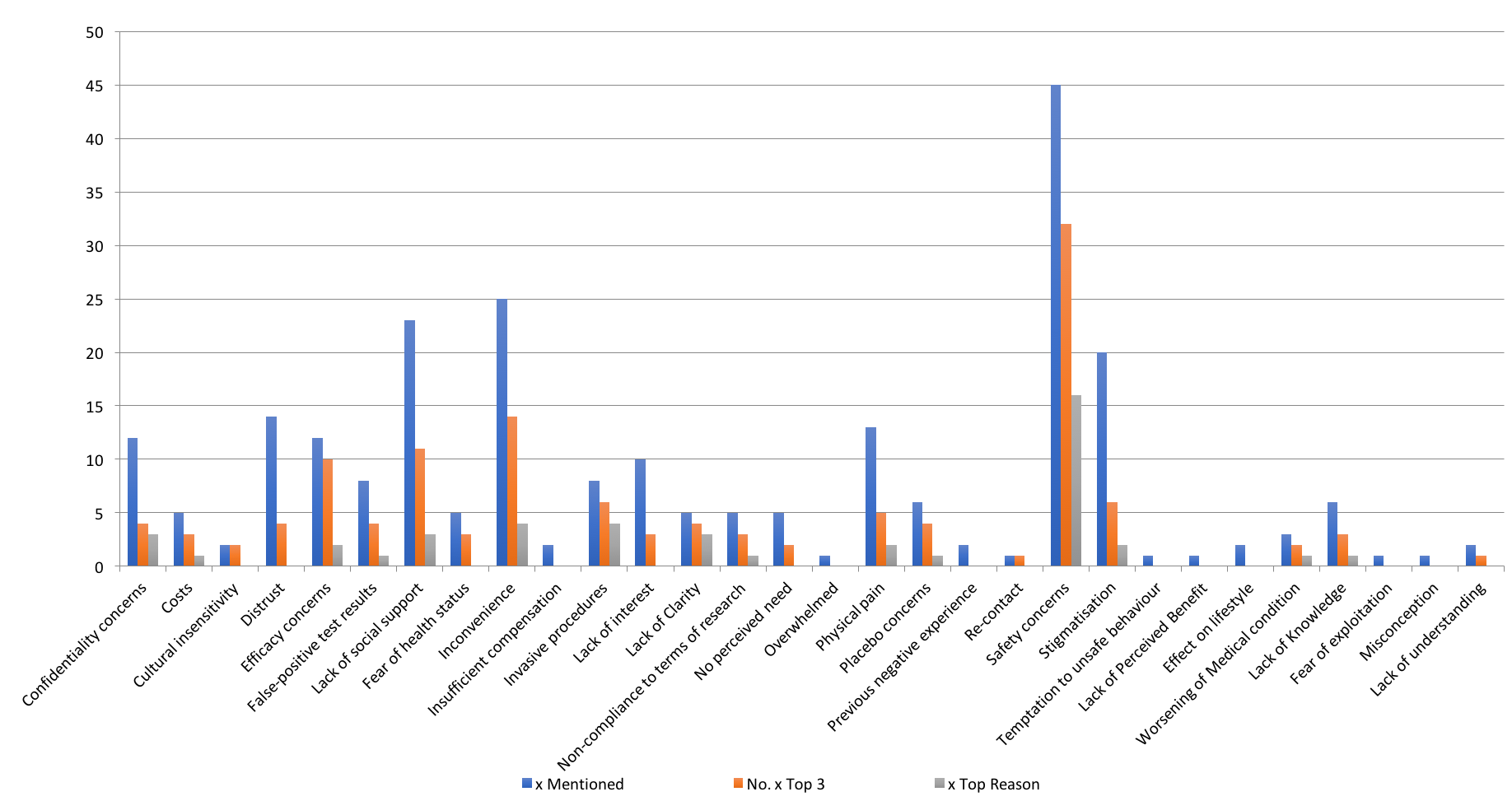


Per Region

A


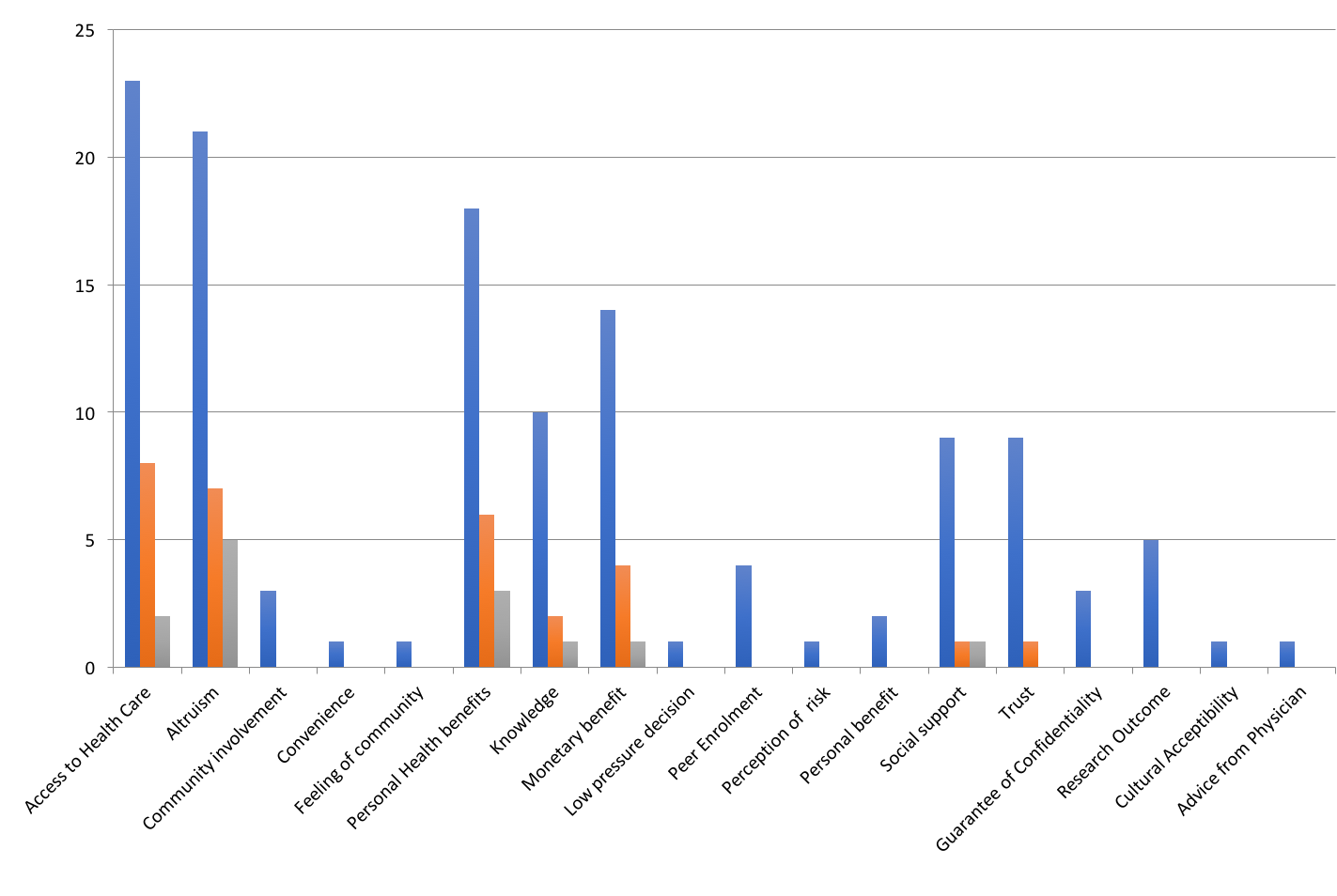


B


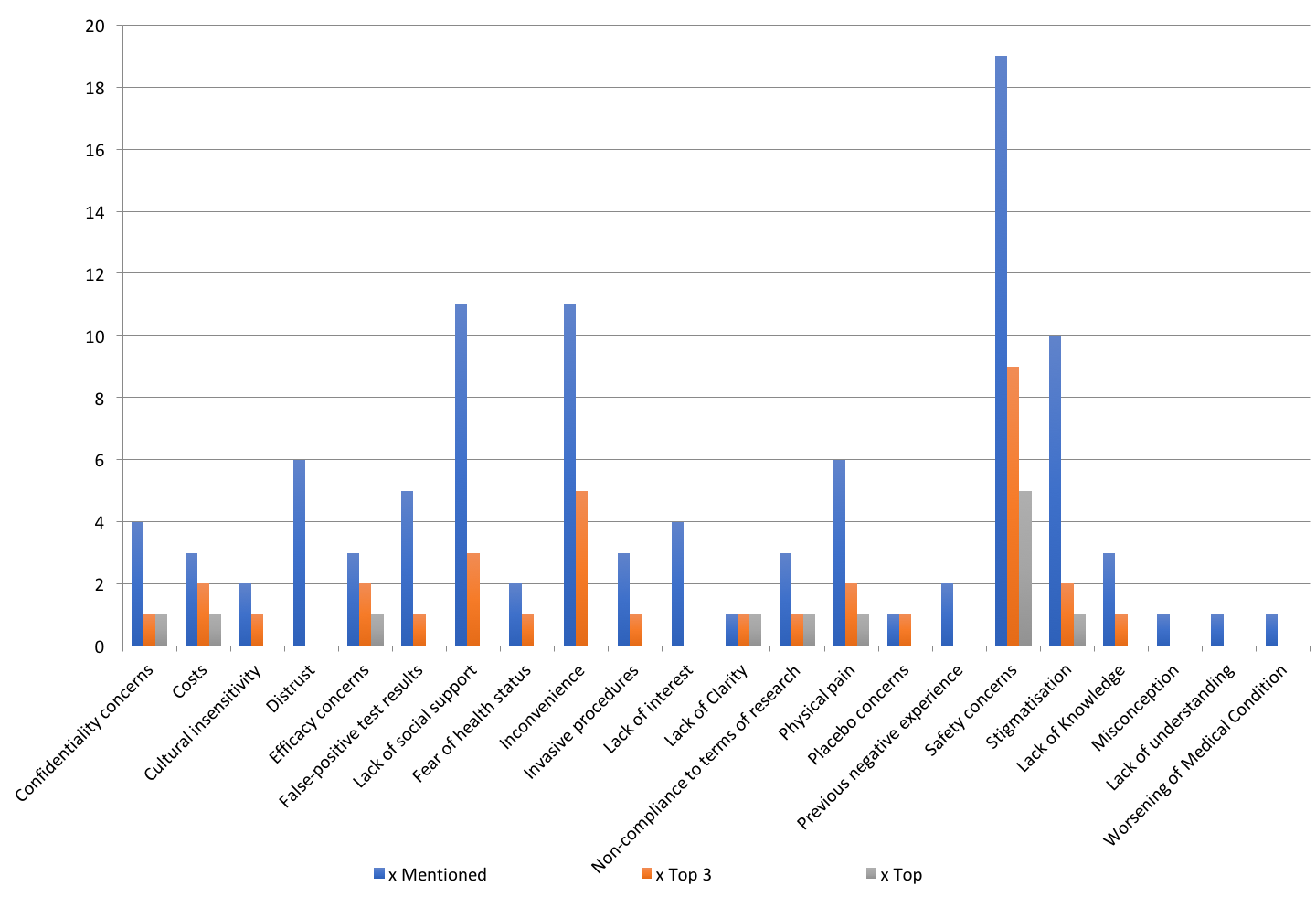


Figure S2 Ranking of Reasons for Participation (Graph A) and Non-Participation (Graph B) - Sub-Saharan Africa

A
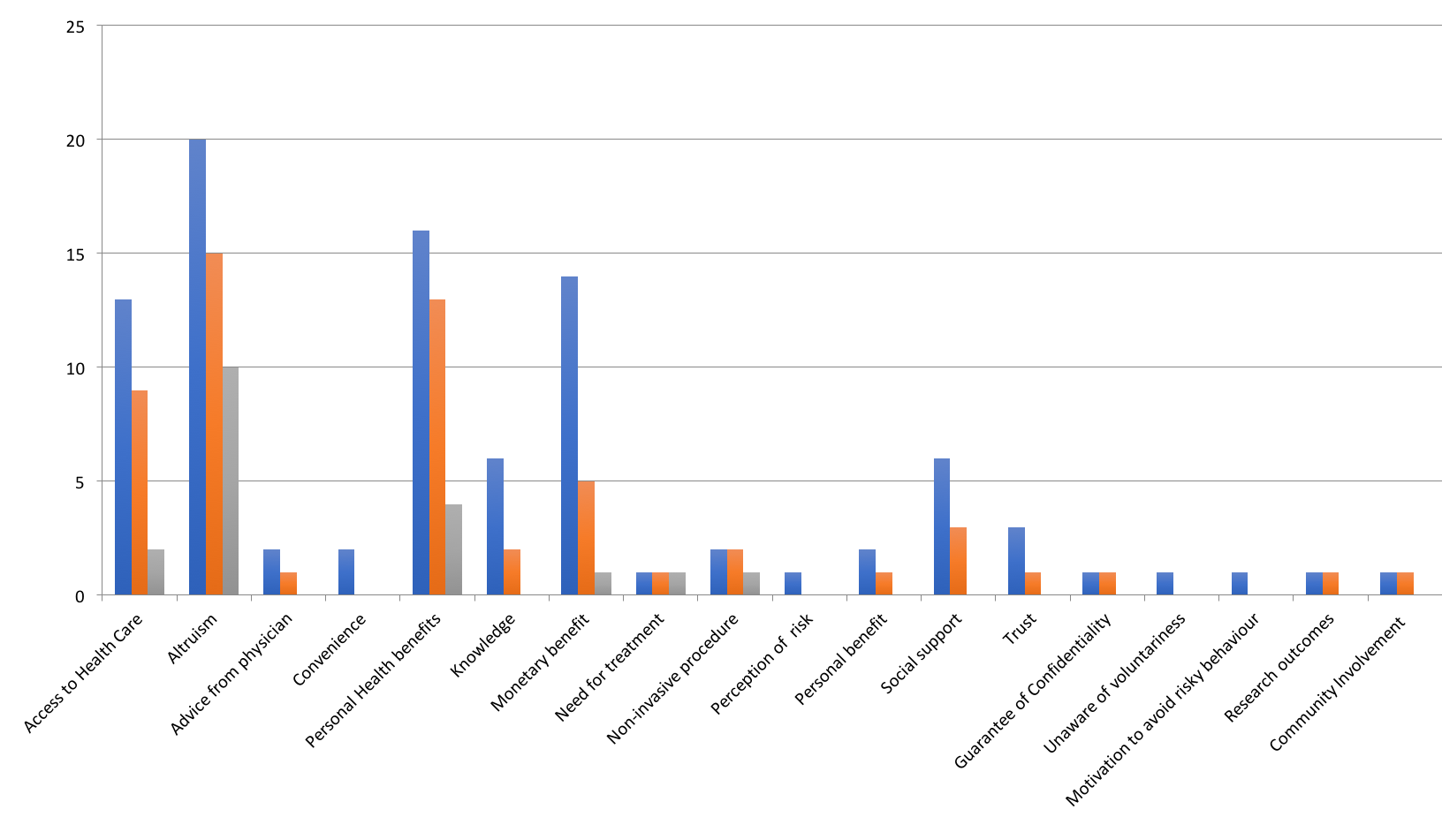


B
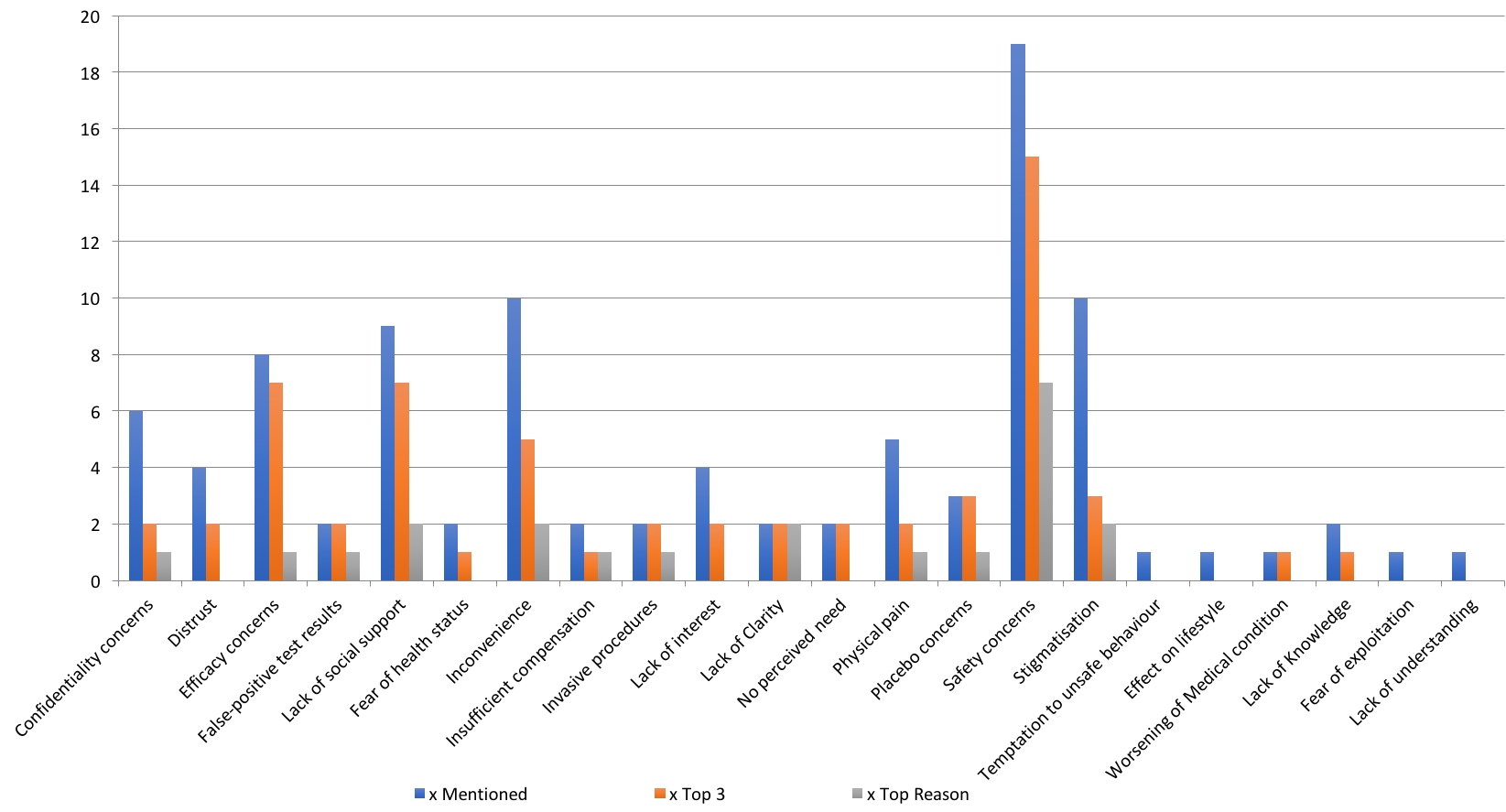


Figure S3 Ranking of Reasons for Participation (Graph A) and Non-Participation (Graph B) – Asia

A
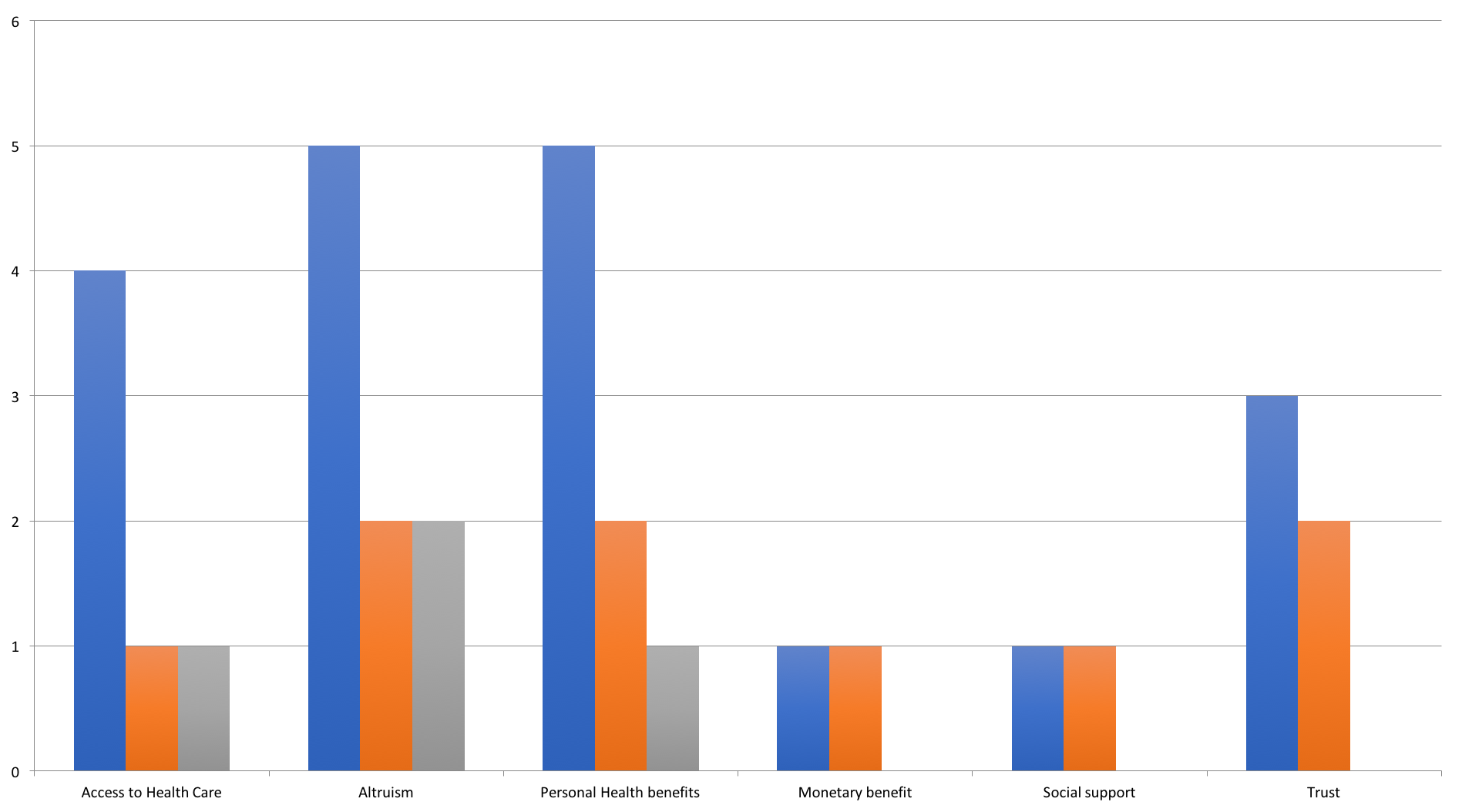


B
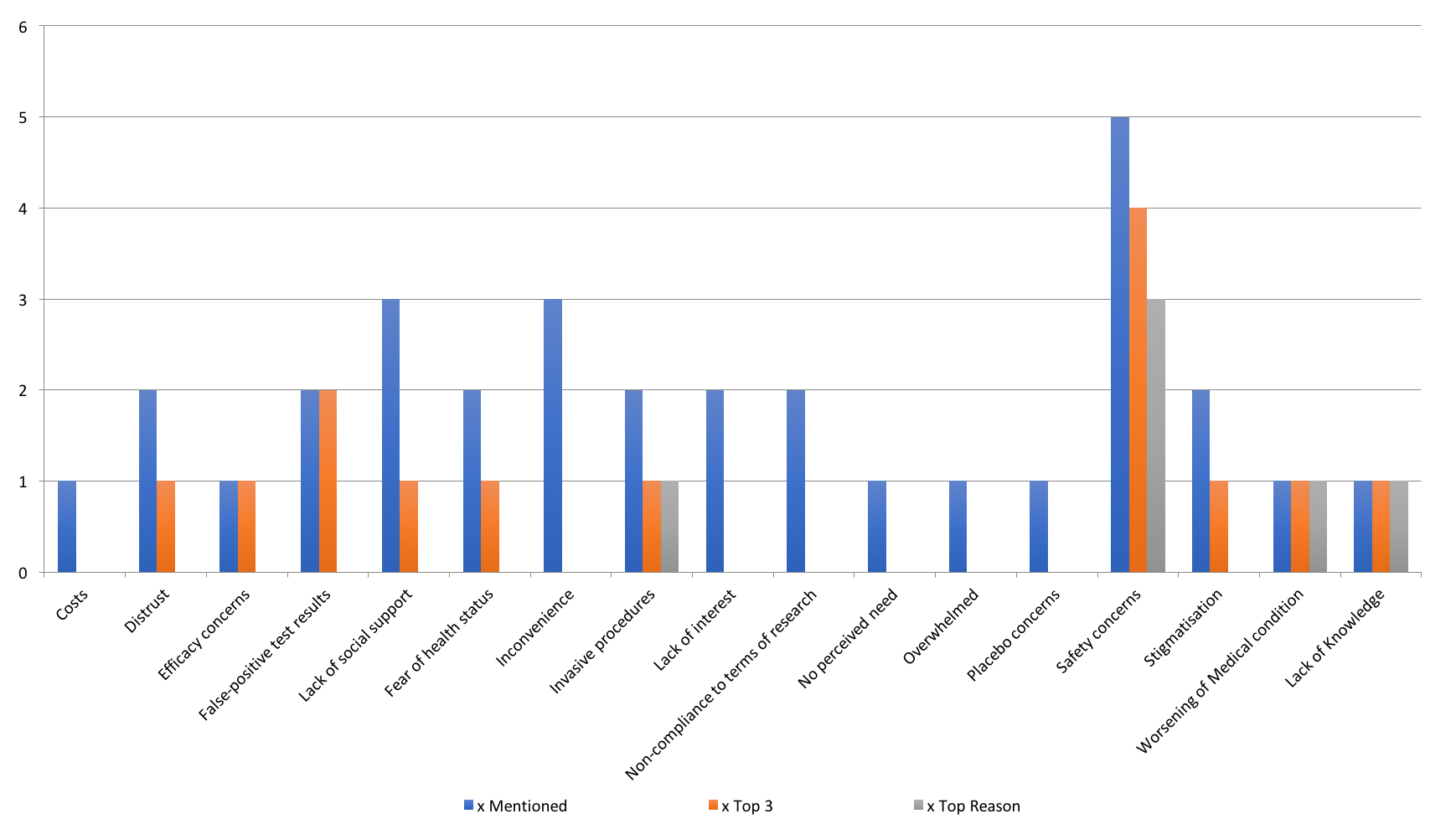


Figure S4 Ranking of Reasons for Participation (Graph A) and Non-Participation (Graph B) – Latin America & the Caribbean

A
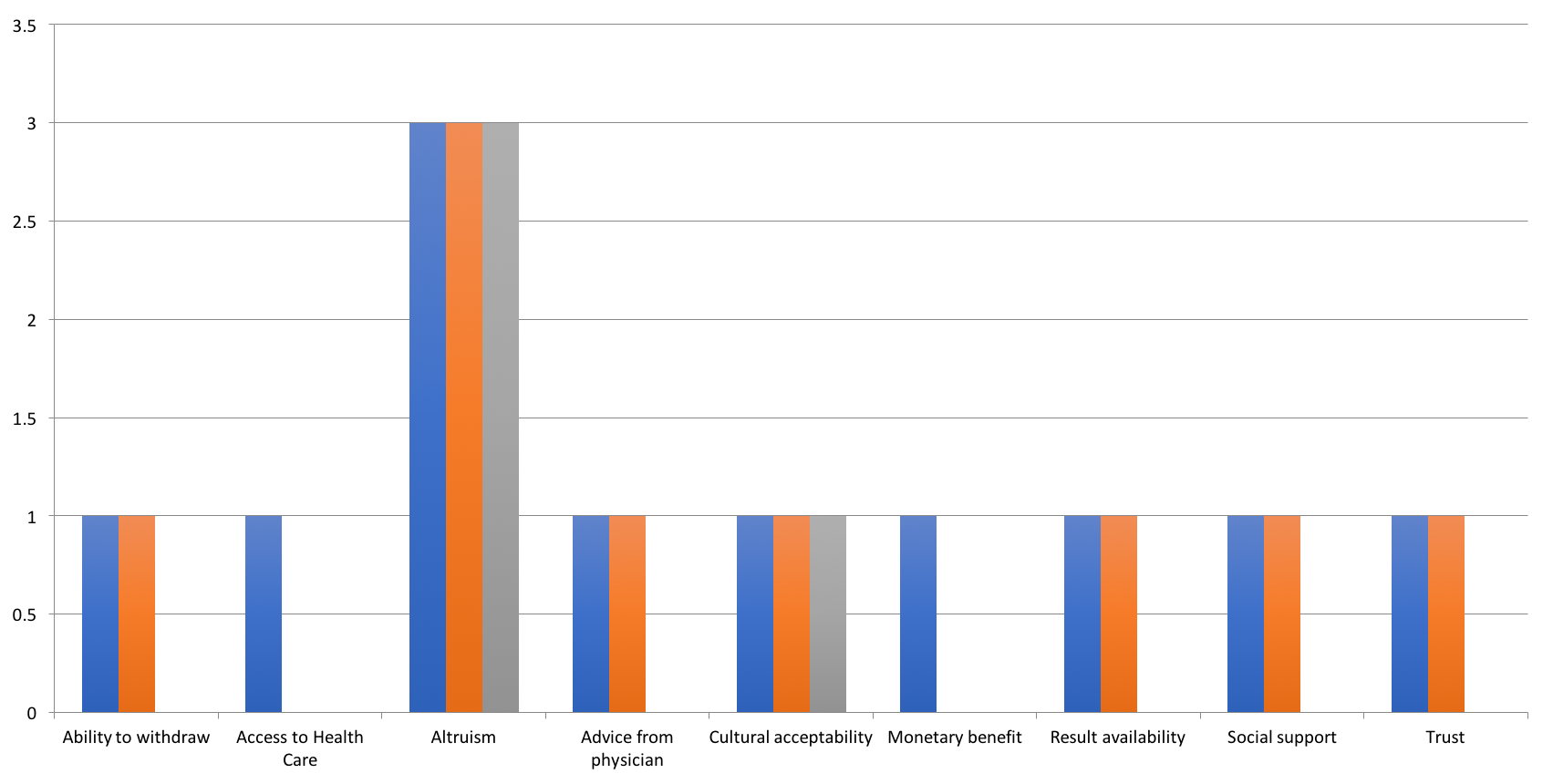


B
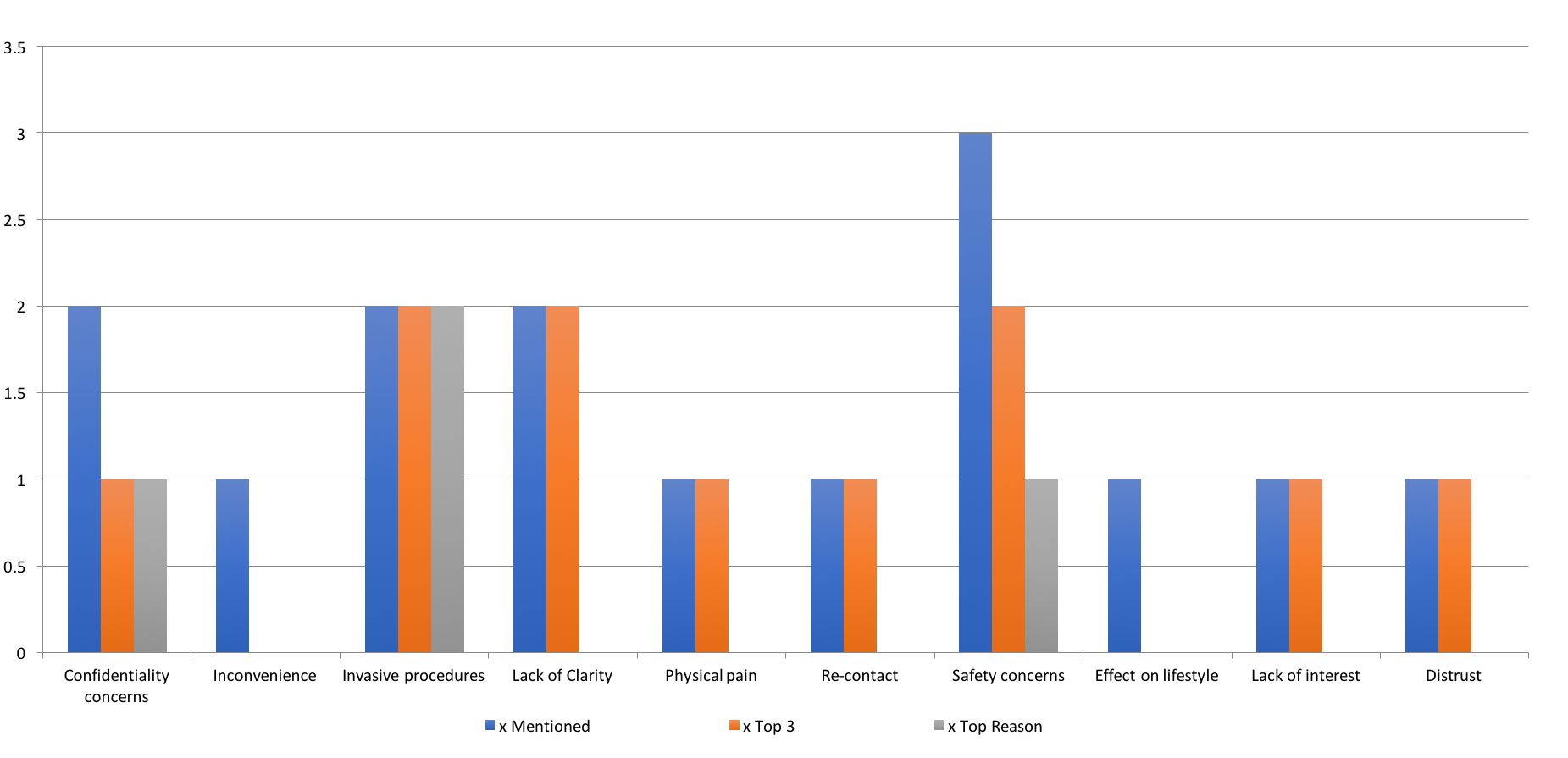


Figure S5 Ranking of Reasons for Participation (Graph A) and Non-Participation (Graph B) – North Africa & the Middle East

*HIV vs. Non-HIV Research*

**A
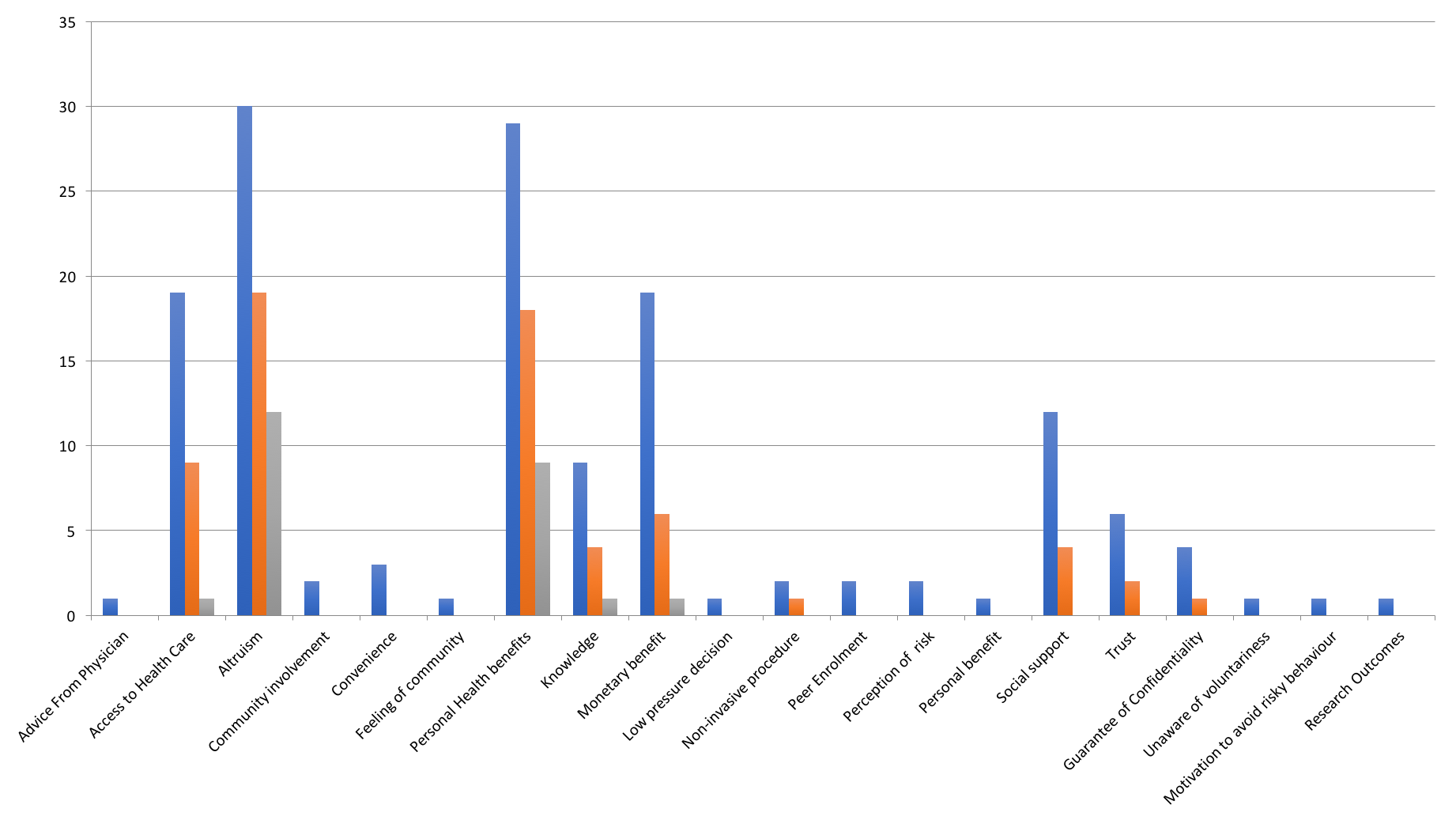
**

**B
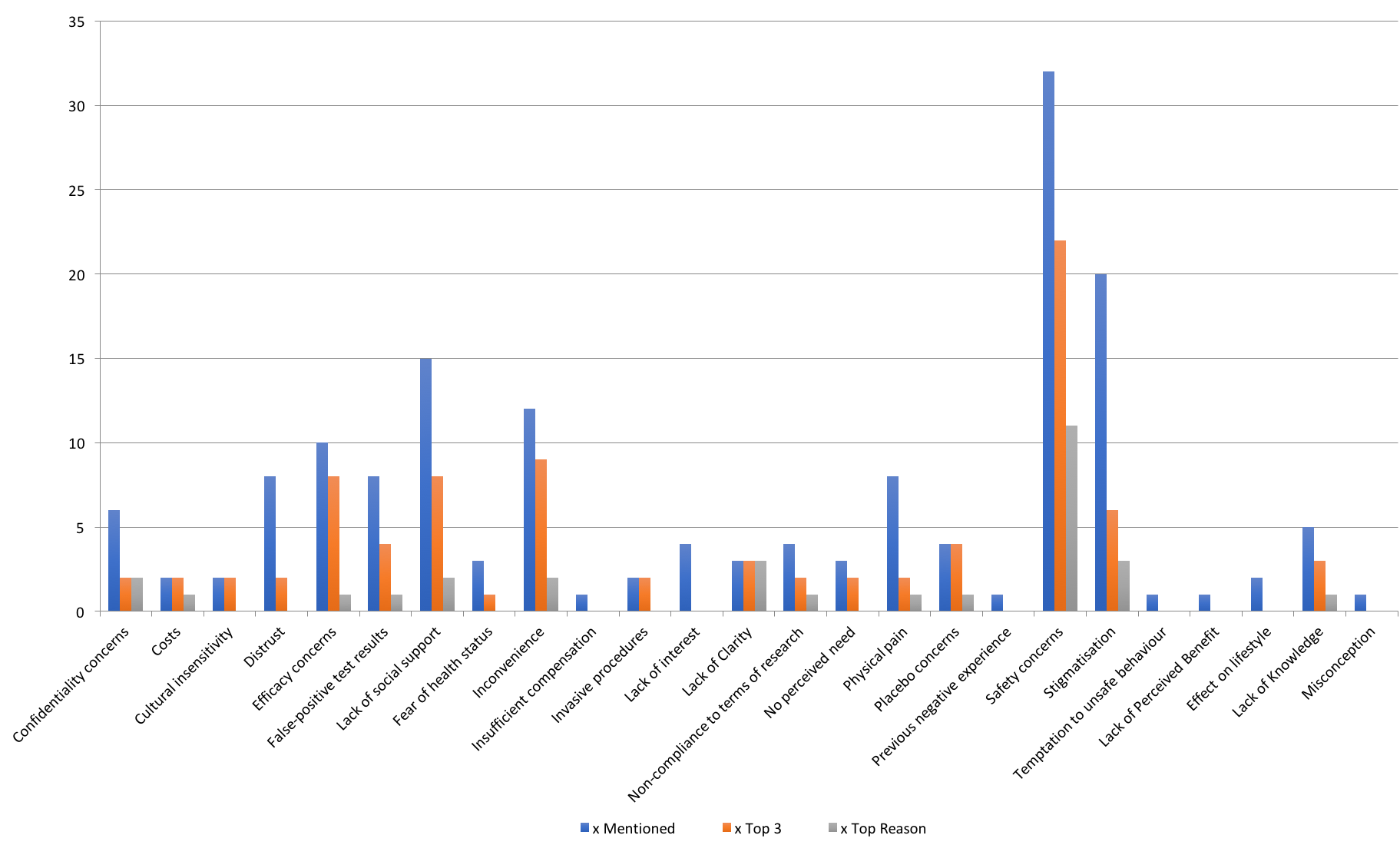
**

Figure S6 Ranking of Reasons for Participation (Graph A) and Non-Participation (Graph B) – HIV Research

A
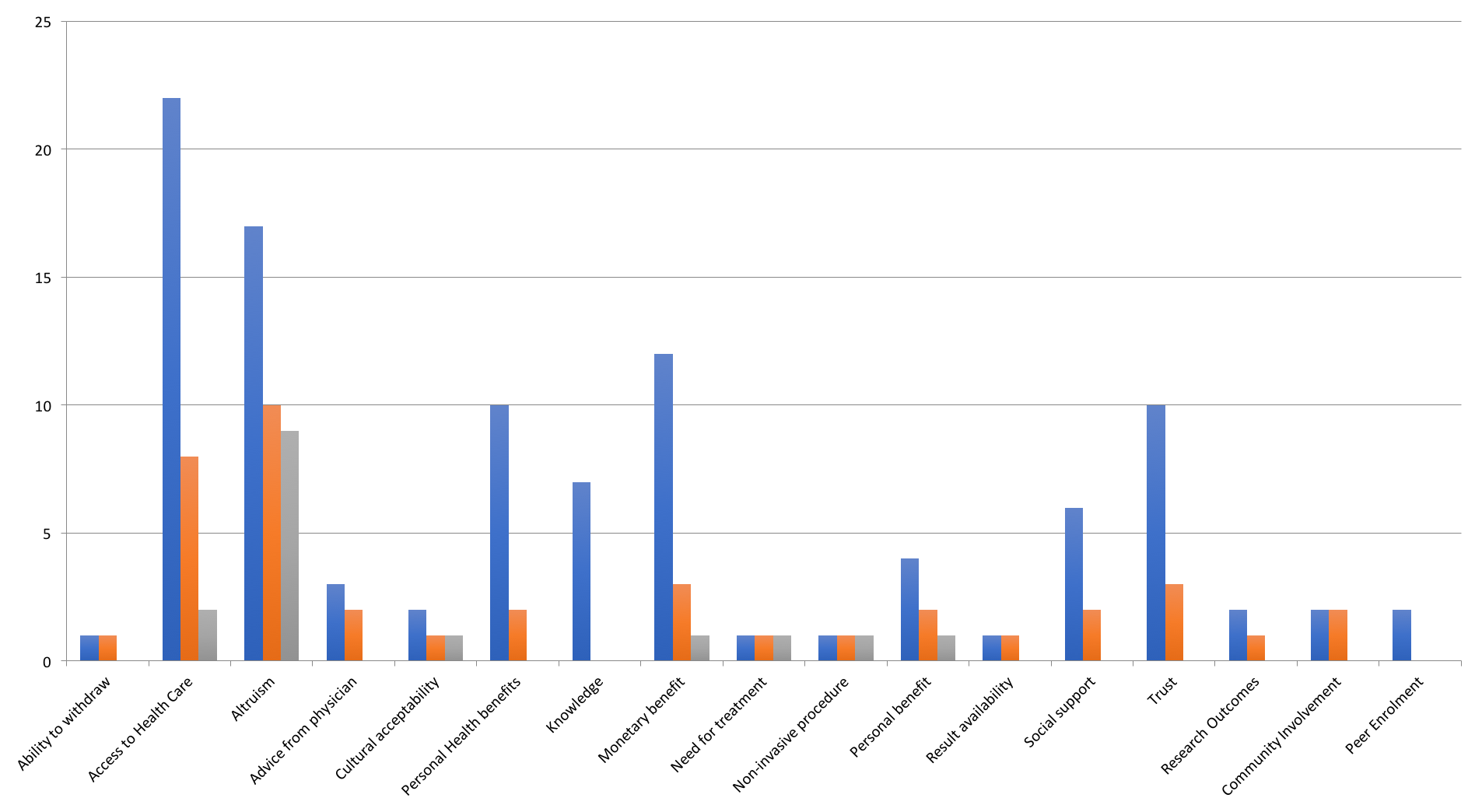


B
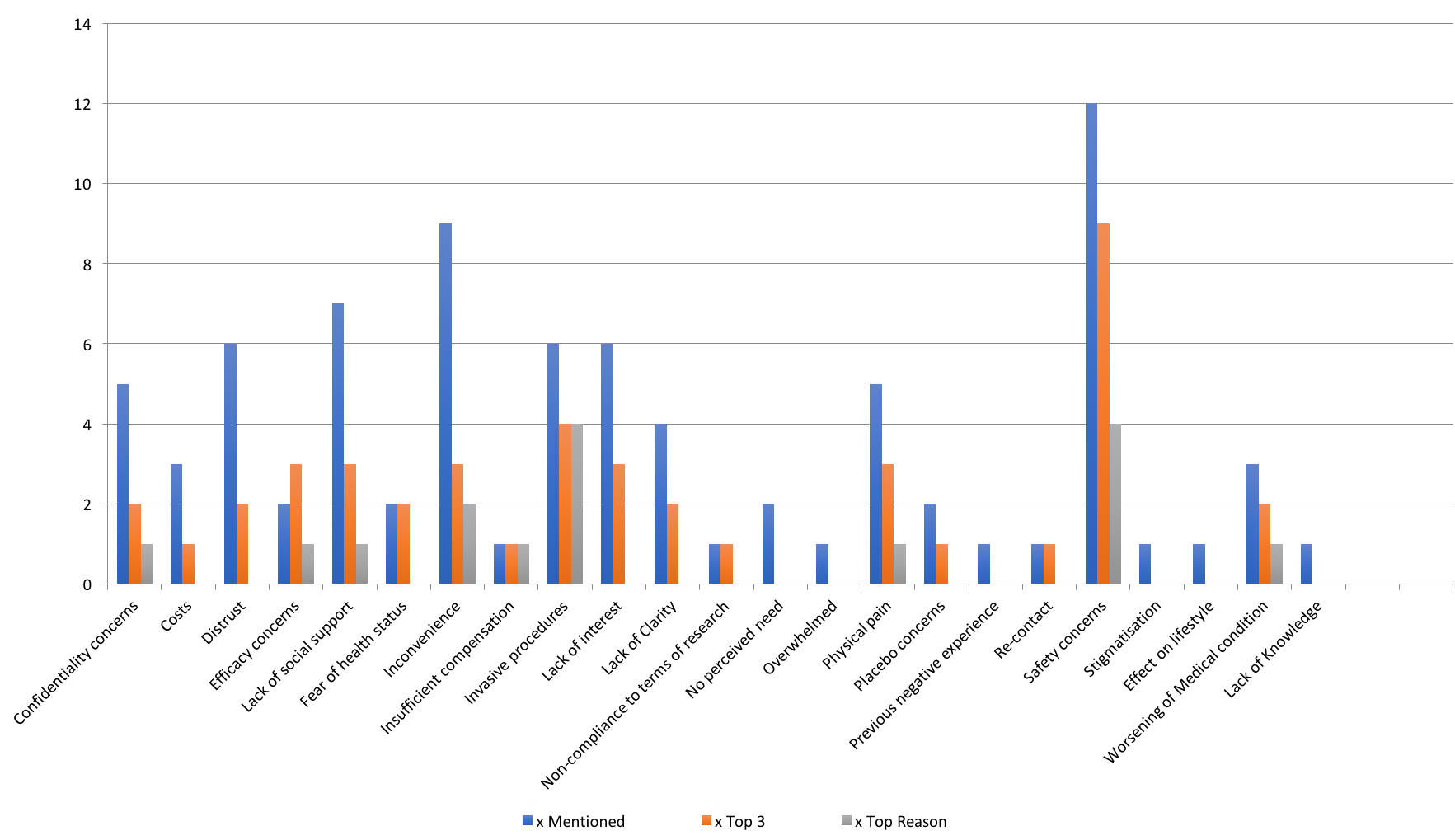


Figure S7 Ranking of Reasons for Participation (Graph A) and Non-Participation (Graph B) – Non- HIV Research

#### Non-Patient vs. Patient Participants

A
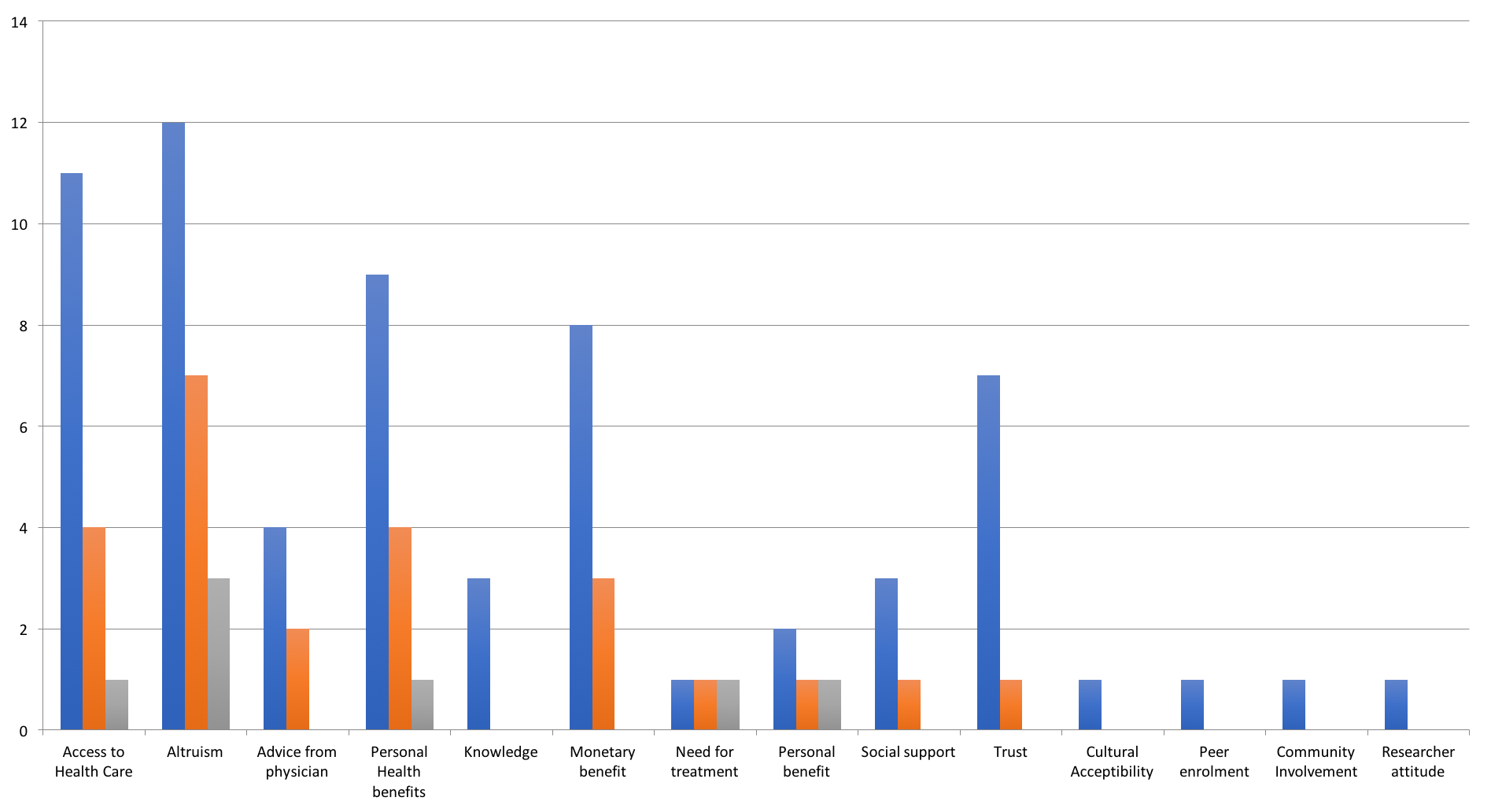


B
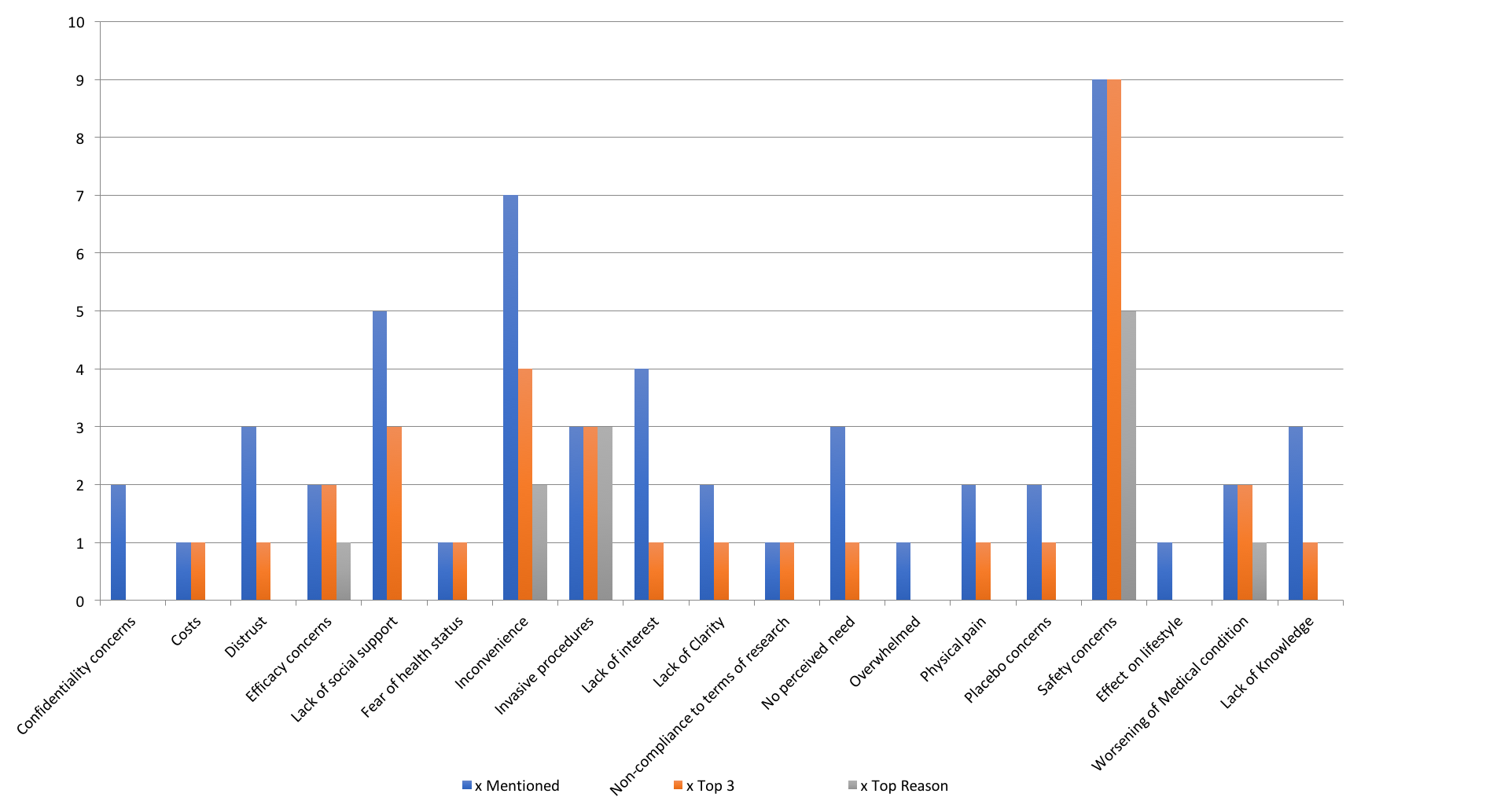


Figure S8 Ranking of Reasons for Participation (Graph A) and Non-Participation (Graph B) – Patients

**A**
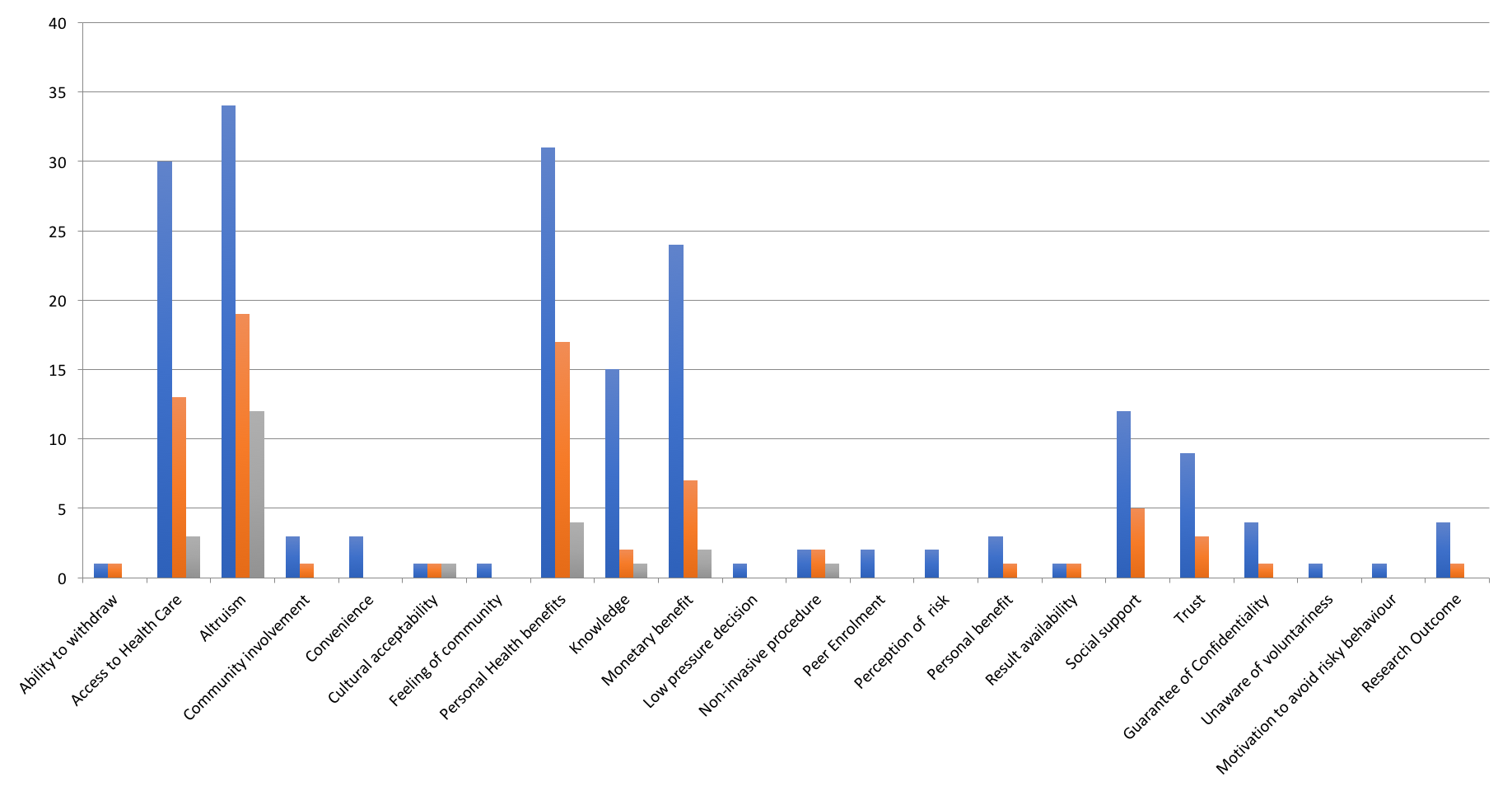


**B**
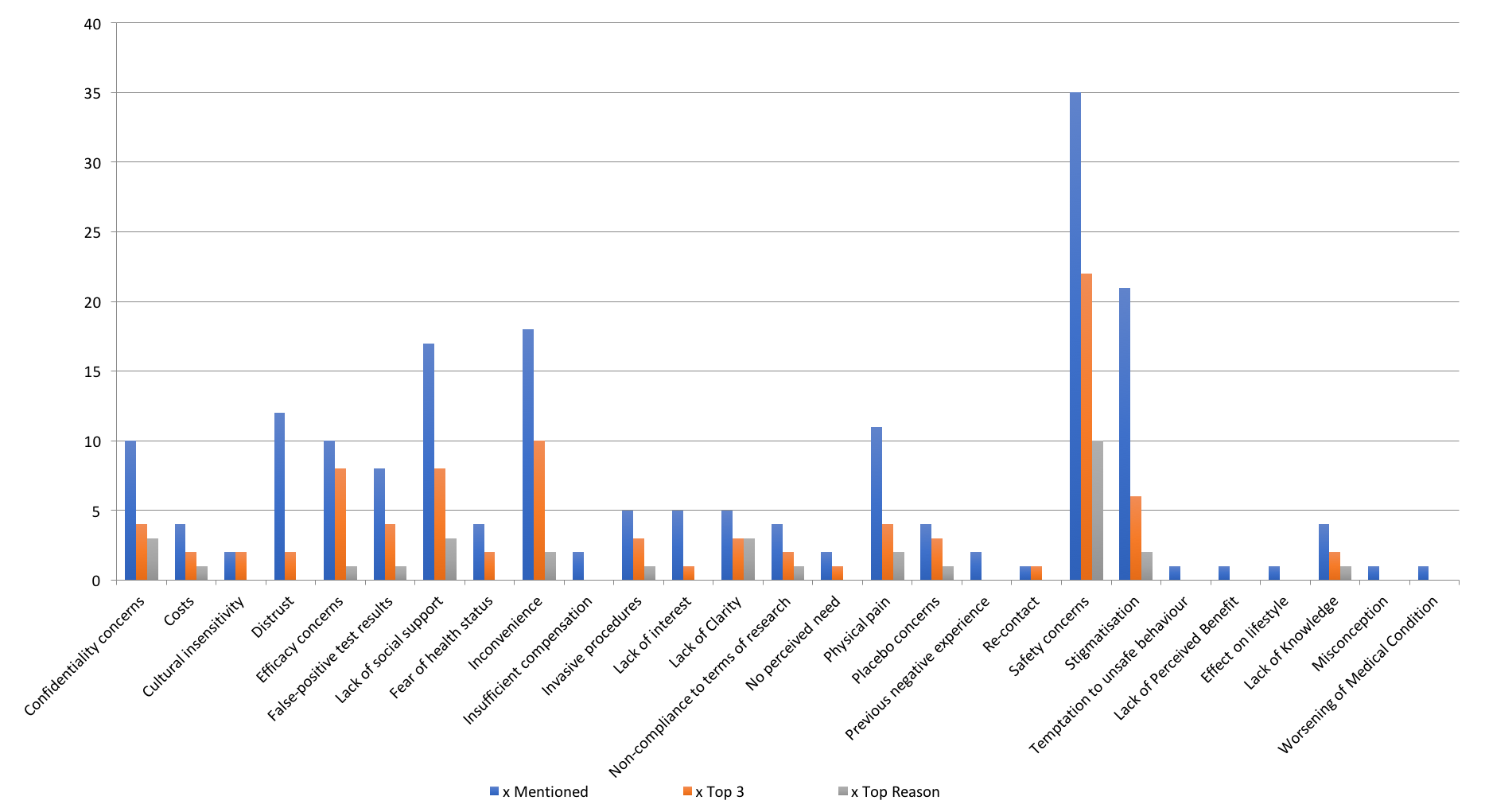


Figure S9 Ranking of Reasons for Participation (Graph A) and Non-Participation (Graph B) – Non-Patients

#### Female vs. Male Participants

A
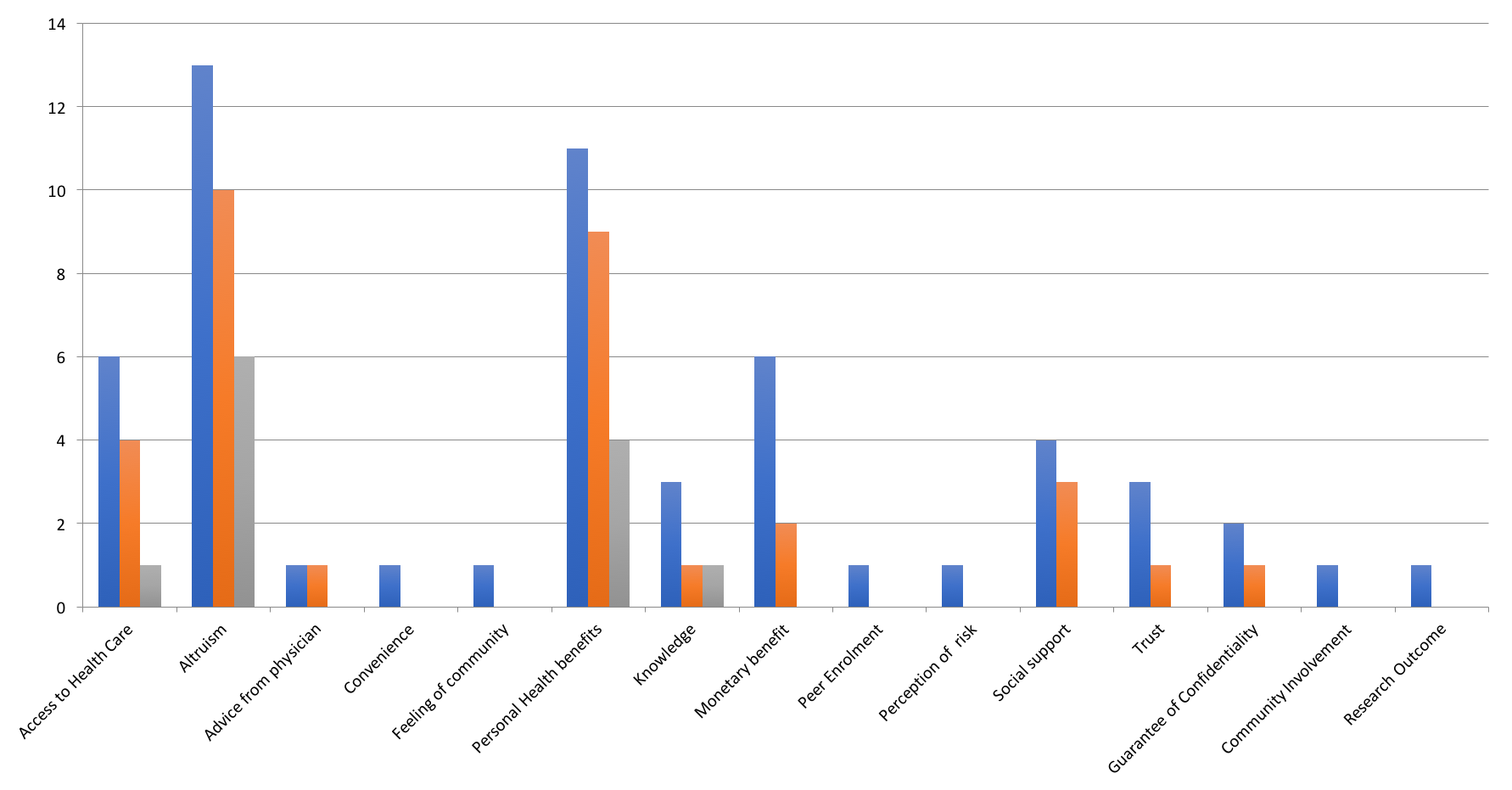


B
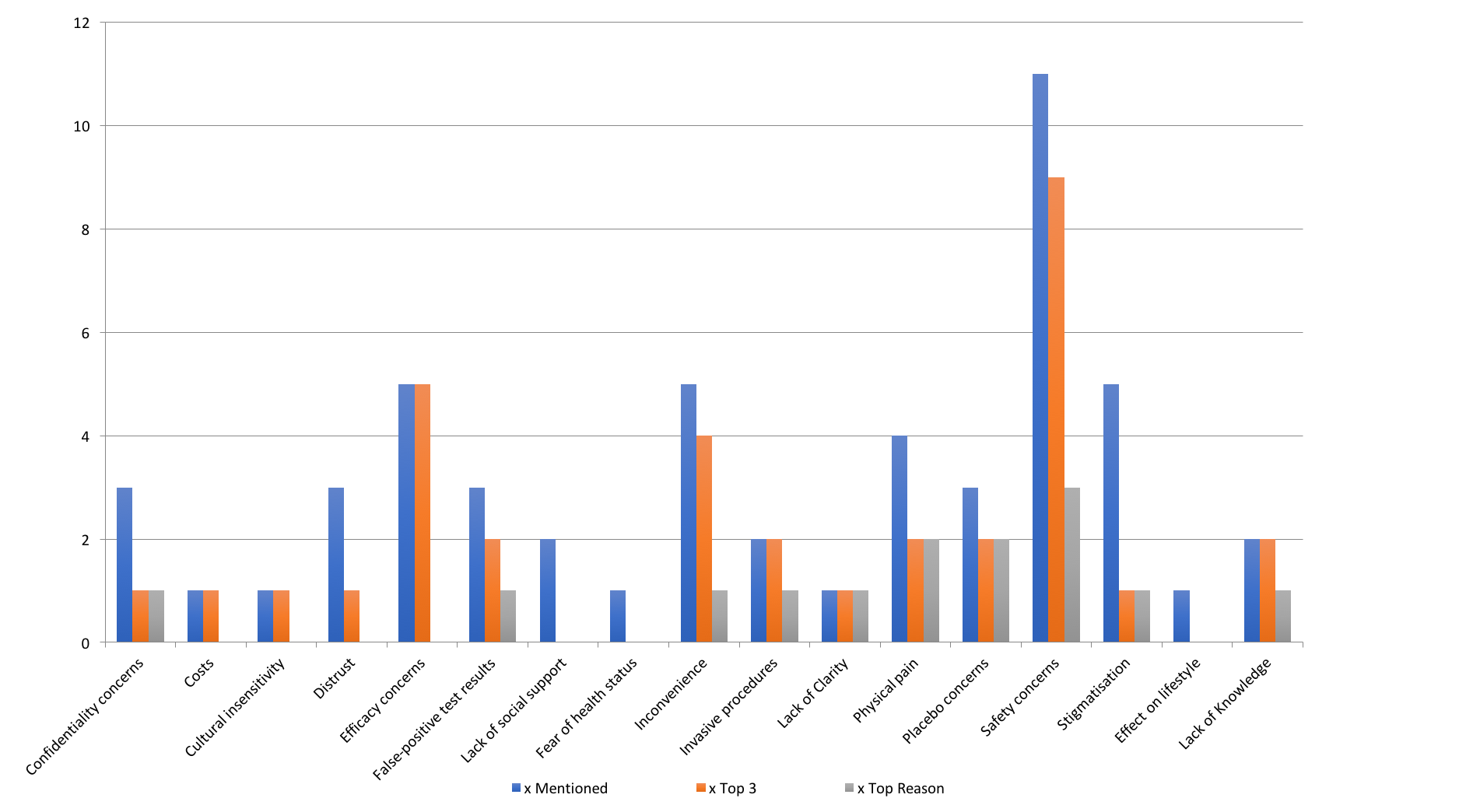


Figure S10 Ranking of Reasons for Participation (Graph A) and Non-Participation (Graph B) – Males

A
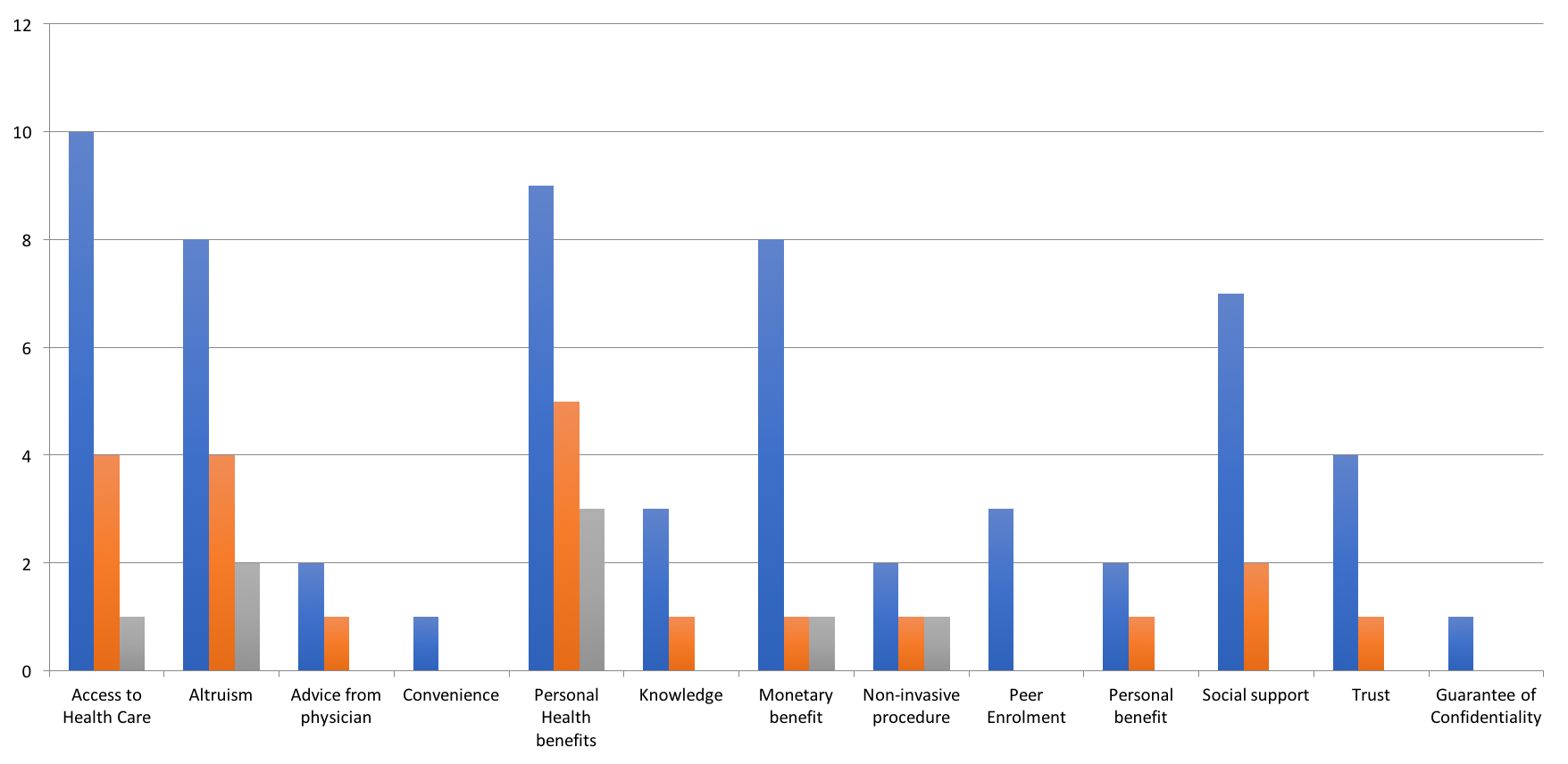


B
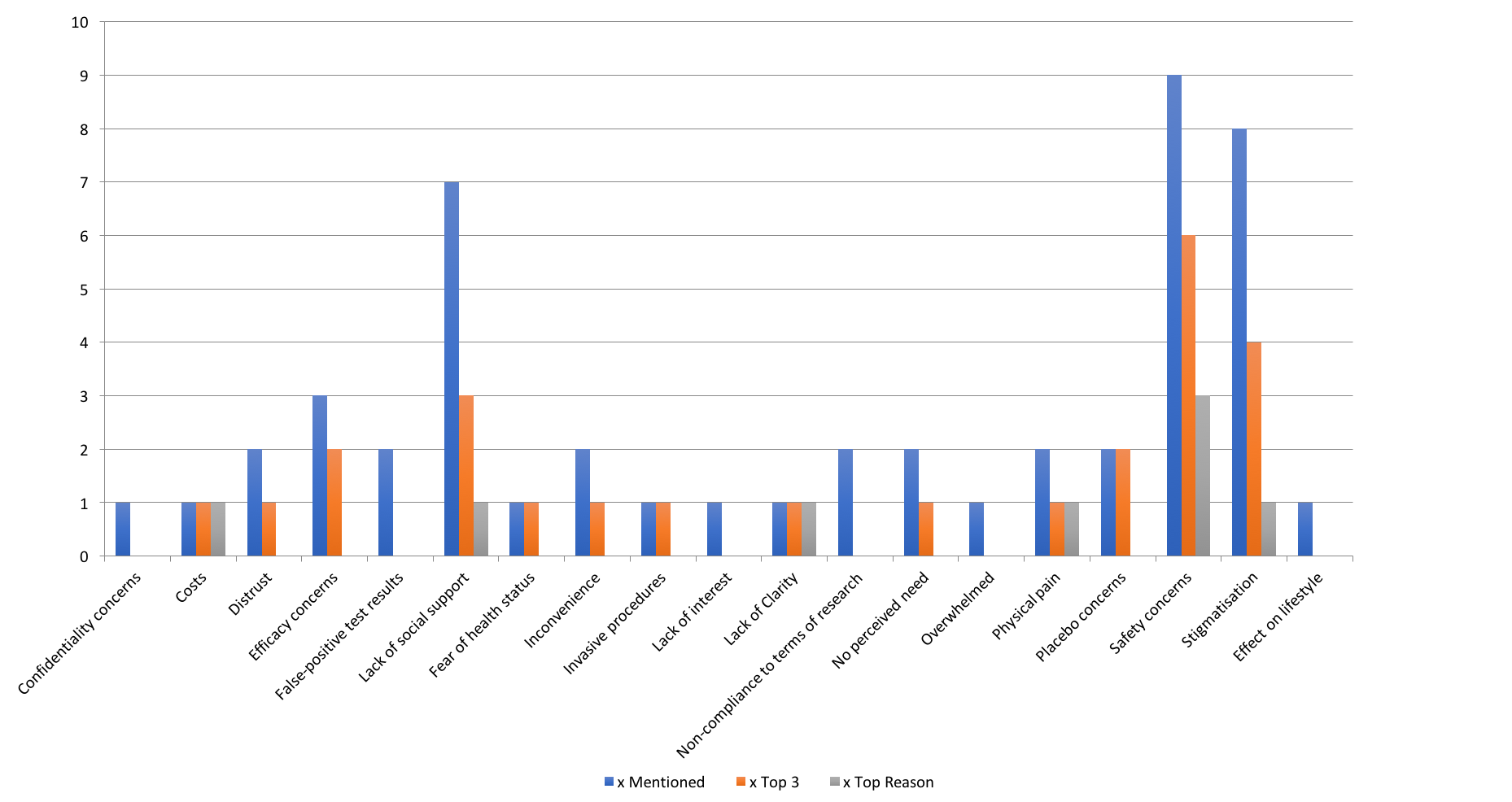


Figure S11 Ranking of Reasons for Participation (Graph A) and Non-Participation (Graph B) – Females

#### Real vs. Hypothetical Studies

A
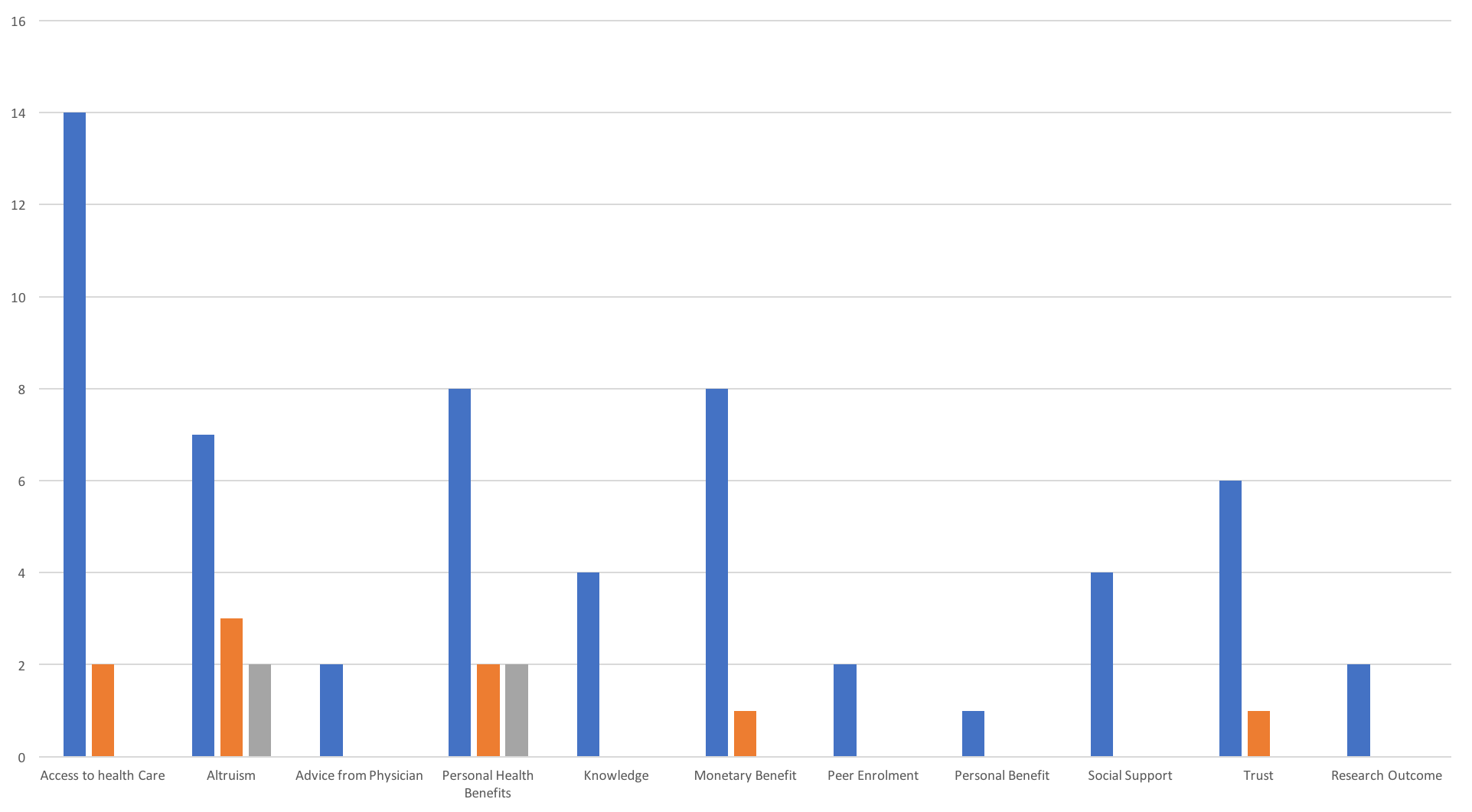


B
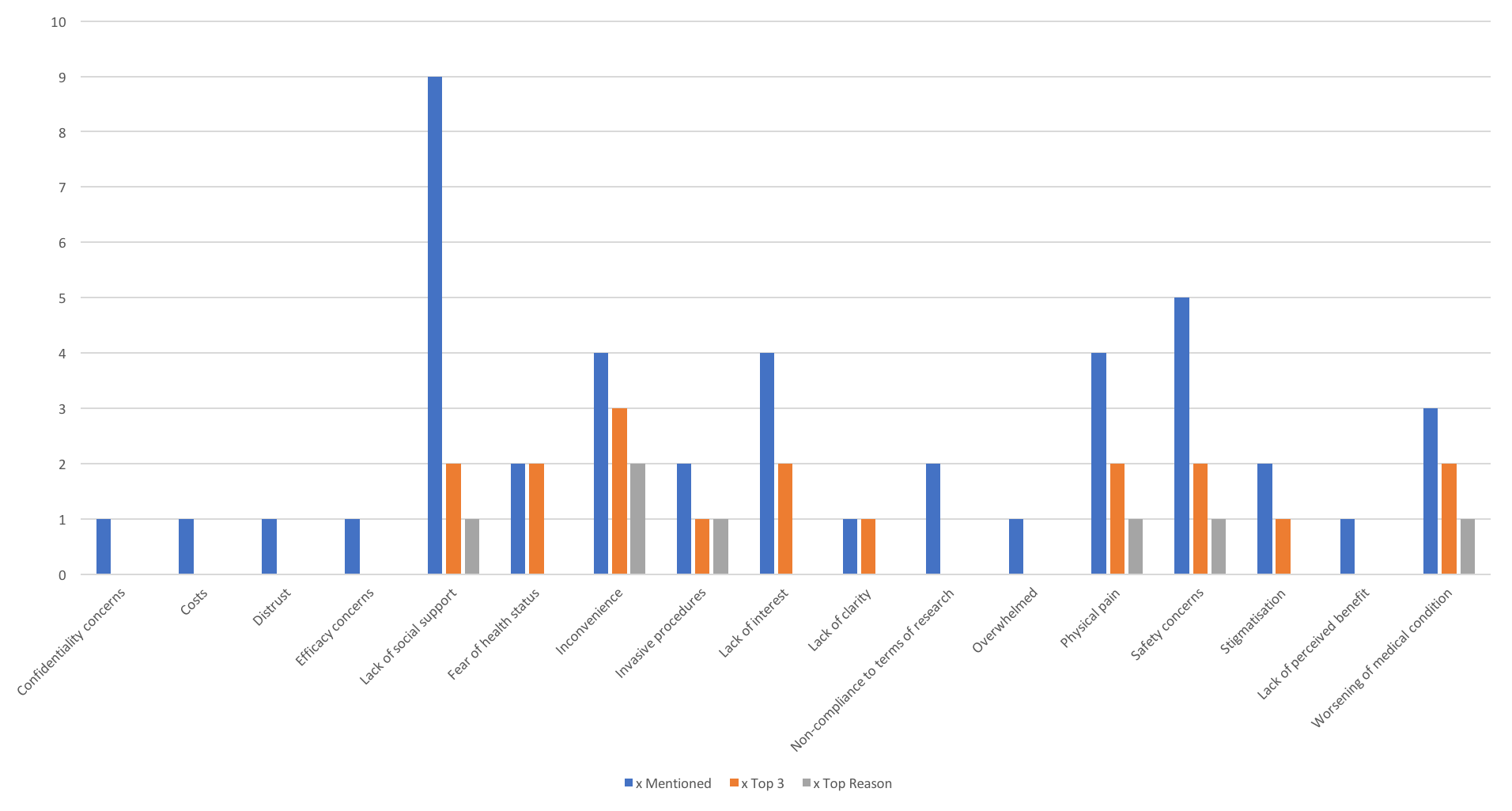


Figure S12 Ranking of Reasons for Participation (Graph A) and Non-Participation (Graph B) – Real Studies

A
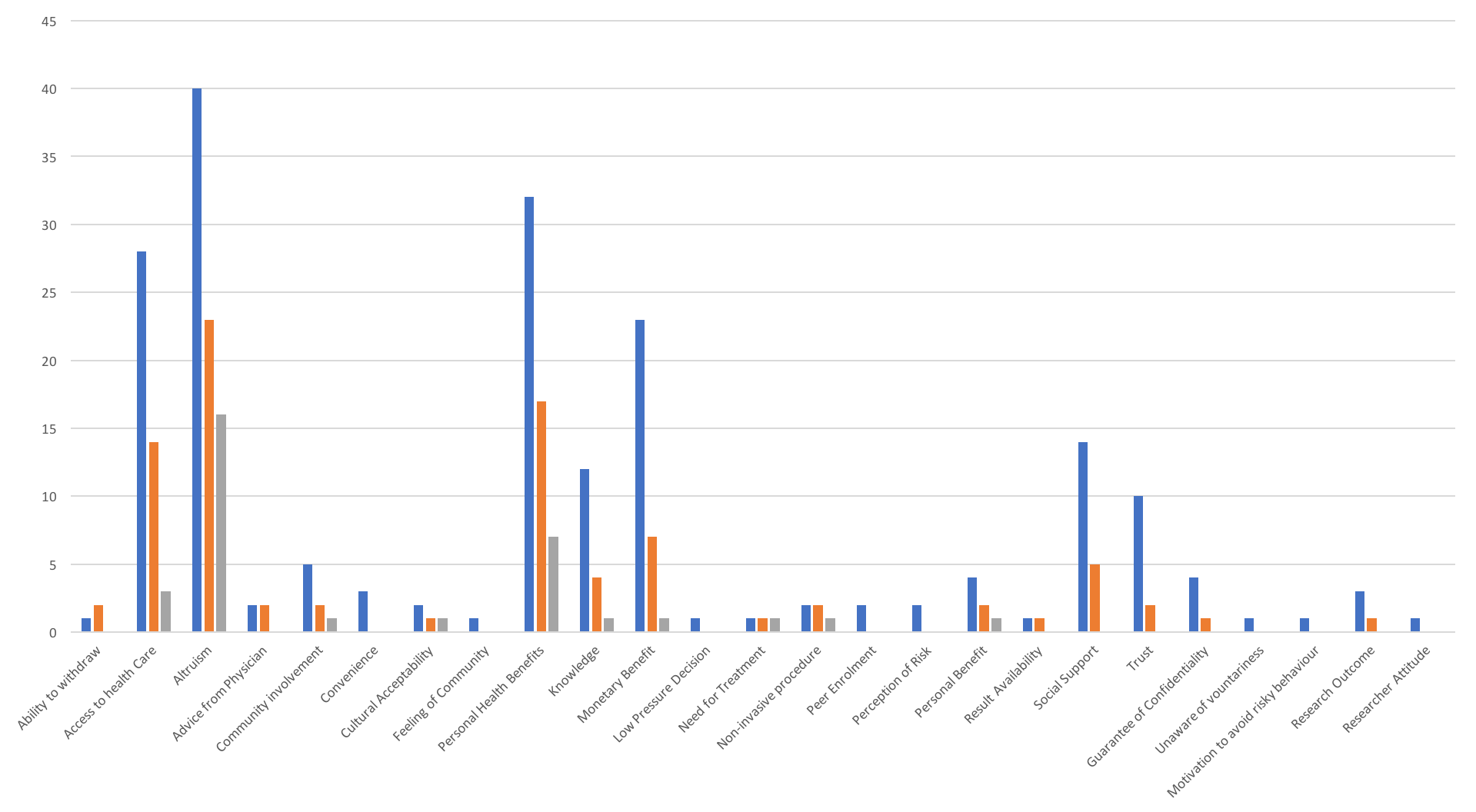


B
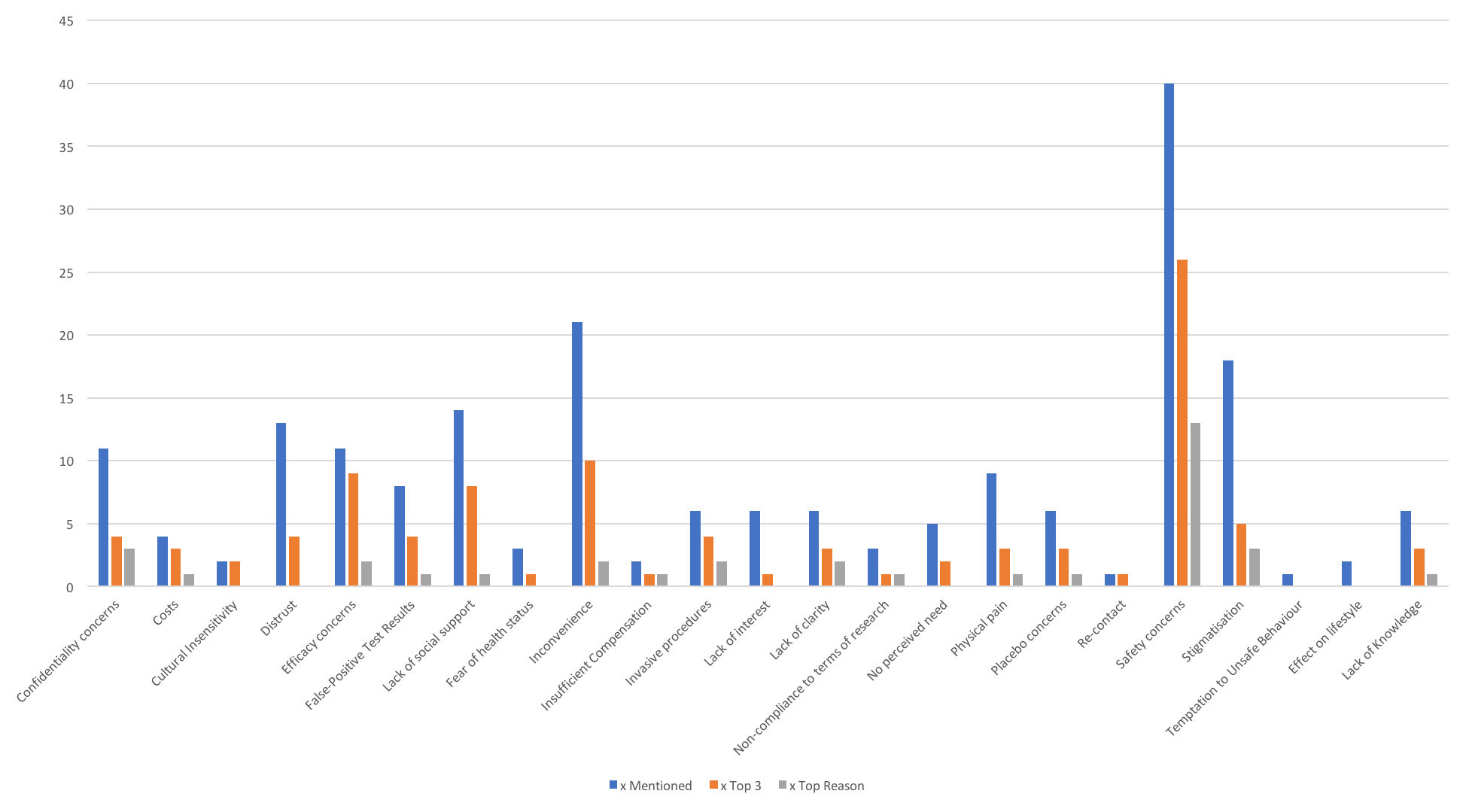


Figure S13 Ranking of Reasons for Participation (Graph A) and Non-Participation (Graph B) – Hypothetical Studies

### 
